# Asymmetric Cascade Aza-Henry/Lactamization Reaction in the Highly Enantioselective Organocatalytic Synthesis of 3-(Nitromethyl)isoindolin-1-ones from α-Amido Sulfones

**DOI:** 10.1021/acs.joc.2c00518

**Published:** 2022-06-14

**Authors:** Lorenzo Serusi, Laura Palombi, Giovanni Pierri, Antonia Di Mola, Antonio Massa

**Affiliations:** † Dipartimento di Chimica e Biologia “A. Zambelli”, Università degli Studi di Salerno, Via Giovanni Paolo II, Fisciano, Salerno 84084, Italy; ‡ Dipartimento di Scienze Fisiche e Chimiche, Università dell’Aquila, Via Vetoio, Coppito, L’Aquila 10-67100, Italy

## Abstract

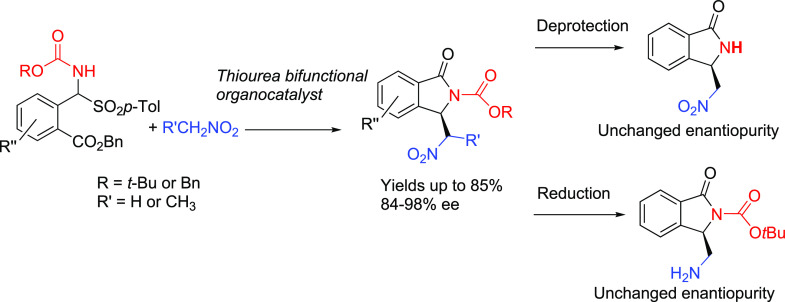

The asymmetric synthesis of novel 3-substituted isoindolinones is herein reported. A new cascade reaction was developed that consisted of the asymmetric nitro-Mannich reaction of suitable α-amido sulfones designed from 2-formyl benzoates, followed by the *in situ* cyclization of the adducts. Very high enantioselectivities, up to 98% ee, and very good yields were obtained in the presence of the readily available neutral bifunctional organocatalyst derived from *trans*-1,2-diaminocyclohexane, which is known as Takemoto’s catalyst. The investigation of the reactivity of the obtained products allowed either the selective Boc-deprotection or reduction of the nitro group, leading to further functionalized 3-substituted isoindolinones without affecting the enantiomeric purity.

## 1 Introduction

The asymmetric synthesis of 3-substituted isoindolinones is an important research area in organic chemistry because of the range of biological activities exhibited by this class of chiral heterocycles, where one enantiomer usually shows improved properties.^1,2^ Several strategies have been reported,^2−6^ which traditionally have been based on the resolution of racemates and the use of chiral auxiliaries.^2a,2b^ More recently, the development of asymmetric catalytic cascade processes based on the use of chiral metal complexes^2,3^ and organocatalytic systems^2,4−6^ has furnished very convenient tools in the asymmetric synthesis of 3-substituted isoindolinones. Despite the convenience of cascade processes,^7^ the design of *ortho*-disubstituted bifunctional aromatic substrates suitable for the organocatalytic asymmetric construction of the benzo-γ-lactam ring is rather challenging, and good levels of enantioselectivity have been achieved only in relatively few cases.^2−6^ Therefore, the development of new methods for the asymmetric construction of these heterocycles is still a research area of paramount interest.

**Scheme 1:**
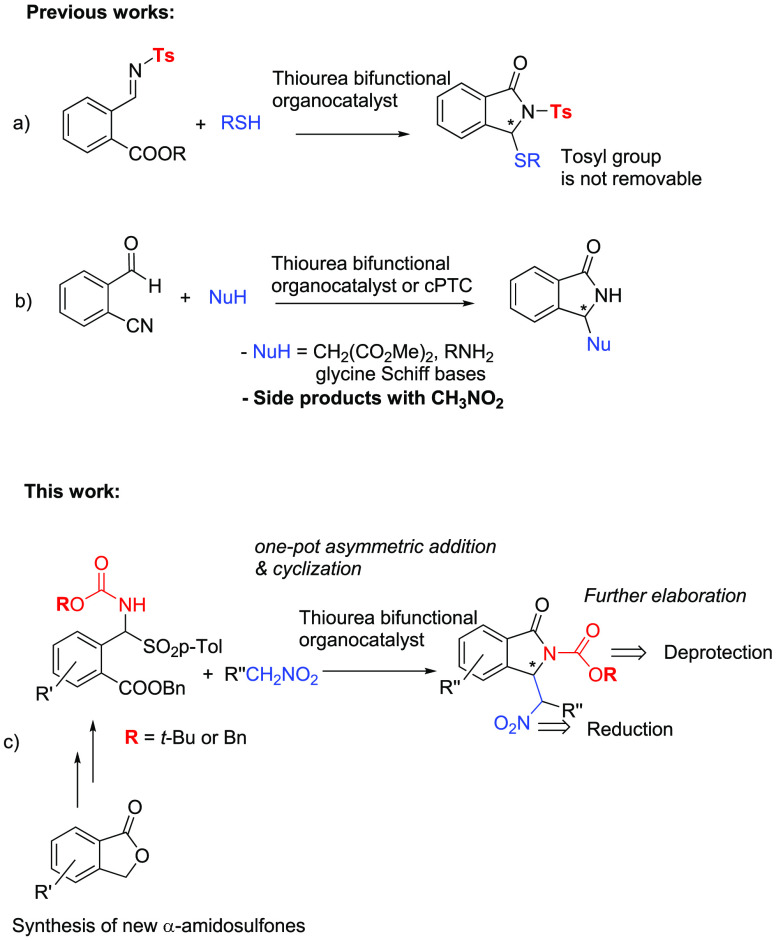
Literature *versus* The Methods Described in the Present Work

Very recently, our group developed an organocatalytic addition of thiols to *N*-tosylimine derived from 2-formylbenzoate that led to the highly enantioselective synthesis of isoindolinones-3-*N*,*S*-acetals (Scheme 1a).^6^*N*-Tosylimine of 2-formylbenzoate has also been used in the asymmetric synthesis of 3-aryl-substituted isoindolinones via arylation reactions in the presence of chiral Rh(I) or Cu(I) catalysts.^3e,3f^ Although the tosyl group proved to be essential for success in these protocols, any attempts to remove it led to very disappointing results (Scheme 1a).^3e,3f,6^ To improve the versatility of the synthetic strategies that lead to 3-substituted isoindolinones, we also attempted the syntheses of different starting materials bearing *N*-Boc and *N*-Cbz protecting groups. So far, the alternative synthesis of imines of 2-formylbenzoate bearing *N*-Boc or *N*-Cbz has never been reported in the literature.^3e,3f,6^

Over the past few years, the nucleophilic addition reaction to imines generated *in situ* from α-amido sulfone has become a very important strategy in organic chemistry and asymmetric catalysis for the synthesis of functionalized chiral amines and related nitrogen-containing compounds.^8^ The easy access and high stability of *N*-carbamoyl-α-amidosulfones guarantee several practical advantages, the first of which is avoiding the use of the respective preformed imines, which can be rather unstable or difficult to isolate. The formation of the imines from α-amidosulfones can be easily carried out *in situ* in the presence of an inorganic base. In combination with chiral phase-transfer catalysts (cPTC), this strategy has been widely explored in asymmetric catalysis for the synthesis of functionalized chiral amines, as notably described in the seminal works of Herrera,^8a^ Bernardi,^8a^ and Palomo.^8b,8d^ The presence of easily removable *N*-Boc or *N*-Cbz groups is a further synthetic advantage of these methods.^8^

In principle, aromatic α-amidosulfones functionalized at the *ortho*-position with a reactive carboxylic group could be suitable substrates for lactam formation in the synthesis of heterocyclic compounds such as 3-substituted isoindolinones (Scheme 1c). The presence of carbamoyl groups, which can be easily removed, can be particularly useful in the further manipulation of these heterocycles. To our knowledge, such *ortho*-substituted aromatic α-amido sulfones have never been reported.

Our attention was focused on the asymmetric aza-Henry reaction, also known as nitro-Mannich reaction, since it is an important tool in the synthesis of active pharmaceutical ingredients (API) and other biologically active compounds.^9^ However, isoindolinones monosubstituted at the 3-position with a nitromethyl side chain have never been reported (Scheme 1).^10,11^ Cascade reactions of 2-cyanobenzaldehyde with active methylene compounds or heteronucleophiles are important tools in the synthesis of 3-monosubstituted isoindolinones (Scheme 1b).^12^ We also developed asymmetric versions of this reaction promoted by both organocatalysts and chiral phase-transfer catalysts.^5^ However, in the presence of nitromethane, only decomposition products were observed.^10,11^

## 2 Results and Discussion

**Figure 1:**
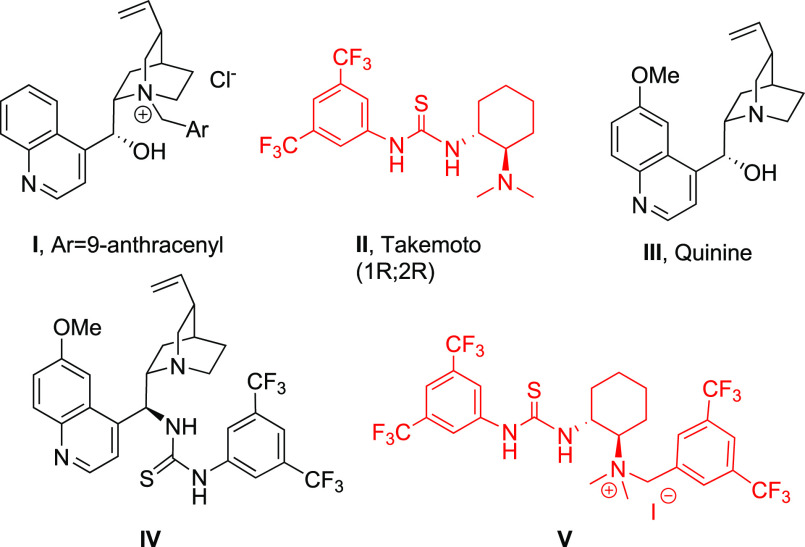
Catalytic systems used in this investigation.

To investigate the feasibility of the proposed strategy, we first demonstrated that novel α-amido sulfones derived from 2-formyl benzoates could also be easily synthesized and purified in a >2 mmol scale process. With the starting materials in hand, we then focused on the possibility of developing an asymmetric cascade aza-Henry/cyclization reaction in the presence of readily available chiral phase-transfer catalysts under the conditions of Table 1. Even though cPTCs like **I** (Figure 1), derived from Cinchona alkaloids, are widely used to accomplish asymmetric reactions of α-amido sulfones in combination with an excess amount of a strong base,^8^ we did not detect the formation of the desired cyclic product. In particular, significant decomposition of the starting materials was observed when KOH was used as base (Table 1, entry 1), along with slow reactivity in the presence of K_2_CO_3_ (Table 1, entry 2). Therefore, we turned our attention to thiourea bifunctional organocatalysts such as Takemoto’s catalyst **II**(13) in combination with an inorganic base necessary to promote *in situ* imine formation. Nicely, in the presence of K_2_CO_3_, the desired product was obtained after the addition step was performed at −20 °C for 24 h and the lactamization step was carried out via further stirring at r.t. for 48 h (Table 1, entry 3). The evolution of the entire cascade process was easily checked by TLC analysis. Stronger bases such as KOH led to decomposition products, probably due to the saponification of the ester group (Table 1, entry 4). An improvement of the enantioselectivity was progressively observed when the MeNO_2_ equivalents and the medium concentration were increased and the temperature of the addition step was decreased at −40 °C in toluene (Table 1, entries 5–8). This allowed us to obtain the 3-substituted isoindolinone in a very good yield and the excellent enantioselectivity of 96% ee (Table 1, entry 8). The reaction was less effective in DCM (Table 1, entry 7).

**Table 1:**
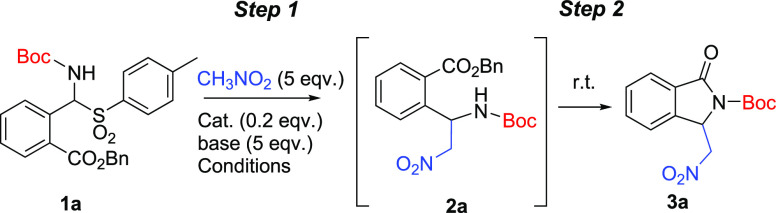
Preliminary Screening of Conditions

entry	catalyst/base	*C* (M)	solvent	*T* (°C) step 1	time (h) step 1/step 2	yield (%)^a^	ee^b^
1^c^	**I**/KOH	0.05	*m*-xylene	–20	8/21		
2^c^	**I**/K_2_CO_3_	0.05	*m*-xylene	–20	24/48		
3^c^	**II**/K_2_CO_3_	0,.5	*m*-xylene	–20	24/41	58	44
4^c^	**II**/KOH	0.05	*m*-xylene	–20	8/21		
5^c^	**II**/K_2_CO_3_	0.1	*m*-xylene	–20	27/45	83	76
6^c^	**II**/K_2_CO_3_	0.1	toluene	–40	79/89	83	88
7^c^	**II**/K_2_CO_3_	0.1	DCM	–40	38/48	68	72
8	**II**/K_2_CO_3_	0.2	toluene	–40	29/47	85	96
9	**III**/K_2_CO_3_	0.2	toluene	–40	50/90		
10	**IV**/K_2_CO_3_	0.2	toluene	–40	96/72	57	–72^d^
11	**V**/K_2_CO_3_	0.2	toluene	–40	50/48		

a Isolated yields after chromatography.

b Determined by HPLC on a chiral stationary phase.

c Used 1.5 equiv of CH_3_NO_2_.

d The opposite enantiomer was obtained.

We also tested other catalytic systems for comparison (Figure 1 and Table 1). In the presence of quinine **III**, we observed the formation of the acyclic intermediate to some extent, but the catalyst did not effectively accomplish the cyclization step. This indicates that bifunctionality and a hydrogen bond network are structural prerequisites necessary for the success of the process (Table 1, entry 9). The thiourea bifunctional organocatalyst **IV** derived from *epi*-quinine was effective, leading to the final product in a moderate yield and good ee (Table 1, entry 10). A bifunctional chiral ammonium salt **V**, which was structurally related to Takemoto’s catalyst and widely used in asymmetric catalysis,^5b,12,14^ confirmed the inaptitude of phase transfer catalysis toward the reaction investigated in the present study, as it led to decomposition products (Table 1, entry 11).

**Figure 2:**
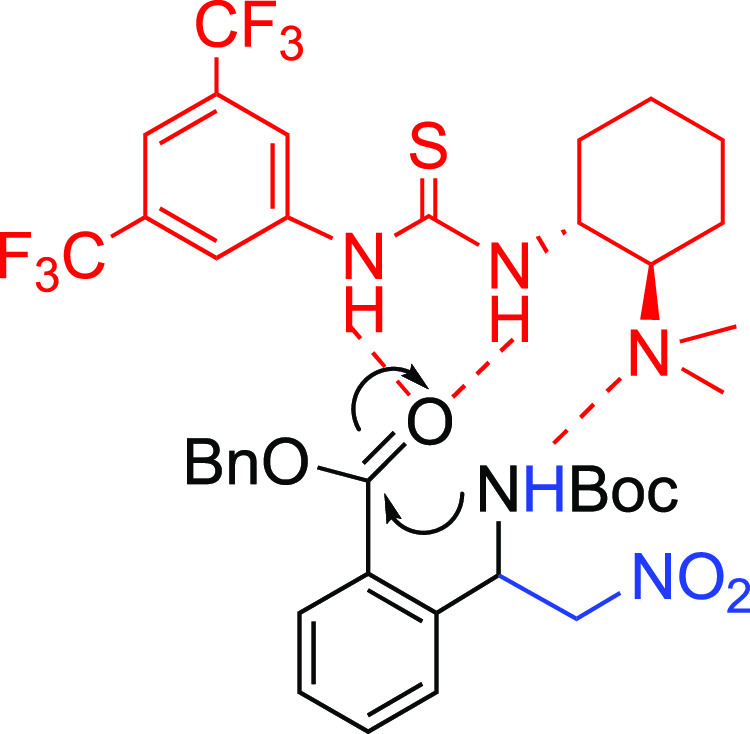
Proposed TS for the cyclization of intermediate **2a**.

Control experiments further shed some light on the process investigated herein. In particular, the synthesis of the racemate, attempted under several conditions (utilizing inorganic bases, such as K_2_CO_3_ or CsCO_3_, or organic bases, such as Et_3_N, in different solvents, such as toluene or acetonitrile, at different temperatures from 0 to +50 °C) gave sluggish outcomes. If we observed the formation of the acyclic intermediate to some extent, the cyclization was achieved with difficulty in low yields under the conditions used, and decomposition products were mainly observed at the end of the experiments. This trend was also observed with the catalysts **I**, **III**, and **V**, indicating that bifunctionality is important not only to carry out the addition step in a highly enantioselective manner but also for the cyclization. This may be due to a transition state (TS) in which the bifunctional organocatalyst can be involved both in the carbonyl activation and the amine deprotonation, as shown in Figure 2.

In the presence of Takemoto’s catalyst **II**, we were able to isolate and analyze the acyclic intermediate **2a** by purifying the reaction mixture directly at the end of step 1 of the process using chromatography. It showed good stability and the same level of enantiopurity with respect to the cyclic product.

*N*-Cbz amidosolfone **1b** led to *N*-Cbz isoindolinone **3b** in a good yield and a very high enantioselectivity (Table 2, entry 2), which was only slightly lower than that of Boc-derivative **3a** (Table 2, entry 1). The enantioselective synthesis of **3a** and **3b** was also scaled up to 1.0 and 0.19 mmol of the respective α-amido sulfones, respectively, leading to similar levels of enantiopurity and yields. This was particularly useful for the investigation of the secondary reactivity of the products (see next section). The nature of the ester group is also important, since the methyl ester of the α-amido sulfone was less effective than the benzyl ester in terms of the yield and enantioselectivity (Table 2, entry 3).

**Table 2:**
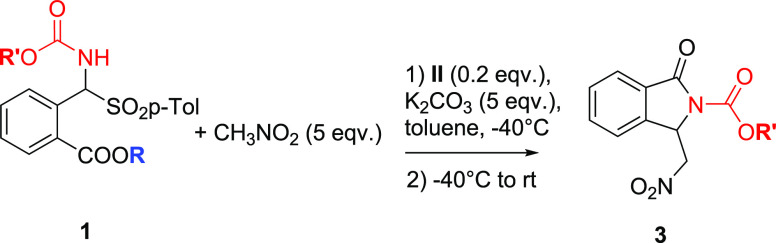
Effect of α-Amido Sulfone Structural Features

entry	R	R′	time (h) step 1/step 2	**3**, yield (%)^a^	ee^b^
1	Bn	*t*-Bu	29/47	**3a**, 85	96
2	Bn	Bn	24/46	**3b**, 78	92
3	Me	*t*-Bu	32/48	**3a**, 56	76

a Isolated yields after chromatography.

b Determined by HPLC on a chiral stationary phase.

The scope of the method was next analyzed in the presence of novel α-amido sulfones with further substituents on the aromatic ring (Table 3), whose syntheses have been detailed in the Supporting Information. This is an important goal since *N*-tosylimines derived from 2-formylbenzoates bearing further substituents on the aromatic ring are difficult to obtain and, to our knowledge, have never been reported (Scheme 1a).^3e,3f,6^ With these substrates in hand, a library of new chiral isoindolinones has been obtained under the optimized conditions with moderate to good yields and excellent levels of enantioselectivity, up to 98% ee, regardless of the presence of halogens, electron-donating groups, or a further phenyl substituent in different positions of the aromatic ring.

**Table 3:**
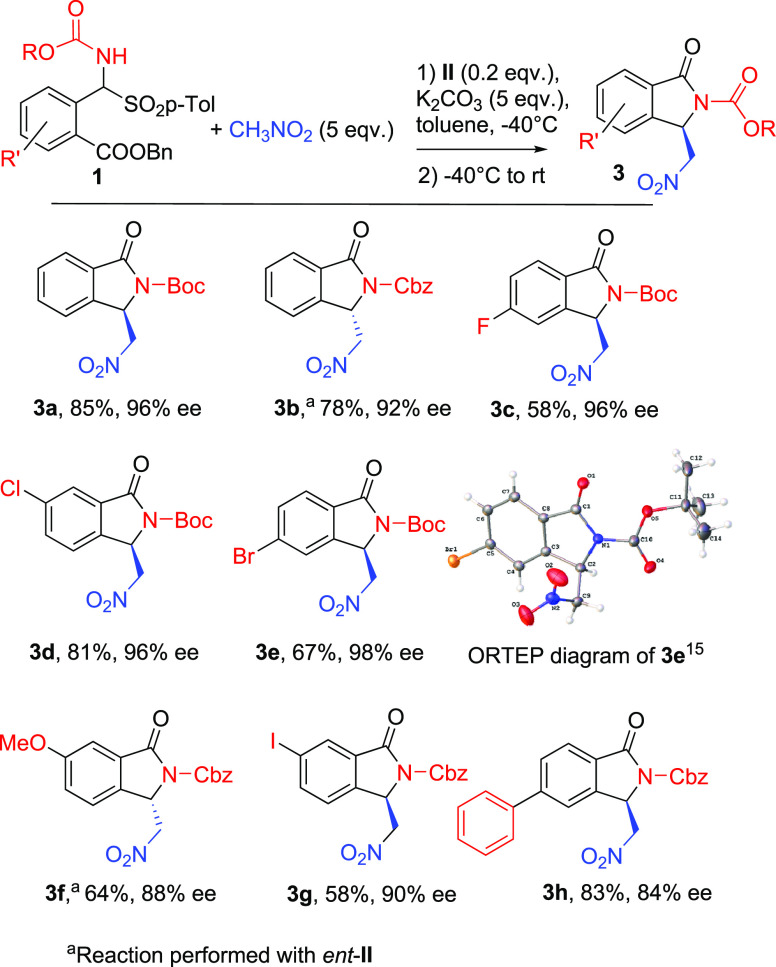
Scope of the Reaction

a Reaction performed with *ent*-II.

The use of *N*-Cbz α-amido sulfones (substrates **1f**, **1g**, and **1h**) was necessary because the synthesis of the analogous *N*-Boc α-amido sulfones was not achieved. In the presence of all the *N*-Cbz derivatives, very good results were also obtained, with ee values only slightly lower than those of the *N*-Boc isoindolinones. The synthesis of **1** bearing a strong electron-withdrawing NO_2_ group was not achieved.

As anticipated, the racemate synthesis gave generally poor results. In the presence of K_2_CO_3_ in acetonitrile at rt, we were able to isolate the racemates in low yields (see the Supporting Information for details) in some cases. When the racemic products were not isolated at all, the enatiopurity was analyzed comparing chromatograms of the opposite enantiomers obtained in the presence of the readily available *ent*-**II**, which also gave reproducible reactions with comparable efficiencies. The absolute configuration (AC) was determined to be (*R*)^15^ by the X-ray crystallographic analysis of a single crystal of **3e** when **II** (*R,R* configuration) was used (see the Supporting Information for further details), which was extended by analogy to the other derivatives.

**Scheme 2:**
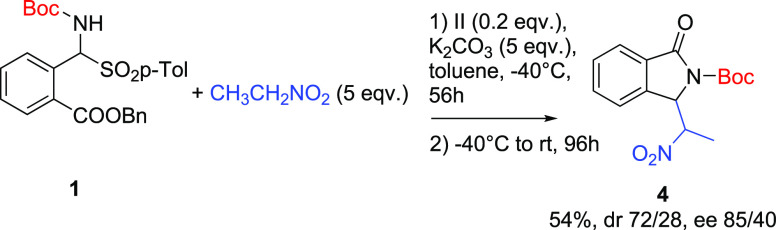
Reaction with Nitroethane

Several trials were performed employing nitroethane. We observed that the system was more complex to handle due to the formation of mixtures of diastereomers at all stages of the process. However, at longer reaction times we were able to get the final product with a moderate level of diastereoselectivity and a very good ee of 85% for the major diastereomer (Scheme 2).

**Scheme 3:**
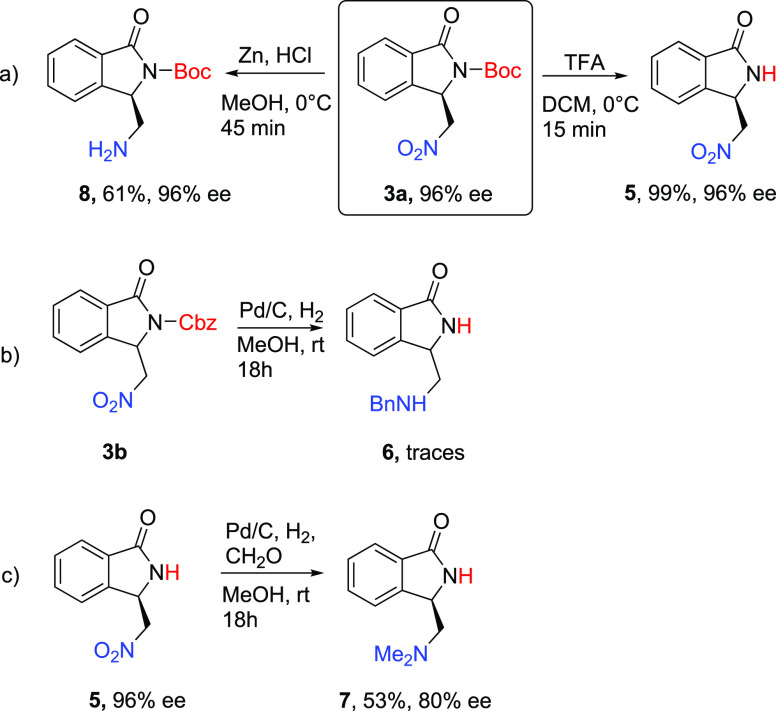
Useful Synthetic Transformations of *N*-Carbamoyl-3-(nitromethyl)isoindolin-1-ones

The reactivity of the highly enantioenriched **3** was investigated, focusing on *N*-deprotection and the reduction of the nitro group (Scheme 3). This would lead to new platforms useful for further elaborations such as selective *N*-alkylation or *N*-arylation following to methods previously described for other isoindolinones.^12a,12c^ The treatment of **3a** with TFA led to the free-NH 3-(nitromethyl)isoindolinone **5**(16) in a quantitative yield and unchanged enantiopurity (Scheme 3a). The selective reduction of the nitro group required more effort. Preliminary trials, performed in the presence of Pd/C and H_2_ on **3b** and **5**, were not satisfying even though this procedure was employed in literature on *N*-Boc amino nitro derivatives.^8d^ In an attempt to combine the reduction and deprotection of **3b** we observed the formation of a complex mixture of products in which we detected the *N*-benzylated product **6** in a low yield and a low purity (Scheme 3b). On the other hand, the presence of formaldehyde, even in traces, led to the *N,N*-dimethylated product **7** when starting material **5** was used (Scheme 3c) because of further reductive amination. Under these conditions, a significant erosion of the enantiopurity was also observed (Scheme 3c). The loss of the enantiopurity led us to focus on a different method using Zn and a low amount of HCl in MeOH to try to develop a more challenging reduction of **3a** without affecting the carbamoyl group (Scheme 3a). Nicely, under the tested conditions, we observed the reproducible selective reduction of the nitro group, which led to the very interesting *N*-Boc-lactam of 3-(aminomethyl)isoindolinone **8** in a good yield and an unchanged enantiopurity (Scheme 3a). The reaction was also successfully performed on both *ent*-**3a** and the racemate.

## 3 Conclusion

The highly enantioselective synthesis of novel 3-substituted isoindolinones bearing an unprecedented nitromethyl side chain has been reported in good yields and up to 98% ee. For this purpose, a new asymmetric cascade aza-Henry/cyclization reaction was developed to design suitable α-amido sulfones from 2-formyl benzoates. The use of bifunctional neutral organocatalysts such as Takemoto’s catalyst is of paramount importance not only to achieve a high enantioselectivity in the addition step but also to accomplish the lactamization of the acyclic intermediate. Several control experiments were performed to corroborate these findings. Boc-deprotection and the selective reduction of the nitro group were also carried out, leading to other novel isoindolinonic platforms in good yields and unchanged enantiomeric purity.

## 4 Experimental Section

### 4.1 General Methods

Unless otherwise noted, all chemicals, reagents, and solvents for the performed reactions were commercially available and used without further purification. In particular, phthalide, 5-bromo-phthalide, 3-hydroxyphthalide, and 6-chloro-3-hydroxyphthalide are commercially available. The other phthalides and 2-formylbenzoate esters **a**, **d**, **f**, and **g** are known and were prepared according to literature procedures, as detailed in the Supporting Information. Benzyl 2-formylbenzoates **c**, **e**, and **h** are new: their spectroscopic data and copies of their NMR spectra are also reported in the Supporting Information. Catalysts **I**–**IV** are commercially available. Catalyst **V** was generously supplied by Prof. Mario Waser, Institute of Organic Chemistry, University of Linz, Austria.

All reactions were monitored by thin-layer chromatography (TLC) on precoated silica gel plates (0.25 mm) and visualized by fluorescence at 254 nm. Flash chromatography was carried out using silica gel 60 (70–230 mesh, Merck, Darmstadt, Germany). Yields are given for isolated products that showed one spot on a TLC plate and had no detectable impurities in the NMR spectrum. The NMR spectra were recorded on Bruker DRX 600, 400, and 300 MHz spectrometers (600 MHz ^1^H and 125 MHz ^13^C, 400 MHz ^1^H and 100.6 MHz ^13^C, and 300 MHz ^1^H and 75.5 MHz ^13^C). The internal reference was set to the residual solvent signals (δH 7.26 ppm and δC 77.16 ppm for CDCl_3_). The ^13^C NMR spectra were recorded under broad-band proton-decoupling. Spectra are reported only for unknown compounds. The following abbreviations are used to indicate the multiplicity in the NMR spectra: s = singlet, d = doublet, t = triplet, q = quartet, dd = doublet of doublets, m = multiplet, and brs = broad signal. Coupling constants (*J*) are quoted in Hertz. High-resolution mass spectra (HRMS) were acquired using a Bruker SolariX XR Fourier transform ion cyclotron resonance mass spectrometer (Bruker Daltonik GmbH, Bremen, Germany) equipped with a 7T refrigerated actively shielded superconducting magnet. For the ionization of the samples, electrospray ionization (ESI) or MALDI was applied. IR spectra were recorded on a IR Bruker Vertex 70v spectrometer.

### 4.2 General Procedure for the Synthesis of the α-Amido Sulfones Derivatives **1a**–**1i**

To a rapidly stirred suspension of *t*-butyl-carbamate (257 mg, 1.0 equiv, 2.2 mmol) and *p*-toluenesulfinic acid sodium salt (563 mg, 1.3 equiv, 2.9 mmol) in methanol/water (2:1, 2.5 mL/1.25 mL) were added benzyl 2-formylbenzoate **a** (528 mg, 1.0 equiv, 2.2 mmol) and formic acid (210 μL) at room temperature. The reaction mixture was vigorously stirred for three days and then filtered. The resulting white solid was washed with water and ether and then dried in *vacuo* to yield the pure sulfone, which was then used without further purification.

#### 4.2.1.1 Benzyl-2-(((*tert*-butoxycarbonyl)amino)(tosyl)methyl)benzoate (**1a**)

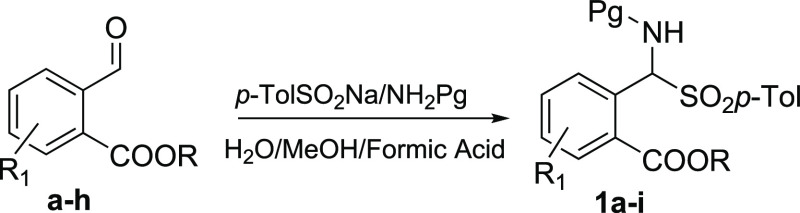



Following the general procedure, the title compound was obtained as white solid in a 92% yield (1.00 g). Mp 124–126 °C. ^1^H NMR (400 MHz, CDCl_3_) δ 8.07 (d, *J* = 6.9 Hz, 1H), 7.81 (d, *J* = 8.6 Hz, 2H), 7.65–7.50 (m, 5H), 7.48–7.30 (m, 5H), 6.22 (d, *J* = 10.4 Hz, 1H), 5.67 (d, *J* = 10.4 Hz, 1H), 5.41 (s, 2H), 2.41 (s, 3H), 1.27 (s, 9H). ^13^C{^1^H} NMR (100 MHz, CDCl_3_) δ 167.1, 153.8, 145.0, 135.9, 134.4, 132.7, 132.6, 131.6, 131.4, 130.9, 129.9, 129.5, 129.4, 128.9, 128.8, 128.6, 81.1, 69.8, 67.5, 28.2, 21.9. HRMS (ESI-FT ICR) *m*/*z* calcd for C_27_H_29_NO_6_S [M + K]^+^ 534.1353, found 534.1334.

#### 4.2.1.2 Benzyl-2-((((benzyloxy)carbonyl)amino)(tosyl)methyl)benzoate (**1b**)

Following the general procedure using 0.83 mmol **a** and benzyl-carbamate (1.0 equiv, 0.83 mmol), the title compound was obtained as a white solid in an 80% yield (350 mg). Mp 124–126 °C. ^1^H NMR (300 MHz, CDCl_3_) δ 8.06 (d, *J* = 7.1 Hz, 1H), 7.72 (d, *J* = 8.1 Hz, 2H), 7.60–7.33 (m, 12H), 7.25–7.20 (m, 3H), 6.57 (d, *J* = 11.1 Hz, 1H), 5.40 (s, 2H), 4.96 (q, *J* = 11.7 Hz, 2H), 2.41 (s, 3H). ^13^C{^1^H} NMR (75 MHz, CDCl_3_) δ 167.1, 154.9, 145.1, 135.9, 135.8, 134.2, 132.7, 131.5, 130.9, 129.8, 129.6, 129.3, 128.8, 128.6, 28.6, 128.5, 128.4, 70.6, 67.6, 21.9. HRMS (ESI-FT ICR) *m*/*z* calcd for C_30_H_27_NO_6_S [M + K]^+^ 568.1196, found 568.1185.

#### 4.2.1.3 Benzyl-2-(((*tert*-butoxycarbonyl)amino)(tosyl)methyl)-4-fluorobenzoate (**1c**)

Following the general procedure using 0.90 mmol **c**, the title compound was obtained as a white solid in a 94% yield (430 mg). Mp 144–146 °C. ^1^H NMR (400 MHz, CDCl_3_) δ 8.14–8.10 (m, 1H), 7.83–7.75 (m, 3H), 7.48 (d, *J* = 6.9 Hz, 2H), 7.42–7.32 (m, 5H), 7.17–7.12 (m, 1H), 6.03 (d, *J* = 10.6 Hz, 1H), 5.40 (s, 2H), 2.42 (s, 3H), 1.27 (s, 9H). ^13^C{^1^H} NMR (100 MHz, CDCl_3_) δ 166.0, 153.7, 145.2, 135.8, 135.3, 134.3, 134.0, 133.9, 129.9, 129.4, 128.8, 128.6, 128.5, 127.0, 116.5, 116.3, 81.3, 69.2, 67.5, 28.1, 21.8. HRMS (ESI-FT ICR) *m*/*z* calcd for C_27_H_28_FNO_6_S [M + Na]^+^ 536.1519, found 536.1505.

#### 4.2.1.4 Benzyl-2-(((*tert*-butoxycarbonyl)amino)(tosyl)methyl)-5-chlorobenzoate (**1d**)

Following the general procedure using 1.0 mmol **d**, the title compound was obtained as a white solid in a 72% yield (378 mg). Mp 136–138 °C. ^1^H NMR: (300 MHz, CDCl_3_) δ 8.03 (s, 1H), 7.78 (d, *J* = 6.6 Hz, 2H), 7.56–7.48 (m, 2H), 7.44–7.31 (m, 7H), 6.12–6.08 (m, 1H), 5.41 (s, 2H), 2.42 (s, 3H), 1.26 (s, 9H). ^13^C{^1^H} NMR (100 MHz, CDCl_3_) δ 165.9, 153.7, 145.2, 135.9, 135.5, 134.2, 132.6, 132.4, 131.3, 130.6, 130.2, 129.9, 129.4, 128.8, 128.7, 128.6, 81.3, 69.4, 67.8, 28.1, 21.8. HRMS (ESI-FT ICR) *m*/*z* calcd for C_27_H_28_ClNO_6_S [M + Na]^+^ 552.1224, found 552.1223.

#### 4.2.1.5 Benzyl-4-bromo-2-(((*tert*-butoxycarbonyl)amino)(tosyl)methyl)benzoate (**1e**)

Following the general procedure using 1.6 mmol **e**, the title compound was obtained a white solid in a 72% yield (660 mg). Mp 141–143 °C. ^1^H NMR (400 MHz, CDCl_3_) δ 7.94 (d, *J* = 9.0 Hz, 1H), 7.81 (d, *J* = 7.5 Hz, 2H), 7.70–7.66 (m, 2H), 7.60 (d, *J* = 9.0 Hz, 1H), 7.48–7.32 (m, 6H), 6.05 (d, *J* = 10.5 Hz, 1H), 5.40 (s, 2H), 2.42 (s, 3H), 1.27 (s, 9H). ^13^C{^1^H} NMR (75 MHz, CDCl_3_) δ 166.3, 153.7, 145.3, 135.5, 134.2, 133.8, 132.6, 132.3, 129.9, 129.7, 129.4, 128.8, 128.6, 128.6, 127.5, 81.3, 69.1, 67.6, 28.1, 21.8. HRMS (ESI-FT ICR) *m*/*z* calcd for C_27_H_28_BrNO_6_S [M + Na]^+^ 596.0718, found 596.0722.

#### 4.2.1.6 Benzyl-2-((((benzyloxy)carbonyl)amino)(phenylsulfonyl)methyl)-5-methoxybenzoate (**1f**)

Following the general procedure using 0.90 mmol **f** and benzyl-carbamate (1.0 equiv, 0.90 mmol), the title compound was obtained in a 67% yield (320 mg). The title compound slowly decomposed in solution at rt, therefore the ^13^C NMR spectrum was not recorded. Decomposition was also observed during the MS analysis. ^1^H NMR (400 MHz, CDCl_3_) δ 7.70–7.69 (m, 2H), 7.57 (s, 1H), 7.47–7.33 (m, 8H), 7.26–7.20 (m, 4H), 7.07 (s, 2H), 6.57 (d, *J* = 8.8 Hz, 1 H), 5.39 (s, 2H), 4.95 (q, *J* = 13.4 Hz, 2 H), 3.84 (s, 3H), 2.40 (s, 3H).

#### 4.2.1.7 Benzyl-2-((((benzyloxy)carbonyl)amino)(tosyl)methyl)-5-iodobenzoate (**1g**)

Following the general procedure using 0.50 mmol **g** and benzyl-carbamate (1.0 equiv, 0.50 mmol), the title compound was obtained a white solid in a 61% yield (200 mg). Mp 143–145 °C. ^1^H NMR (300 MHz, CDCl_3_) δ 8.36 (s, 1H), 7.90 (d, *J* = 8.0 Hz, 1H), 7.70 (d, *J* = 8.0 Hz, 2H), 7.55–7.29 (m, 9H), 7.26–7.20 (m, 4H), 6.46 (d, *J* = 10.7 Hz, 1H), 5.40 (s, 2H), 4.95 (q, *J* = 12.0 Hz, 2H), 2.41 (s, 3H). ^13^C{^1^H} NMR (100 MHz, CDCl_3_) δ 165.7, 154.7, 145.4, 141.6, 140.0, 135.8, 135.5, 134.0, 132.5, 131.2, 130.7, 129.9, 129.3, 129.0, 128.9, 128.8, 128.7, 128.5, 128.4, 127.5, 95.7, 70.2, 67.7, 22.0. HRMS (ESI-FT ICR) *m*/*z* calcd for C_30_H_26_INO_6_S [M + Na]^+^ 678.0423, found 678.0422.

#### 4.2.1.8 Benzyl-3-((((benzyloxy)carbonyl)amino)(phenylsulfonyl)methyl)-[1,1′-biphenyl]-4-carboxylate (**1h**)

Following the general procedure using 0.32 mmol **h** and benzyl-carbamate (1.0 equiv, 0.32 mmol), the title compound was obtained in a 60% yield (114 mg). The title compound slowly decomposed in solution at rt, therefore the ^13^C NMR spectrum was not recorded. Decomposition was also observed during the MS analysis. ^1^H NMR (300 MHz, CDCl_3_) δ 8.14 (d, *J* = 7.6 Hz, 2 H), 7.77.-7.64 (m, 6H), 7.57 (d, *J* = 7.6 Hz, 3H), 7.49–7.33 (m, 9H), 7.21 (d, *J* = 8.3 Hz, 2H), 6.68 (d, *J* = 11.4 Hz, 1H), 5.43 (s, 2H), 4.96 (q, *J* = 12.5 Hz, 2H), 2.41 (s, 3H).

#### 4.2.1.9 Methyl-2-(((*tert*-butoxycarbonyl)amino)(tosyl)methyl)benzoate (**1i**)

Following the general procedure using 4.2 mmol methyl 2-formylbenzoate, the title compound was obtained as a white solid in a 92% yield (1.6 g). Mp 156–158 °C. ^1^H NMR (400 MHz, CDCl_3_) δ 8.04 (d, *J* = 6.5 Hz, 1H), 7.85 (d, *J* = 7.8 Hz, 2H), 7.60–7.49 (m, 4H), 7.34 (d, *J* = 7.8 Hz, 2H), 6.25 (d, *J* = 10.4 Hz, 1H), 5.73 (d, *J* = 10.4 Hz, 1H), 3.96 (s, 3H), 2.42 (s, 3H), 1.26 (s, 9H). ^13^C{^1^H} NMR (75 MHz, CDCl_3_) δ 167.7, 153.8, 145.1, 132.5, 131.3, 131.0, 129.8, 129.4, 81.0, 69.9, 52.8, 28.1, 21.8. HRMS (ESI-FT ICR) *m*/*z* calcd for C_21_H_25_NO_6_S [M + Na]^+^ 442.1300, found 442.1288.

### 4.3 Typical Procedure for the Asymmetric Synthesis of *N*-Carbamoyl-3-substituted Isoindolin-1-ones **3a**–**3h**

In an ACE tube, α-amido sulfones **1a**–**1i** (1 equiv, 0.08 mmol,), K_2_CO_3_ (55 mg, 5 equiv, 4 mmol), nitromethane (21 μL, 5 equiv, 0.4 mmol), and organocatalyst **II** (20 mol %) were stirred in at −40 °C in toluene (0.4 mL) until the starting material was completely converted to the intermediate. Then, the reaction mixture was allowed to slowly warm to room temperature, and stirring was continued until the intermediate disappeared (48 h). The mixture was directly purified by flash chromatography on silica gel.

#### 4.3.1 (*R*)-Benzyl-2-(1-((*tert*-butoxycarbonyl)amino)-2-nitroethyl)benzoate (**2a**)

Starting from 0.08 mmol α-amido sulfone **1a**, the compound was obtained as a very viscous oil (27 mg, 85%) after the direct purification on silica gel of the product from step 1 of the general reaction (hexane/ethyl acetate 5:1). [α]_D_^18^= +2.3 (*c* = 0.40, CHCl_3_). ^1^H NMR (300 MHz, CDCl_3_) δ 8.07 (d, *J* = 7.7 Hz, 1H), 7.55–7.36 (m, 8H), 6.13–6.07 (m, 2H), 5.38 (s, 2H), 4.93–4.78 (m, 2H), 1.41 (s, 9H). ^13^C{^1^H} NMR (100 MHz, CDCl_3_) δ 167.2, 154.9, 139.3, 135.5, 133.5, 132.1, 129.3, 128.9, 128.7, 128.5, 128.2, 127.1, 80.5, 78.8, 67.5, 51.7, 28.4. HRMS (MALDI-FT ICR) *m*/*z* calcd for C_21_H_24_N_2_O_6_ [M + Na]^+^ 423.1526, found 423.1542. HPLC analysis: Chiralpak OD-H column, *n*-hexane/*i*-PrOH 70:30, 0.8 mL/min, *t*_R_ = 7.2 min, *t*_S_ = 8.2 min. 96% ee.

#### 4.3.2 (*R*)-*tert*-Butyl-1-(nitromethyl)-3-oxoisoindoline-2-carboxylate (**3a**)

Following the general procedure using 0.08 mmol α-amido sulfone **1a**, the title compound was obtained as a very viscous oil in an 85% yield (20 mg) after chromatography on silica gel (hexane/ethyl acetate 5:1). [α]_D_^18^= +31.3 (*c* = 0.75, CHCl_3_). ^1^H NMR (300 MHz, CDCl_3_) δ 7.92 (d, *J* = 7.7 Hz, 1H), 7.70 (t, *J* = 7.8 Hz, 1H), 7.58 (t, *J* = 7.5 Hz, 1H), 7.48 (d, *J* = 8.1 Hz, 1H), 5.65 (dd, *J*_1_ = 6.0 Hz, *J*_2_ = 3.8 Hz,, 1H), 5.10 (dd, *J*_1_ = 12.0 Hz, *J*_2_ = 3.8 Hz, 1H), 4.77 (dd, *J*_1_ = 12.0 Hz, *J*_2_ = 6.3 Hz, 1H), 1.61 (s, 9H). ^13^C{^1^H} NMR (100 MHz, CDCl_3_) δ 165.4, 150.2, 140.9, 134.6, 131.0, 130.2, 125.7, 122.9, 84.9, 76.5, 57.1, 28.2. IR (neat) 1783, 1747, 1699, 1562, 1333, 756 cm^–1^. HRMS (MALDI-FT ICR) *m*/*z* calcd for C_14_H_16_N_2_O_5_ [M + K]^+^ 331.0691, found 331,0710. HPLC analysis: Chiralpak OD-H column, *n*-hexane/*i*-PrOH 70:30, 0.8 mL/min, *t*_S_ = 13.4 min, *t*_R_ = 21.0 min. 96% ee. The reaction was also scaled to 1.0 mmol (495 mg) **1a** under the same conditions, yielding 78% **3a** (228 mg, 96% ee).

#### 4.3.3 (*S*)-Benzyl-1-(nitromethyl)-3-oxoisoindoline-2-carboxylate (**3b**)

Following the general procedure using 0.08 mmol α-amido sulfone **1b**, the title compound was obtained as a very viscous oil in a 78% yield (20 mg) after chromatography on silica gel (hexane/ethyl acetate 5:1). [α]_D_^18^= −32.8 (*c* = 0.75, CHCl_3_). ^1^H NMR (400 MHz, CDCl_3_) δ 7.94 (d, *J* = 7.4 Hz, 1H), 7.70 (t, *J* = 7.4 Hz, 1H), 7.59 (t, *J* = 7.4 Hz, 1H), 7.49 (s, 3H), 7.41–7.34 (m, 3H), 5.67 (m, 1H), 5.42 (s, 2H), 5.12 (d, *J* = 9.0 Hz, 1H), 4.84 (d, *J* = 9.0 Hz, 1H). ^13^C{^1^H} NMR (100 MHz, CDCl_3_) δ 165.1, 151.8, 140.9, 135.0, 134.8, 130.4, 130.3, 128.9, 128.8, 128.3, 125.9, 123.0, 76.0, 69.0, 57.2. IR (neat) 1740, 1654, 1546, 1290, 788 cm^–1^. HRMS (MALDI-FT ICR) *m*/*z* calcd for C_17_H_14_N_2_O_5_ [M + K]^+^ 365.0534, found 365.0520. HPLC analysis: Chiralpak IC column, *n*-hexane/*i*-PrOH 70:30, 1 mL/min, *t*_R_ = 27.8 min, *t*_S_ = 29.9 min. 92% ee. The reaction was also scaled to 0.19 mmol (100 mg) **1b**, yielding 78% **3b** (51 mg, 92% ee).

#### 4.3.4 (*R*)-*tert*-Butyl-5-fluoro-3-(nitromethyl)-1-oxoisoindoline-2-carboxylate (**3c**)

Following the general procedure using 0.08 mmol α-amido sulfone **1c**, the title compound was obtained as a very viscous oil in a 58% yield (14 mg) after chromatography on silica gel (hexane/ethyl acetate 5:1). [α]_D_^18^= +66.9 (*c* = 0.35, CHCl_3_). ^1^H NMR: (400 MHz, CDCl_3_) δ 7.94–7.90 (m, 1H), 7.29–7.27 (m, 1H), 7.19 (d, *J* = 7.5 Hz, 1H) 5.61 (m, 1H), 5.10 (d, *J* = 10.2 Hz, 1H), 4.80 (d, *J* = 10.0 Hz, 1H), 1.61 (s, 9H). ^19^F NMR (400 MHz, chloroform-*d*, 298 K, ppm) δ −101.4. ^13^C{^1^H} NMR (100 MHz, CDCl_3_) δ 166.7 (*J*_C–F_ = 259.1 Hz), 164.2, 150.0, 143.4 (*J*_C–F_ = 9.9 Hz), 128.1 (*J*_C–F_ = 9.9 Hz), 126.8, 118.4 (*J*_C–F_ = 19.9 Hz), 110.6 (*J*_C–F_ = 29.9 Hz), 85.1, 76.1, 56.6, 28.2. IR (neat) 1770, 1605, 1564, 1551, 756 cm^–1^. HRMS (ESI-FT ICR) *m*/*z* calcd for C_14_H_15_FN_2_O_5_ [M + Na]^+^ 333.0863, found 333.0856. HPLC analysis: Chiralpak OD-H column, *n*-hexane/*i*-PrOH 70:30, 0.8 mL/min, *t*_S_ = 13.4 min, *t*_R_ = 21.4 min. 96% ee.

#### 4.3.5 (*R*)-*tert*-Butyl-5-chloro-1-(nitromethyl)-3-oxoisoindoline-2-carboxylate (**3d**)

Following the general procedure using 0.08 mmol α-amido sulfone **1d**, the title compound was obtained as a very viscous oil in an 81% yield (21 mg) after chromatography on silica gel (hexane/ethyl acetate 5:1). [α]_D_^18^= +46.3 (*c* = 0.55, CHCl_3_). ^1^H NMR (300 MHz, CDCl_3_) δ 7.87 (s, 1H), 7.64 (d, *J* = 8.1 Hz, 1H), 7.43 (d, *J* = 8.1 Hz, 1H), 5.60–5.59 (m, 1H), 5.10 (dd, *J*_1_ = 12.0 Hz, *J*_2_ = 3.8 Hz, 1H), 4.80 (dd, *J*_1_ = 12.0 Hz, *J*_2_ = 6.0 Hz, 1H), 1.61 (s, 9H). ^13^C{^1^H} NMR (75 MHz, CDCl_3_) δ 164.0, 150.0, 139.0, 136.8, 134.7, 132.5, 125.6, 124.3, 85.3, 76.1, 56.8, 28.2. IR (neat) 1772, 1561, 1351, 761 cm^–1^. HRMS (MALDI-FT ICR) *m*/*z* calcd for C_14_H_15_ClN_2_O_5_ [M + Na]^+^ 351.0537, found 351.0519. HPLC analysis: Chiralpak OD-H column, *n*-hexane/*i*-PrOH 70:30, 0.8 mL/min, *t*_S_ = 14.8 min, *t*_R_ = 18.9 min. 96% ee.

#### 4.3.6 (*R*)-*tert*-Butyl-5-bromo-3-(nitromethyl)-1-oxoisoindoline-2-carboxylate (**3e**)

Following the general procedure using 0.08 mmol α-amido sulfone **1e**, the title compound was obtained as a very viscous oil in a 67% yield (20 mg) after chromatography on silica gel (hexane/ethyl acetate 5:1). [α]_D_^18^= +32.8 (*c* = 0.70, CHCl_3_). ^1^H NMR (300 MHz, CDCl_3_) δ 7.76–7.67 (m, 3H); 5.60 (m, 1H), 5.08 (d, *J* = 12.0 Hz, 1H), 4.81 (dd, *J*_1_ = 12.0 Hz, *J*_2_ = 6.2 Hz, 1H), 1.60 (s, 9H). ^13^C{^1^H} NMR (75 MHz, CDCl_3_) δ 164.4, 150.0, 142.6, 133.9, 129.7, 129.6, 127.0, 126.4, 85.2, 76.0, 56.6, 28.8. IR (neat) 1745, 1712, 1546, 1330, 745 cm^–1^. HRMS (ESI-FT ICR) *m*/*z* calcd for C_14_H_15_BrN_2_O_5_ [M + Na]^+^ 393.0062, found 393.0053. HPLC analysis: Chiralpak OD-H column, *n*-hexane/*i*-PrOH 70:30, 0.8 mL/min, *t*_S_ = 14.7 min, *t*_R_ = 21.9 min. 98% ee.

#### 4.3.7 (*S*)-Benzyl-5-methoxy-1-(nitromethyl)-3-oxoisoindoline-2-carboxylate (**3f**)

Following the general procedure using 0.08 mmol α-amido sulfone **1f**, the title compound was obtained as a very viscous oil in a 64% yield (18 mg) after chromatography on silica gel (hexane/ethyl acetate 5:1). [α]_D_^18^= −40.3 (*c* = 0.40, CHCl_3_). ^1^H NMR (300 MHz, CDCl_3_) δ 7.50 (d, *J* = 7.1 Hz, 2H), 7.43–7.35 (m, 5H), 7.24 (dd, *J*_1_ = 2.3 Hz, *J*_2_ = 6.4 Hz, 1H), 5.6 (dd, *J*_1_ = 6.3 Hz, *J*_2_ = 3.8 Hz, 1H), 5.42 (s, 2H), 5.09 (dd, *J*_1_ = 12.0 Hz, *J*_2_ = 3.7 Hz, 1H), 4.77 (dd, *J*_1_ = 12.1 Hz, *J*_2_ = 6.3 Hz, 1H), 3.87 (s, 3H). ^13^C{^1^H} NMR (100 MHz, CDCl_3_) δ 165.2, 161.5, 151.7, 135.0, 133.0, 131.8, 128.9, 128.8, 128.3, 124.0, 123.4, 107.9, 76.2, 68.9, 56.8, 56.0. IR (neat) 1726, 1710, 1551, 1499, 1343, 781 cm^–1^. HRMS (ESI-FT ICR) *m*/*z* calcd for C_18_H_16_N_2_O_6_ [M + Na]^+^ 379.0906, found 379.0895. HPLC analysis: Chiralpak OD-H column, *n*-hexane/*i*-PrOH 60:40, 1 mL/min, *t*_S_ = 20.4 min, *t*_R_ = 30.6 min. 88% ee.

#### 4.3.8 (*R*)-Benzyl-5-iodo-1-(nitromethyl)-3-oxoisoindoline-2-carboxylate (**3g**)

Following the general procedure using 0.08 mmol α-amido sulfone **1g**, the title compound was obtained as a very viscous oil in a 58% yield (21 mg) after chromatography on silica gel (hexane/ethyl acetate 5:1). [α]_D_^18^= +11.8 (*c* = 0.80, CHCl_3_). ^1^H NMR (400 MHz, CDCl_3_) δ 8.26 (s, 1H), 8.00 (d, *J* = 7.1 Hz, 1H), 7.48 (d, *J* = 7.1 Hz, 1H), 7.40–7.37 (m, 4H), 7.24 (s, 1H), 5.60 (m, 1H), 5.41 (bs, 2H), 5.09 (d, *J* = 11.9 Hz, 1H), 4.81 (dd, *J*_1_ = 12.5 Hz, *J*_2_ = 6.0 Hz, 1H). ^13^C{^1^H} NMR (100 MHz, CDCl_3_) δ 163.4, 151.5, 143.6, 140.1, 134.9, 134.8, 132.3, 128.9, 128.9, 128.4, 124.7, 95.7, 75.5, 69.2, 57.0. IR (neat) 1745, 1555, 1381, 1118, 775 cm^–1^. HRMS (ESI-FT ICR) *m*/*z* calcd for C_17_H_13_IN_2_O_5_ [M + Na]^+^ 474.9767, found 474.9759. HPLC analysis: Chiralpak AS-H column, *n*-hexane/*i*-PrOH 80:20, 0.8 mL/min, *t*_S_ = 66.2 min, *t*_R_ = 71.1 min. 90% ee.

#### 4.3.9 (*R*)-Benzyl-3-(nitromethyl)-1-oxo-5-phenylisoindoline-2-carboxylate (**3h**)

Following the general procedure using 0.08 mmol α-amido sulfone **1h**, the title compound was obtained as a very viscous oil in an 83% yield (26 mg) after chromatography on silica gel (hexane/ethyl acetate 5:1). [α]_D_^18^= +12.4 (*c* = 0.40, CHCl_3_). ^1^H NMR (300 MHz, CDCl_3_) δ 7.99 (d, *J* = 8.3 Hz, 1H), 7.79 (d, *J* = 8.3 Hz,1H), 7.64–7.57 (m, 3H), 7.52–7.35 (m, 8H), 5.72 (dd, *J*_1_ = 6.5 Hz, *J*_2_ = 3.6 Hz, 1H), 5.43 (s, 2H), 5.17 (dd, *J*_1_ = 12.5 Hz, *J*_2_ = 3.8 Hz, 1H), 4.85 (dd, *J*_1_ = 12.0 Hz, *J*_2_ = 6.6 Hz, 1H). ^13^C{^1^H} NMR (150 MHz, CDCl_3_) δ 164.8, 151.7, 148.2, 141.5, 139.3, 134.9, 129.5, 129.2, 128.9, 128.7, 128.6, 128.2, 127.6, 126.1, 121.4, 75.9, 68.9, 57.0. IR (neat) 1765, 1699, 1674, 1533, 1302, 781 cm^–1^. HRMS (ESI-FT ICR) *m*/*z* calcd for C_23_H_18_N_2_O_5_ [M + K]^+^ 441.0847, found 441.0859. HPLC analysis: Chiralpak IC column, *n*-hexane/*i*-PrOH 60:40, 1 mL/min, t_S_ = 24.6 min, *t*_R_ = 30.1 min. 84% ee.

### 4.4 *tert*-Butyl-1-(1-nitroethyl)-3-oxoisoindoline-2-carboxylate (**4**)

In an ACE tube, α-amido sulfone **1a** (27 mg, 1 equiv, 0.08 mmol), K_2_CO_3_ (55 mg, 5 equiv, 0.4 mmol), nitroethane (28 μL, 5 equiv, 0.4 mmol), and organocatalyst II (20 mol %) were stirred in at −40 C° in toluene (0.4 mL) until the starting material was completely converted to the intermediate. Then, the reaction mixture was allowed to slowly warm to room temperature, and the stirring was continued until the intermediate disappeared (96 h). The crude was directly purified by flash chromatography on silica gel (hexane/ethyl acetate 7:3). Yield: 14 mg, 54% (solid). dr: 72/28. ^1^H NMR mixture of diastereomers (400 MHz, CDCl_3_) δ 7.93 (d, *J* = 7.3 Hz, 1H, major diastereomer), 7.72–7.67 (m, 1H, major diastereomer), 7.59–7.55 (m, 2H, major diastereomer), 5.89 (bs, 1H, minor diastereomer), 5.77 (bs, 1H, major diastereomer), 5.50–5.49 (m, 1H, minor diastereomer), 5.05–5.04 (m, 1H, major diastereomer), 1.62 (s, 9H, minor diastereomer), 1.57 (s, 9H, major diastereomer), 1.42 (d, *J* = 6.4 Hz, 3H, major diastereomer), 1.06 (d, *J* = 6.4 Hz, 3H, minor diastereomer). ^13^C{^1^H} NMR mixture of diastereomers (100 MHz, CDCl_3_) δ 165.9 (×2), 150.1 (×2), 140.7, 139.3, 134.5, 134.4, 131.0, 130.2, 125.8, 125.6, 123.9, 122.9, 85.1, 84.8, 83.3, 81.0, 62.0, 60.7, 28.3, 28.0, 12.3, 11.1. HRMS (ESI-FT ICR) *m*/*z* calcd for C_15_H_18_N_2_O_5_ [M + K]^+^ 345.0847, found 345.0852. HPLC analysis: Chiralpak OD-H column, *n*-hexane/*i*-PrOH 80:20, 0.8 mL/min, *t*_minor,d1_= 8.3 min, t_2_major,d1__= 8.8 min, *t*_minor,d2_= 17.1 min, t_2_major,d2__= 18.9 min. 85% ee_1_ and 40% ee_2_.

### 4.5 (*R*)-3-(Nitromethyl)isoindolin-1-one (**5**)

In a round-bottom flask, **3a** (20 mg, 1 equiv, 0.07 mmol) was stirred in CH_2_Cl_2_ (300 μL) and CF_3_COOH (150 μL) for 10–15 min at 0 °C. The mixture was diluted with DCM and water. To the mixture was added 1 M NaOH at rt under stirring until the pH reached 7, then the aqueous phase was further extracted twice with DCM. The combined organic layers were dried over Na_2_SO_4_, filtered and evaporated *in vacuo*. The crude mixture was purified by flash chromatography on silica gel (hexane/ethyl acetate 1:1). Yield: 14 mg, 99% (white solid). Mp 142–144 °C. [α]_D_^18^= −31.6 (*c* = 0.15, CHCl_3_). ^1^H NMR (300 MHz, CDCl_3_) δ 7.92 (d, *J* = 7.7 Hz, 1H), 7.65–7.58 (m, 2H), 7.45 (d, *J* = 7.7 Hz, 1H), 6.97 (s, 1H), 5.28 (d, *J* = 10.3 Hz, 1H), 4.91 (dd, *J*_1_ = 12.3, *J*_2_ = 3.1 Hz, 1H), 4.38 (dd, *J*_1_ = 12.3, *J*_2_ = 10.2 Hz, 1H). ^13^C{^1^H} NMR (75 MHz, DMSO-d_6_) δ 170.0, 143.4, 133.1, 132.4, 129.5, 124.1, 123.7, 78.2, 54.2. IR (KBr) 3180, 1718, 1546 cm^–1^. HRMS (ESI-FT ICR) *m*/*z* calcd for C_9_H_8_N_2_O_3_ [M + Na]^+^ 215.0427, found 215.0424. HPLC analysis: Chiralpak OD-H column, *n*-hexane/*i*-PrOH 80:20, 0.6 mL/min, *t*_R_ = 35.2 min, *t*_S_ = 40.1 min. 96% ee.

### 4.6 (*R*)-3-((Dimethylamino)methyl)isoindolin-1-one (**7**)

A suspension of **5** (12 mg, 1 equiv, 0.06 mmol) and 10% Pd/C (20 mol %) in methanol (1 mL) was stirred under a hydrogen atmosphere for 18 h at room temperature. The mixture was filtered on Celite and eluted with MeOH/DCM. The organic layer was then evaporated under reduced pressure, and the crude product was purified by flash chromatography on silica (chloroform/methanol 95:5). Yield: 6 mg, 53% (very viscous oil). [α]_D_^18^= −54.7 (*c* = 0.30, CHCl_3_). ^1^H NMR (300 MHz, CDCl_3_) δ 7.85 (d, *J* = 7.2 Hz, 1H), 7.57–7.42 (m, 3H), 6.76 (s, 1H), 4.65 (dd, *J*_1_ = 11.1 Hz, *J*_2_ = 3.5 Hz, 1H), 2.67 (dd, *J*_1_ = 12.0 Hz, *J*_2_ = 3.8 Hz, 1H), 2.36 (m, 6 + 1H). ^13^C{^1^H} NMR (75 MHz, CDCl_3_) δ 170.5, 145.7, 132.4, 131.9, 128.6, 124.2, 122.7, 64.4, 54.8, 45.9 (2C, −NMe_2_). IR (neat) 2787, 1695, 1468, 1303, 748 cm^–1^. HRMS (MALDI-FT ICR) *m*/*z* calcd for C_11_H_14_N_2_O [M + Na]^+^ 213.0998, found 213.0996. HPLC analysis: Chiralpak IC column, *n*-hexane/*i*-PrOH 60:40, 1 mL/min, *t*_S_ = 11.0 min, *t*_R_ = 11.9 min. 80% ee.

### 4.7 (*R*)-*tert*-Butyl-1-(aminomethyl)-3-oxoisoindoline-2-carboxylate (**8**)

To a solution of compound **3a** (20 mg, 1 equiv, 0.07 mmol,) in methanol (500 μL) in a round-bottom flask were added zinc dust (23 mg, 4 equiv, 0.3 mmol) and 6 M HCl (90 μL) at 0 °C. The mixture was stirred at room temperature for 40–45 min. Then, the mixture was basified with 1 M NaOH until the pH reached 7 and extracted twice with ethyl acetate. Combined organic layers were then dried over Na_2_SO_4_ and evaporated *in vacuo*. Purification by flash chromatography on silica gel (chloroform/methanol 95:5) gave the product. Yield: 11 mg, 61% (very viscous oil). [α]_D_^18^= +15.1 (*c* = 0.40, CHCl_3_). ^1^H NMR (400 MHz, CDCl_3_) δ 7.91 (d, *J* = 7.5 Hz, 1H), 7.66 (t, *J* = 7.5 Hz, 1H), 7.52–7.51 (m, 2H), 5.09 (bs, 1H), 3.50–3.46 (m, 1H), 3.31 (m, 1H), 1.61 (s, 9H). ^13^C{^1^H} NMR (100 MHz, CDCl_3_) δ 167.3, 150.5, 143.5, 134.1, 131.7, 129.1, 125.3, 122.7, 83.7, 62.2, 44.1, 28.3. IR (neat) 2979, 2933, 1772, 1708, 1335, 1151 cm^–1^. HRMS (MALDI-FT ICR) *m*/*z* calcd for C_14_H_18_N_2_O_3_ [M + Na]^+^ 285.1209, found 285.1200. HPLC analysis: Chiralpak OD-H column, *n*-hexane/*i*-PrOH 80:20, 0.8 mL/min, *t*_R_ = 10.1 min, *t*_S_ = 11.9 min. 96% ee.

## Supporting Information Available

^1^H and ^13^C{^1^H} NMR spectra and IR spectra (PDF)
FAIR data, including the primary NMR FID files, for compounds **c**, **e**, **h**, **1**, **2a**, **3**, **4**, **5**, **7**, and **8** (ZIP)

## Accession Codes

CCDC 2143635 contains the supplementary crystallographic data for this paper. These data can be obtained free of charge via www.ccdc.cam.ac.uk/data_request/cif, or by emailing data_request@ccdc.cam.ac.uk, or by contacting The Cambridge Crystallographic Data Centre, 12 Union Road, Cambridge CB2 1EZ, UK; fax: +44 1223 336033.
