# Supplementary material for: Asymmetric Cascade Aza-Henry/Lactamization Reaction in the Highly Enantioselective Organocatalytic Synthesis of 3-(Nitromethyl)isoindolin-1-ones from α-Amido Sulfones
Source: J Org Chem. 2022 Jun 14;87(13):8420–8. doi: 10.1021/acs.joc.2c00518 (PMC9490826; doi:10.1021/acs.joc.2c00518)

Supporting Information

Asymmetric cascade Aza-Henry/lactamization reaction in the highly enantioselective organocatalytic synthesis of 3-(nitromethyl)isoindolin-1-ones from α-amido sulfones.

Lorenzo Serusi,[a] Laura Palombi,[b] Giovanni Pierri,[a] Antonia Di Mola,[a,*] and Antonio Massa [a,*]

[a] Dipartimento di Chimica e Biologia "A. Zambelli", Università degli Studi di Salerno, Via Giovanni Paolo II, 84084-Fisciano (SA), Italy.
[b] Dipartimento di Scienze Fisiche e Chimiche, Università dell'Aquila, Via Vetoio, Coppito, L'Aquila 10-67100, Italy;

*Corresponding Authors email: amassa@unisa.it, adimola@unisa.it

Table of Contents

General information S3

Experimental procedures for intermediates synthesis S3

General procedure for the synthesis of the α-amido sulfones S9

General procedure for the synthesis of *N*-protected 3-substituted isoindolin-1-ones S9

Crystallographic data S11

References S13

Copies of ^{1}H NMR and ^{13}C NMR spectra of intermediates and α-amido sulfones S14

Copies of ^{1}H NMR, ^{13}C NMR spectra and HPLC traces of enantioenriched isoindolin-1-ones S27

Copies of IR spectra of selected isoindolin-1-ones S51

General Information

Unless otherwise noted, all chemicals, reagents and solvents for the performed reactions are commercially available and were used without further purification. Phthalide, 5-bromo-phthalide, 3-hydroxyphthalide and 6-chloro-3-hydroxyphthalide were purchased from Fluorochem. Other phthalides and 2-formylbenzoate esters **a-h** were prepared according to literature procedures.[1-7] Catalysts I-IV are commercially available. Catalyst V was gently supplied by Prof. Mario Waser, Institute of Organic Chemistry, University of Linz, Austria. All the reactions were monitored by thin layer chromatography (TLC) on precoated silica gel plates (0.25 mm) and visualized by fluorescence quenching at 254 nm. Flash chromatography was carried out using silica gel 60 (70–230 mesh, Merck, Darmstadt, Germany). Yields are given for isolated products showing one spot on a TLC plate and no impurities were detectable in the NMR spectrum. The NMR spectra were recorded on Bruker DRX 600, 400, and 300 MHz spectrometers (600 MHz, ^{1}H, 150 MHz, ^{13}C; 400 MHz, ^{1}H, 100 MHz; ^{13}C, 300 MHz, ^{1}H, 75 MHz, ^{13}C). Internal reference was set to the residual solvent signals (δ_H 7.26 ppm, δ_C 77 ppm for $CDCl_3$ and δ_H 2.50 ppm, δ_C 39 ppm for DMSO-*d*$_6$). The ^{13}C NMR spectra were recorded under broad-band proton-decoupling. Spectra are reported only for unknown compounds. The following abbreviations are used to indicate the multiplicity in NMR spectra: s-singlet, d-doublet, t-triplet, q-quartet, dd-doublet of doublets, m-multiplet, brs-broad signal. Coupling constants (J) are quoted in Hertz. High resolution mass spectra (HRMS) were acquired using a Bruker SolariX XR Fourier transform ion cyclotron resonance mass spectrometer (Bruker Daltonik GmbH, Bremen, Germany) equipped with a 7T refrigerated actively-shielded superconducting magnet. For ionization of the samples electrospray ionization (ESI) or MALDI was applied.

Experimental procedures

Procedures for the synthesis of 6-iodophthalide

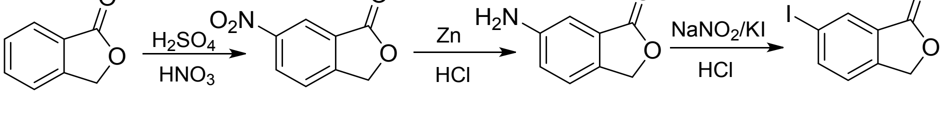

Synthesis of 6-nitrophthalide: Phthalide (2.0 g, 1.0 eq., 15.0 mmol) was dissolved in fuming nitric acid (2.0 mL) and concentrated sulfuric acid (2.3 mL) at 0 °C. The ice bath was then removed and the reaction stirred for 12 h. The reaction was then poured into an ice-H_2O bath and collected via filtration to give the crude product as a white solid. Recrystallisation from Hexane/$CHCl_3$ gave the title compound as white crystals (Yield= 1.630 mg, 61%). **^{1}H NMR** (400 MHz, $CDCl_3$): δ 8.77 (d, *J*= 1.8 Hz, 1H), 8.59 (d, *J*= 8.3 Hz, 1H), 7.72 (d, *J*= 8.4 Hz, 1H), 5.46 (s, 2H). Mp and the obtained spectroscopic data were found in agreement with literature.[1]

Synthesis of 6-aminophthalide: To a solution of 6-nitrophthalide (1.0 g, 1 eq., 5.58 mmol) in methanol (33.0 mL) Zn (1.450 g, 4 eq., 22.3 mmol) and 37% HCl (6.25 mL) were added dropwise at 0° (ice bath) and the mixture stirred for 2 h. The mixture was then basified with sodium hydroxide and filtered. The aqueous layer was extracted with Ethyl acetate two times, dried in *vacuo* to yield the corresponding 6-aminophthalide as yellow solid. (Yield= 830 mg, 99%). **^{1}H NMR** (600 MHz, DMSO-d_6): δ 7.24 (d, J= 7.8 Hz, 1H), 6.98 (dd, J_1= 8.4, J_2= 1.2 Hz, 1H), 6.92 (s, 1H), 5.46 (s, 2H), 5.18 (s, 2H). Mp and the obtained spectroscopic data were found in agreement with literature.[2]

Synthesis of 6-iodophthalide: In an ice bath a solution of $NaNO_2$ (610 mg, 1.2 eq., 6.5 mmol) in H_2O (1.2 mL) was added dropwise to a suspension of 6-aminophthalide (800 mg, 1.0 eq., 5.4 mmol) in 37% HCl (3.5 mL) and the resulting mixture was stirred for 25 min at 0°C. Then, a solution of KI (4.5 g, 5.0 eq., 27.0 mmol) in H_2O (4.6 mL) was added dropwise. The mixture was stirred at room temperature for 18 h. Then, the aqueous suspension was extracted with Ethyl acetate and the combined organic layers were washed with saturated aq. $Na_2S_2O_3$ and brine (14.0 mL), dried over Na_2SO_4 and concentrated in *vacuo*. The residue was purified by silica gel column chromatography eluting with $CHCl_3$ to obtain the product as white solid. (Yield= 900 mg, 65%). **^{1}H NMR** (300 MHz, $CDCl_3$) δ 8.26 (d, J= 1.5 Hz, 1H), 7.99 (dd, J_1= 8.0 Hz, J_2= 1.5 Hz, 1H), 7.30 (d, J= 8.0 Hz, 1 H), 5.29 (s, 2 H). Mp and the obtained spectroscopic data were found in agreement with literature.[3]

Procedure for the synthesis of 6-methoxyphthalide

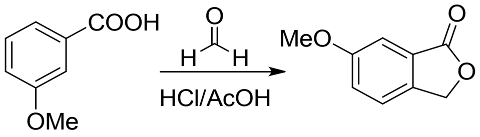

To a solution of 3-methoxybenzoic acid (1.520 g, 1.0 eq., 10.0 mmol) in glacial acetic acid (5 mL), 37% HCl (7.5 mL) and 30% formaldehyde (3 mL, 4.0 eq., 40.0 mmol) were added, and the reaction mixture was stirred at 100°C for 1 h in an oil bath. After cooling, a saturated solution of $NaHCO_3$ was added until pH=7. The resulting mixture was extracted with CH_2Cl_2 twice, the combined organic layers were dried over Na_2SO_4 and concentrated under reduced pressure. The residue was purified by column chromatography on silica gel (Hexane/Ethyl acetate, 7:1) to afford 6-methoxyphthalide as a gum. (Yield= 700 mg, 43%). **^{1}H NMR** (400 MHz, $CDCl_3$) δ 7.36 (d, J= 8.4 Hz, 1H), 7.28 (d, J= 2.2 Hz, 1H), 7.23 (dd, J_1= 8.4, J_2= 2.2 Hz, 1H), 5.24 (s, 2H), 3.84 (s, 3H). The obtained spectroscopic data were found in agreement with literature.[4]

Procedure for the synthesis of 5-phenylphthalide

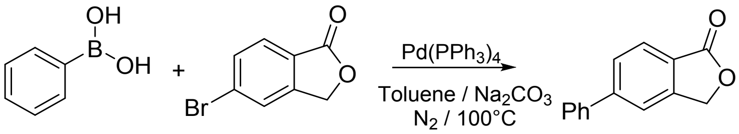

Phenylboronic acid (340 mg, 1.0 eq., 2.8 mmol), 5-bromophthalide (600 mg, 1.0 eq., 2.88 mmol) and tetrakis (triphenylphosphine)palladium 1(0) ($Pd(PPh_3)_4$) (130 mg, 4.0 mol%) were stirred in toluene (2.8 mL) and 2 M Na_2CO_3 solution (2.24 mL) at 100°C in an oil bath for 3 h under nitrogen atmosphere. Then, the mixture was extracted with Ethyl acetate three times. The combined organic layer was washed with brine, dried over anhydrous Na_2SO_4 and concentrated under reduced pressure. The crude product was purified by column chromatography on silica gel (Petroleum ether/Ethyl acetate, 8:1) affording 5-phenylphthalide as white solid (Yield= 600 mg, 99%). **^{1}H NMR** (300 MHz, $CDCl_3$) δ 7.99 (d, *J*= 7.8 Hz, 1H), 7.77 (d, *J*= 7.9 Hz, 1H), 7.65 (m, 1H), 7.45 (m, 5H), 5.38 (s, 2H). Mp and the obtained spectroscopic data were found in agreement with literature.[5]

General procedure for the synthesis of 3-hydroxy phthalide derivatives

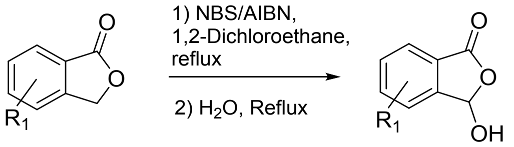

R_1	Yield of 3-OH phthalide
5-Br	82%
6-OCH_3	83%
6-I	72%
5-Ph	49%

A mixture of phthalide (1.0 eq., 2.5 mmol), *N*-bromosuccinimide (NBS, 1.1 eq., 2.75 mmol) and azobisisobutyronitrile (AIBN, 0.04 eq., 0.10 mmol) in 1,2-dichloroethane (15.0 mL) was refluxed for 1-6 h in an oil bath. The reaction mixture was kept in an ice bath for 2 h and then filtered. The solvent was removed under reduced pressure and the crude was suspended in water (7.0 mL) and the resulting mixture was refluxed for 1 h. The reaction mixture was cooled to room temperature and extracted with Ethyl acetate three times. The combined organic phases were dried and concentrated under reduced pressure to give 3-hydroxyphthalide derivatives, which are known compounds.[3,5,6]

5-bromo 3-hydroxy phthalide.[5] White solid (470 mg, 82%). **^{1}H NMR** (300 MHz, DMSO-d_6) δ 8.28 (d, *J*= 4.1 Hz, 1H), 7.96-8.00 (m, 1H), 7.63 (*d*, *J*= 7.7 Hz, 2H), 6.66 (d, *J*= 4.6 Hz, 1H).

3-hydroxy 6-methoxy phthalide.[6] Reaction carried out on 4 mmol of respective phthalide**.** White solid (597 mg, 83%). **^{1}H NMR** (400 MHz, $CDCl_3$) δ 7.54 (s, 1H), 7.29 (s, 2H), 6.53 (s, 1H), 5.57 (s, 1H), 3.88 (s, 3H)

3-hydroxy 6-iodophthalide.[3] White solid (495 mg, 72%).**^{1}H NMR** (300 MHz, DMSO-d_6) δ 8.26 (d, *J*= 7.0 Hz, 1H), 8.06-8.02 (m, 2H), 7.49 (d, *J*= 7.8 Hz, 1 H), 6.64 (d, *J*= 7.6 Hz, 1 H).

3-hydroxy 5-phenylphthalide.[5] White solid (276 mg, 49%). **^{1}H NMR** (600 MHz, $CDCl_3$) δ 7.98 (d, *J*= 7.6 Hz, 1H), 7.86 (d, *J*= 6.9 Hz, 2H), 7.66 (d, *J*= 7.6 Hz, 3H), 7.53 (t, *J*= 7.0 Hz, 2H), 7.47-7.49 (m, 1H), 6.71 (s, 1H).

Mp and the obtained spectroscopic data were found in agreement with literature.[3,5,6]

Procedure for the synthesis of 5-fluoro-3-hydroxyphthalide

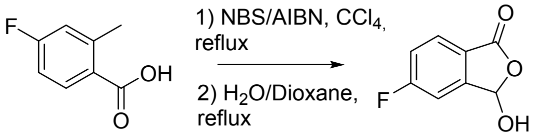

N-bromosuccinimide (1.440 mg, 2.5 eq., 8.1 mmol) and azobisisobutyronitrile (20 mg, 0.04 eq., 0.12 mmol) were added to a suspension of 4-fluoro-2-methylbenzoic acid (500 mg, 1.0 eq., 3.24 mmol) in CCl_4 (13.0 mL) and refluxed for 4-6 h in an oil bath. Then the mixture was cooled to room temperature. and filtered. The resulting organic solution was evaporated in *vacuo*, the residue dissolved in a mixture of water/dioxane (1:1, 7.5 mL/7.5 mL) and refluxed for 8 h. Then dioxane was removed under reduced pressure and the aqueous layer extracted with Ethyl acetate twice to afford the resulting 5-fluoro-3-hydroxyphthalide as white solid. (Yield= 495 mg, >99%). **^{1}HNMR** (400 MHz, DMSO-d_6) δ 8.30 (d, J= 7.3 Hz, 1H), 7.90 (dd, J_1= 8.1, J_2= 4.8 Hz, 1H), 7.54 (dd, J_1= 8.0, J_2= 1.6 Hz, 1H), 7.55-7.44 (m, 1H), 6.65 (d, J= 7.1 Hz, 1H). Mp and the obtained spectroscopic data were found in agreement with literature.[6]

General procedure for the synthesis of 2-formyl benzoate esters a-h

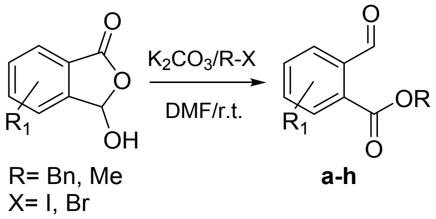

	R	R_1 in 3-OH Phthalide
**a**	Bn	H
**a-1**	Me	H
**c**	Bn	5-F
**d**	Bn	6-Cl
**e**	Bn	5-Br
**f**	Bn	6-OCH_3
**g**	Bn	6-I
**h**	Bn	5-Ph

K_2CO_3 (0.6 eq.) and Benzyl Bromide or iodomethane (1.2 eq.) were added to a suspension of 3-hydroxyphthalide (1.0 eq.) in DMF (0.5 M). The resulting mixture was stirred for 16-18 h. Then the mixture was extracted with Ethyl acetate and water and the resulting organic layer washed with brine. The organic layer was dried over Na_2CO_3 and evaporated under vacuo. The mixture was directly purified by flash chromatography on silica gel (Hexane/Ethyl acetate, 5:1) to give the resulting 2-formylbenzoate esters **a-h.** All the compounds **a**, **d**, **f**, **g** are known. The ^{1}H NMR spectra of the products are consistent with the literature data.[7] The compounds **c**, **e** and **h** are new. Their complete spectroscopic data and copies of NMR spectra are reported as supplementary materials.

Benzyl 2-formylbenzoate. (a)[7]

Following the general procedure using 6.7 mmol of the respective of 3-hydroxyphthalide, the title compound was obtained as a colorless oil in 87% yield (1.4 g).

^{1}H NMR ($CDCl_3$, 400 MHz) δ 10.60 (s, 1H), 7.99-7.96 (m, 1H), 7.93-7.90 (m, 1H), 7.63-7.58 (m, 2H), 7.44-7.34 (m, 5H), 5.40 (s, 2H).

Methyl 2-formylbenzoate. (a-1)[7]

Following the general procedure using 5.0 mmol of the respective of 3-hydroxyphthalide, the title compound was obtained as a colorless oil in 95% yield (780 mg).

^{1}H NMR ($CDCl_3$, 300 MHz): δ 10.60 (s, 1H), 7.98-7.90 (m, 2H), 7.66-7.62 (m, 2H), 3.96 (s, 3H)

Benzyl 4-fluoro-2-formylbenzoate. (c)

Following the general procedure using 2.2 mmol of the respective of 3-hydroxyphthalide, the title compound was obtained as a colorless oil in 80% yield (454 mg).

^{1}H NMR: (400 MHz, $CDCl_3$): δ 10.66 (s, 1H), 8.08 (dd, J_1= 5.5 Hz, J_2= 2.9 Hz, 1H), 7.62 (dd, J_1= 5.5 Hz, J_2= 2.9 Hz, 1H), 7.46-7.28 (m, 6H), 5.41 (s, 2H).

^{13}C NMR: (75 MHz, $CDCl_3$): δ 190.9, 165.2, 140.3, 135.3, 133.7 (J_{C-F}= 8.5 Hz), 128.9, 119.9 (J_{C-F}= 22.2 Hz), 115.4 (J_{C-F}= 22.2 Hz), 67.9.

HRMS (ESI-FT ICR): *m/z* calcd for $C_{15}H_{11}FO_3$ $[M+Na]^+$= 281.0595, found: 281.0578.

Benzyl 5-chloro-2-formylbenzoate. (d)[7]

Following the general procedure using 3.0 mmol of the respective of 3-hydroxyphthalide, the title compound was obtained as a colorless oil in 94% yield (772 mg).

^{1}H NMR ($CDCl_3$, 400 MHz) δ 10.56 (s, 1H), 7.93 (d, J = 2.0 Hz, 1H), 7.86 (d, J = 8.4 Hz, 1H), 7.56 (dd, J_1= 8.4 Hz, J_2= 2.0 Hz, 1H), 7.44-7.35 (m, 5H), 5.39 (s, 2H).

Benzyl 4-bromo-2-formylbenzoate. (e)

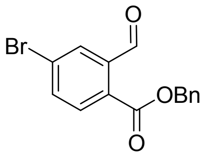

e

Following the general procedure using 2.0 mmol of the respective of 3-hydroxyphthalide, the title compound was obtained as a colorless oil in 87% yield (555 mg).

^{1}H NMR: (300 MHz, $CDCl_3$) δ 10.61 (s, 1H), 8.03 (d, *J*= 2.2 Hz, 1H), 7.88 (d, *J*= 7.2 Hz, 1H), 7.73 (dd, J_1= 7.2 Hz, J_2= 2.2 Hz, 1H), 7.44-7.37 (m, 5H), 5.40 (s, 2H).

^{13}C NMR: (75 MHz, $CDCl_3$) δ 190.8, 165.4, 138.7, 135.9, 135.2, 131.6, 130.5, 128.9, 128.7, 127.9, 68.0.

HRMS (MALDI-FT ICR): *m/z* calcd for $C_{15}H_{11}BrO_3$ $[M+Na]^+$= 340.9789, found: 340.9781.

Benzyl 2-formyl-5-methoxybenzoate. (f)[7]

Following the general procedure using 3.0 mmol of the respective of 3-hydroxyphthalide, the title compound was obtained as a colorless oil in 35% yield (283 mg).

^{1}H NMR ($CDCl_3$, 400 MHz) δ 10.54 (s, 1H), 7.82 (d, *J*= 8.0 Hz, 1H), 7.74 (s, 1H), 7.46-7.31 (m, 6H), 5.37 (s, 2H), 3.88 (s, 3H)

Benzyl 2-formyl-4-iodobenzoate. (g)[7]

Following the general procedure using 1.0 mmol of the respective of 3-hydroxyphthalide, the title compound was obtained as a colorless oil in 85% yield (311 mg).

^{1}H NMR ($CDCl_3$, 400 MHz) δ 10.57 (s, 1H), 8.33 (d, *J*=1.7 Hz, 1H), 8.01 (dd, J_1= 8.2, J_2= 1.5 Hz, 1H), 7.64 (d, *J*= 8.2 Hz, 1H), 7.48-7.34 (m, 5H), 5.43 (s, 2H).

Benzyl 3-formyl-[1,1'-biphenyl]-4-carboxylate. (h)

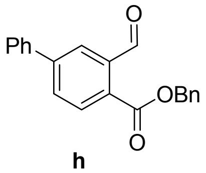

h

Following the general procedure using 1.0 mmol of the respective of 3-hydroxyphthalide, the title compound was obtained as colorless oil in 63% yield (200 mg).

^{1}H NMR: (300 MHz, $CDCl_3$) δ 10.73 (s, 1H), 8.17 (d, *J*= 1.9 Hz, 1H), 8.10 (d, *J*= 6.9 Hz, 1H), 7.85(dd, J_1= 6.9 Hz, J_2= 1.9 Hz, 1H), 7.66-7.63 (m, 2H), 7.49-7.38 (m, 8H), 5.44 (s, 2H).

^{13}C NMR: (75 MHz, $CDCl_3$) δ 192.4, 166.1, 145.5, 138.8, 137.9, 135.5, 131.5, 131.2, 130.4, 129.3, 128.9, 128.8, 128.6, 127.4, 127.0, 67.8.

HRMS (MALDI-FT ICR): *m/z* calcd for $C_{21}H_{16}O_3$ $[M+Na]^+$= 339.0991, found: 339.1027.

General procedure for the synthesis of the α-amido sulfones derivatives 1a-1i.[8]

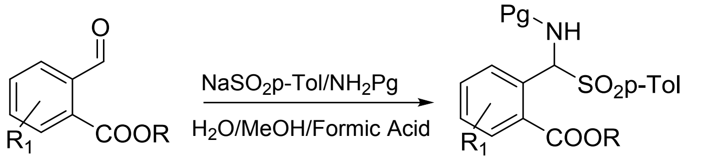

a-h

R= Bn, Me
R$_1$= H, F, Cl, Br, OMe, I, Ph
Pg= Boc,Cbz

1a (R_1=H; R=Bn; Pg= Boc)
1b (R_1=H; R=Bn; Pg=Cbz)
1c (R_1=F; R=Bn; Pg=Boc)
1d (R_1=Cl; R=Bn; Pg=Boc)
1e (R_1=Br; R=Bn; Pg=Boc)
1f (R_1=OMe; R=Bn; Pg=Cbz)
1g (R_1=I; R=Bn; Pg=Cbz)
1h (R_1=Ph; R=Bn; Pg=Cbz)
1i (R_1=H; R=Me; Pg= Boc)

2-formyl benzoate esters **a-h** (1.0 eq., using 2.2 mmol of aldehyde **a**, R= Bn, R_1= H) and formic acid (210 µL) were added at room temperature to a rapidly stirred suspension of *t*-butyl- or benzyl- carbamate (1.0 eq., 2.2 mmol) and *p*-toluenesulfinic acid sodium salt (1.3 eq., 2.9 mmol) in methanol/ water (2:1, 2.5 mL/1.25 mL). The reaction mixture was vigorously stirred for three days and then filtered. The resulting white solid was washed with water and diethyl ether and then dried in *vacuo* to yield the pure sulfone that was then used without further purification. For the other compounds the reactions were performed on 0.80-2.2 mmol range. Characterization of compounds is reported in the main text. Copies of the spectra are reported below.

General procedure for the asymmetric synthesis of *N*-protected 3-substituted isoindolin-1 ones 3a-3h

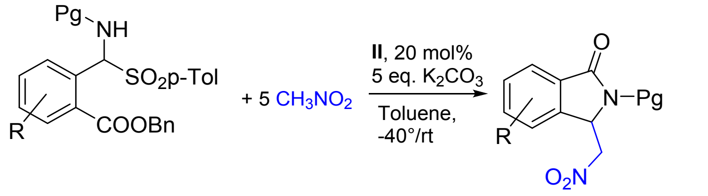

1a (R=H; Pg= Boc)
1b (R=H; Pg=Cbz)
1c (R=F; Pg=Boc)
1d (R=Cl; Pg=Boc)
1e (R=Br; Pg=Boc)
***1f** (R=OMe; Pg=Cbz)
1g (R=I; Pg=Cbz)
***1h** (R=Ph; Pg=Cbz)

3a (R=H; Pg= Boc)
3b (R=H; Pg=Cbz)
3c (R=F; Pg=Boc)
3d (R=Cl; Pg=Boc)
3e (R=Br; Pg=Boc)
3f (R=OMe; Pg=Cbz)
3g (R=I; Pg=Cbz)
3h (R=Ph; Pg=Cbz)

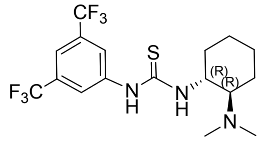

II

*Compounds employed as crude starting materials.

In an ACE tube, α-amido sulfones **1a-1i** (1.0 eq., 0.08 mmol,), K_2CO_3 (55 mg, 5.0 eq., 4 mmol), nitromethane (21 µL, 5.0 eq., 0.4 mmol) and organocatalyst **II** (20 mol%) were stirred in at -40° in toluene (0.4 mL) until starting material was all converted in the intermediate. Then, the reaction mixture was allowed to slowly warm up to room temperature and the stirring was continued until the intermediate disappeared (48 h). The

mixture was directly purified by flash chromatography on silica gel (Hexane/Ethyl acetate, 5:1). Characterization of compounds is reported in the main text. Copies of the spectra are reported below.

Racemic procedure for the synthesis of compound 3a and 3e

α-amido sulfones **1a** or **1e** (1.0 eq., 0.08 mmol), K_2CO_3 (55 mg, 5.0 eq., 0.4 mmol) and nitromethane (21 μL, 5.0 eq., 0.4 mmol) were stirred at room temperature in toluene (0.4 mL) until starting material was all converted in the intermediate. Then, the reaction mixture was allowed to slowly warm up to room temperature and the stirring was continued for 72 h. Purification by flash chromatography on silica gel (Hexane/Ethyl acetate, 5:1) afforded the racemic products **3a** (10.5 mg, 45%) and **3e** (11.8 mg, 40%).

Asymmetric synthesis of *tert*-butyl 1-(1-nitroethyl)-3-oxoisoindoline-2-carboxylate. (4)

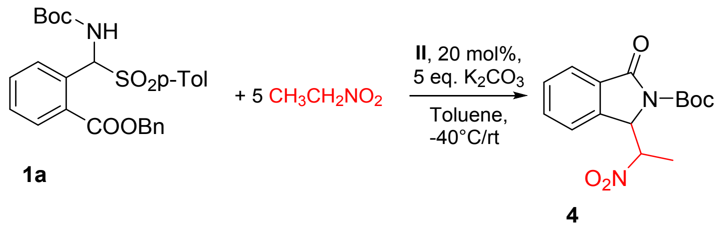

In an ACE tube, α-amido sulfone **1a** (27 mg, 1.0 eq., 0.08 mmol), K_2CO_3 (55 mg, 5.0 eq., 0.4 mmol), nitroethane (28 μL, 5.0 eq., 0.4 mmol) and organocatalyst **II** (20 mol%) were stirred in at -40° in toluene (0.4 mL) until starting material was all converted in the intermediate. Then, the reaction mixture was allowed to slowly warm up to room temperature and the stirring was continued until the intermediate disappeared (96 h). Then, the mixture was directly purified by flash chromatography on silica gel (Hexane/Ethyl acetate, 7:3). Yield= 14 mg, 54%. Characterization of compounds is reported in the main text. Copies of the spectra are reported below.

Racemic procedure for the synthesis of compound 4.

α-amido sulfones **1a** (27 mg, 1.0 eq., 0.08 mmol), K_2CO_3 (55 mg, 5.0 eq., 0.4 mmol) and nitroethane (28 μL, 5.0 eq., 0.4 mmol) were stirred at room temperature in toluene (0.4 mL) until starting material was all converted in the intermediate. Then, the reaction mixture was allowed to slowly warm up to room temperature and the stirring was continued until the intermediate disappeared (110 h). Purification by flash chromatography on silica gel (Hexane/Ethyl acetate, 7:3) gave the racemic product. Yield= 8 mg, 33%.

Crystallographic data

Crystals of compound **3e** suitable for single crystal X-ray diffraction analysis were obtained dissolving 14 mg of the compound in a mixture of chloroform (0.3 mL) and hexane (0.1 mL) at room temperature. After the complete dissolution, the saturated solution was cooled down to -18°C. Crystals were obtained after few days. For the measurement, a colourless prismatic crystal of 0.34 mm x 0.21 mm x 0.07 mm was selected and mounted on a cryoloop with paratone oil.

Data collection was performed at room temperature with a Bruker D8 QUEST diffractometer equipped with a PHOTON II detector using Cu*Kα* radiation (λ= 1.54178 Å).

Data indexing, integration and reduction were performed using *CrysAlisPro* ver. 1.171.41.122a.[9] Empirical absorption correction was performed with *CrysAlisPro* ver. 1.171.41.122a using spherical harmonics, implemented in SCALE3 ABSPACK scaling algorithm. The structure was solved using SHELXS-97[10] and refined through full matrix least-squares based on *F*[10] using the program SHELXL.[11]

Non-hydrogen atoms were refined anisotropically, hydrogen atoms were positioned geometrically and included in structure factors calculations but not refined.

The chirality on carbon atom C2 (R) was successfully assigned by anomalous-dispersion effects in diffraction measurements on the crystal (Flack parameter -0.025(14)).

ORTEP diagrams (Figure S1) was drawn using *OLEX2*.[12] In Table S1 are reported the crystallographic data.

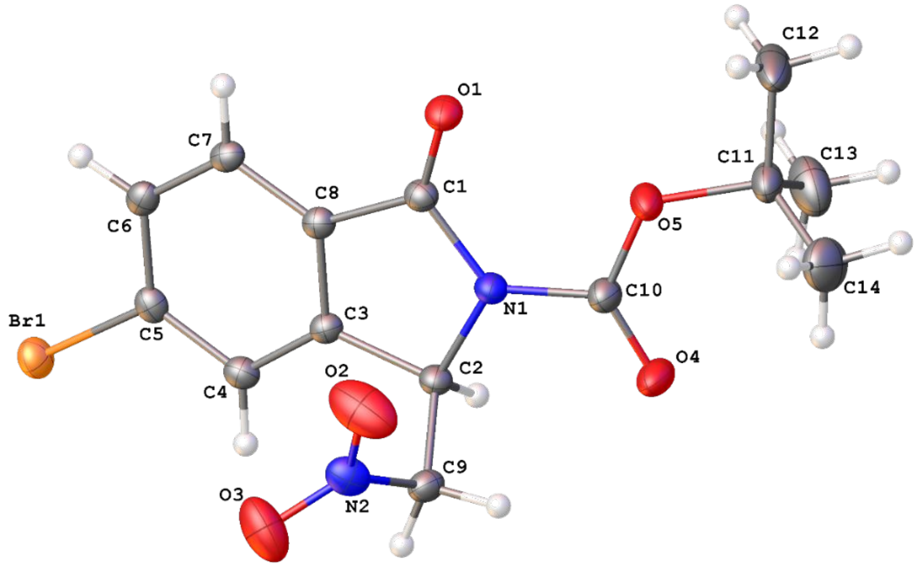

Figure S1. ORTEP drawings for compound **3e**. Atom types: C grey, H white, Br orange, O red, N blue. Ellipsoids are drawn at 20% probability level. CCDC 2143635 contains the supplementary crystallographic data, which can be accessed available free of charge from The Cambridge Crystallographic Data Centre via www.ccdc.cam.ac.uk/structures.

Table S1. Crystallographic data for compound **3e**.

	**3e**
**T (K)**	296
**Formula**	$C_{14}H_{15}BrN_2O_5$
**Formula weight**	371.18
**System**	Orthorhombic
**Space group**	$P2_12_12_1$
a **(Å)**	6.50468(9)
b **(Å)**	7.19264(8)
c **(Å)**	33.7877(4)
α **(°)**	90
β **(°)**	90
γ **(°)**	90
V **(Å^3)**	1580.79(3)
**Z**	4
D_x **(g cm^{-3})**	1.560
**λ (Å)**	1.54178
μ **(mm^{-1})**	3.779
F_{000}	752.0
**R1 (I > 2σI)**	0.0379(2719)
$_wR_2$	0.1024(2861)
**N. of param.**	203
**GooF**	1.069
**Flack parameter**	-0.025(14)
ρ_{min}, ρ_{max} **(eÅ^3)**	-0.51, 0.40

REFERENCES

[1]Santoso, K. T.; Cheung, C. Y.; Hards, K.; Cook, G. M.; Stocker, B. L.; Timmer. M. S. M. Synthesis and Investigation of Phthalazinones as Antitubercular Agents. *Chem Asian J.* **2019,** 14, 1278-1285.

[2]Du, J.; Chen, J.; Xia, H; Zhao, Y.; Wang, F.; Liu, H.; Zhou, W.; Wang, B. Commercially Available CuO Catalyzed Hydrogenation of Nitroarenes Using Ammonia Borane as a Hydrogen Source. *Chem. Cat. Chem.* **2020,** *12*, 2426-2430.

[3] (a) Beck, D. E.; Abdelmalak, M.; Wei, L.; P. V. Narasimha Reddy; Tender, G. S.; O'Neill, E.; Agama, K.; Marchand, C.; Pommier, Y.; Cushman, M. Discovery of Potent Indenoisoquinoline Topoisomerase I Poisons Lacking the 3-Nitro Toxicophore. *J. Med. Chem.* **2015***, 58,* 3997-4015. (b) Jiao, J.; Shafer, J. Isoindolin-1-ones as macrophage migration inhibitory factor (MIF) inhibitors. US **2015**/0175540.

[4]Vila, N.; Besada, P.; Viña, D.; Sturlese, M.; Moro, S.; Terán, C. Synthesis, biological evaluation and molecular modeling studies of phthalazin-1(2H)-one derivatives as novel cholinesterase inhibitors. *RSC Adv.* **2016,** *6*, 46170-46185**.**

[5] (a) Wu, S.; Liu, N.; Dong, G.; Ma, L.; Wang, S.; Shi, W.; Fang, K.; Chen, S.; Li, J.; Zhang, W.; Sheng, C.; Wang, W. Facile construction of pyrrolo[1,2-b]isoquinolin-10(5H)-ones via a redox-amination–aromatization–Friedel–Crafts acylation cascade reaction and discovery of novel topoisomerase inhibitors. *Chem. Commun.* **2016**, *52*, 9593-9596. (b) Bolliger, J.; Frech, C. The 1,3-Diaminobenzene-Derived Aminophosphine Palladium Pincer Complex {$C_6H_3[NHP(piperidinyl)_2]_2Pd(Cl)$} – A Highly Active Suzuki–Miyaura Catalyst with Excellent Functional Group Tolerance. *Adv. Synth. Catal.* **2010**, 352-1075-1080.

[6] Zhang, Y.; Ao, Y. F.; Huang, Z. T.; Wang, De X.; Wang, M. X.; Zhu, J. Chiral Phosphoric Acid Catalyzed Asymmetric Ugi Reaction by Dynamic Kinetic Resolution of the Primary Multicomponent. *Angew. Chem. Int. Ed.* **2016**, *55*, 5282-5285.

[7] (a) Sharique, M.; Tamba, U. K. *N*-Heterocyclic carbene based catalytic platform for Hauser–Kraus annulations *Chem. Sci.* **2020**,11, 7239-7243. (b) Longhui, Y.; Jun L.; Hongyu W.; Lijun, X.; Wu, Y.; a Zheng, C.; Gang Z. Asymmetric Dieckmann Condensation towards Spirocyclic Oxindoles Catalyzed by Amino Acid-derived Phosphonium Salts. *Adv. Synth. Catal.* **2022**, 302-306. (c) Mirabdolbaghi, R.; Dudding, T. An Indium-Mediated Allylative/Transesterification DFT-Directed Approach to Chiral C(3)-Functionalized Phthalides *Org. Lett.* **2012**, *14*, 3748-3751.

[8] Tillman, A. L.; Ye, J.; Dixon. D. J. Direct enantio- and diastereoselective Mannich reactions of malonate and β-keto esters with N-Boc and N-Cbz aldimines catalysed by a bifunctional cinchonine derivative. *Chem. Commun.* **2006**, 1191-1193.

[9] Rigaku OD (2021). *CrysAlis PRO*, Version 1.171.41.122a. Rigaku Oxford Diffraction.

[10] Sheldrick, G. M. A short history of SHELX. *Acta Cryst. A64*, **2008**, 112–122.

[11] Sheldrick, G. M. Crystal structure refinement with SHELXL. *Acta Cryst. C71*, **2015**, 3–8.

[12] Dolomanov, O. V. ; Bourhis, L. J.; Gildea, R. J.; Howard, J. A. K.; Puschmann, H. OLEX2: a complete structure solution, refinement and analysis program *J. Appl. Cryst.* **2009**, 42, 339–341.

Copies of ^{1}H NMR and ^{13}C NMR Spectra of intermediates and starting materials.

^{1}H NMR (400 MHz, $CDCl_3$)

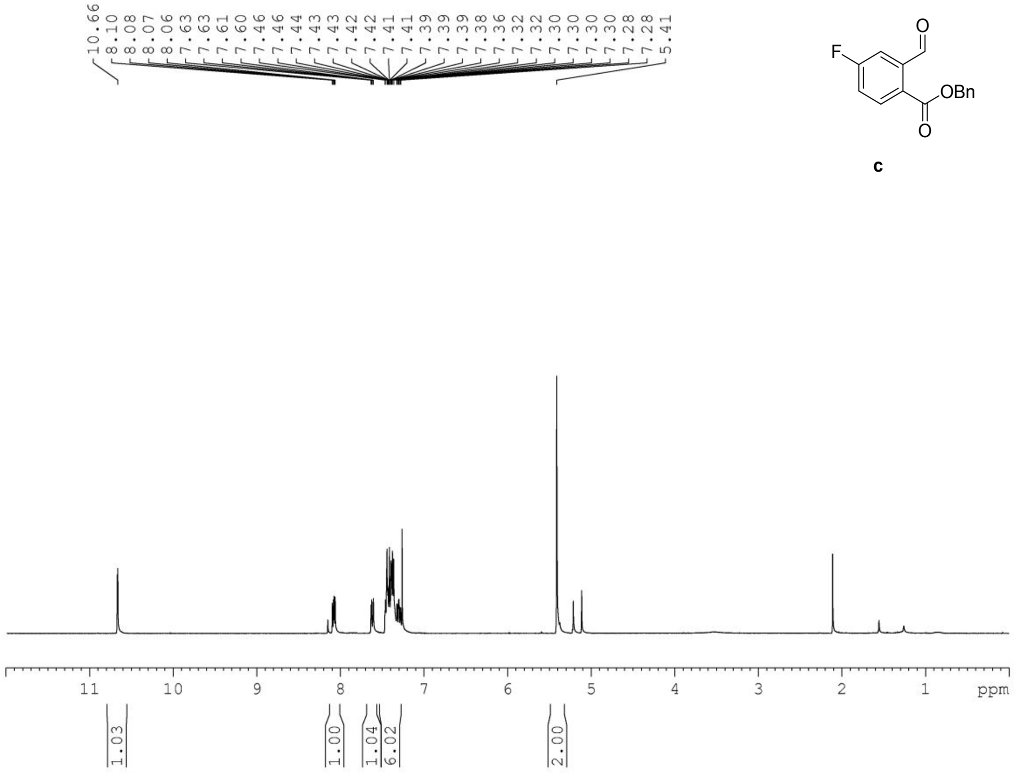

c

^{13}C{^{1}H} NMR (75 MHz, $CDCl_3$)

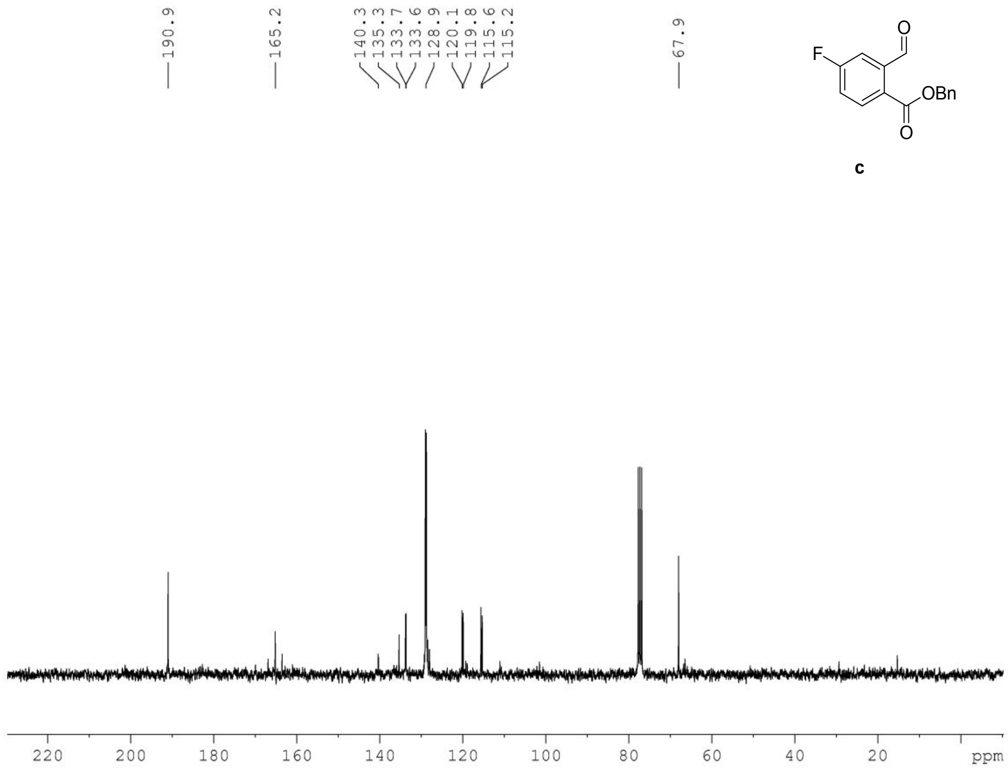

c

^{1}H NMR (300 MHz, $CDCl_3$)

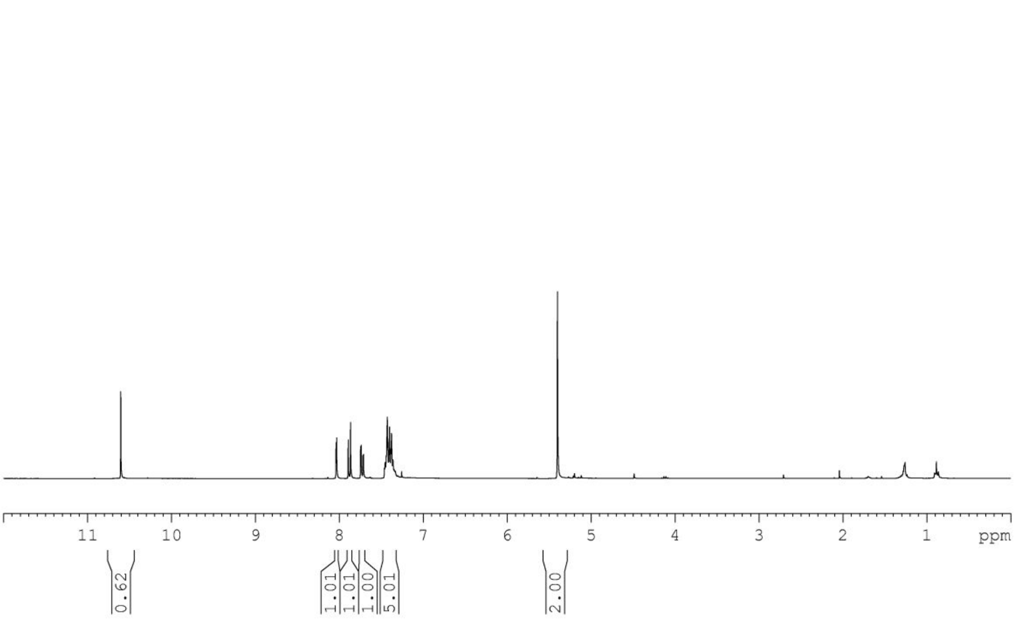

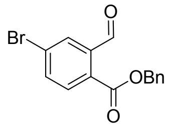

e

^{13}C{^{1}H} NMR (75 MHz, $CDCl_3$)

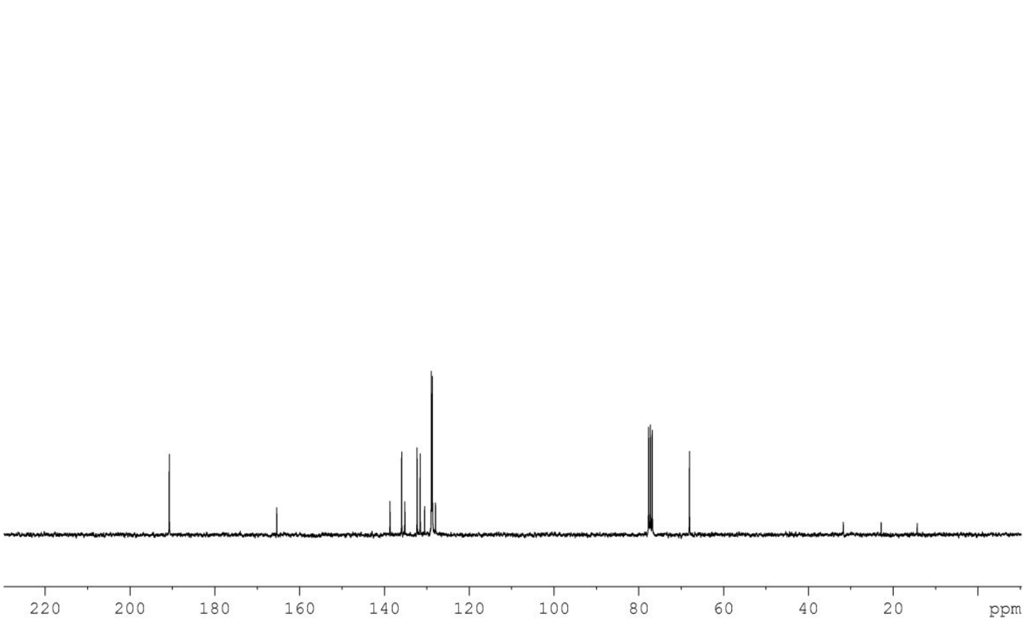

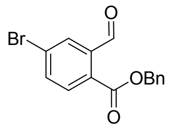

e

^1H NMR (300 MHz, $CDCl_3$)

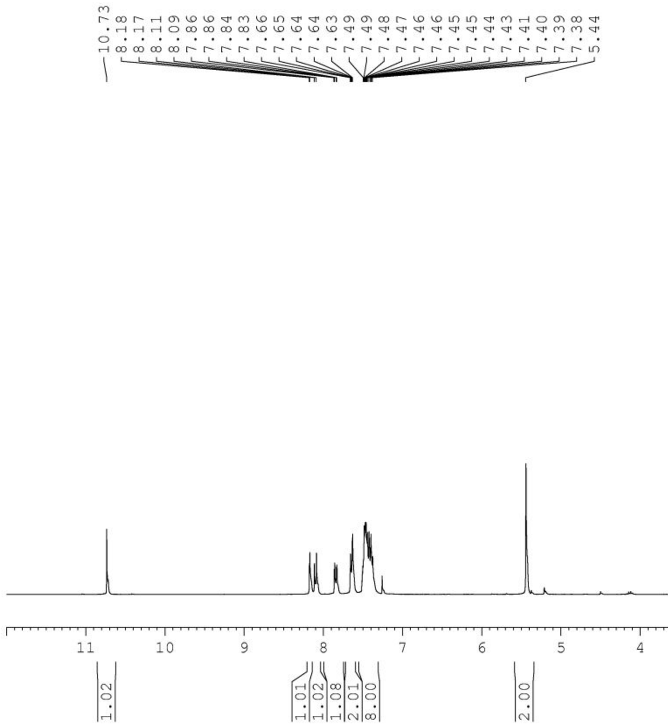

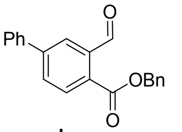

h

$^{13}C\{^1H\}$ NMR (75 MHz, $CDCl_3$)

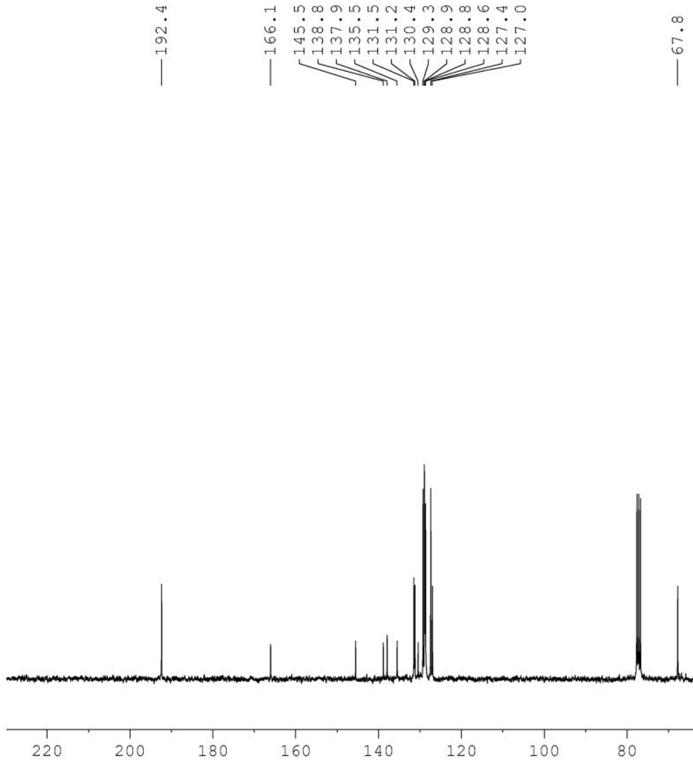

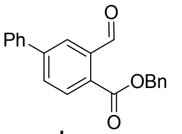

h

^{1}H NMR (400 MHz, $CDCl_3$)

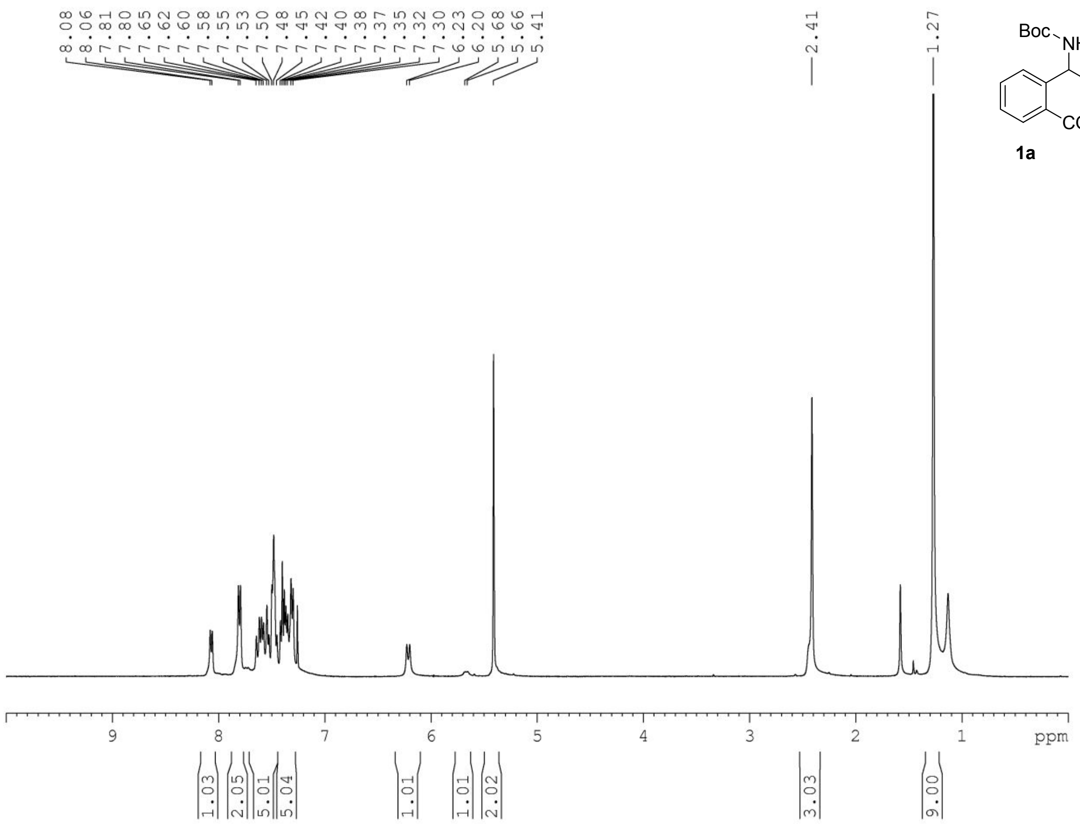

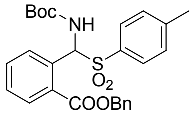

1a

$^{13}C\{^{1}H\}$ NMR (100 MHz, $CDCl_3$)

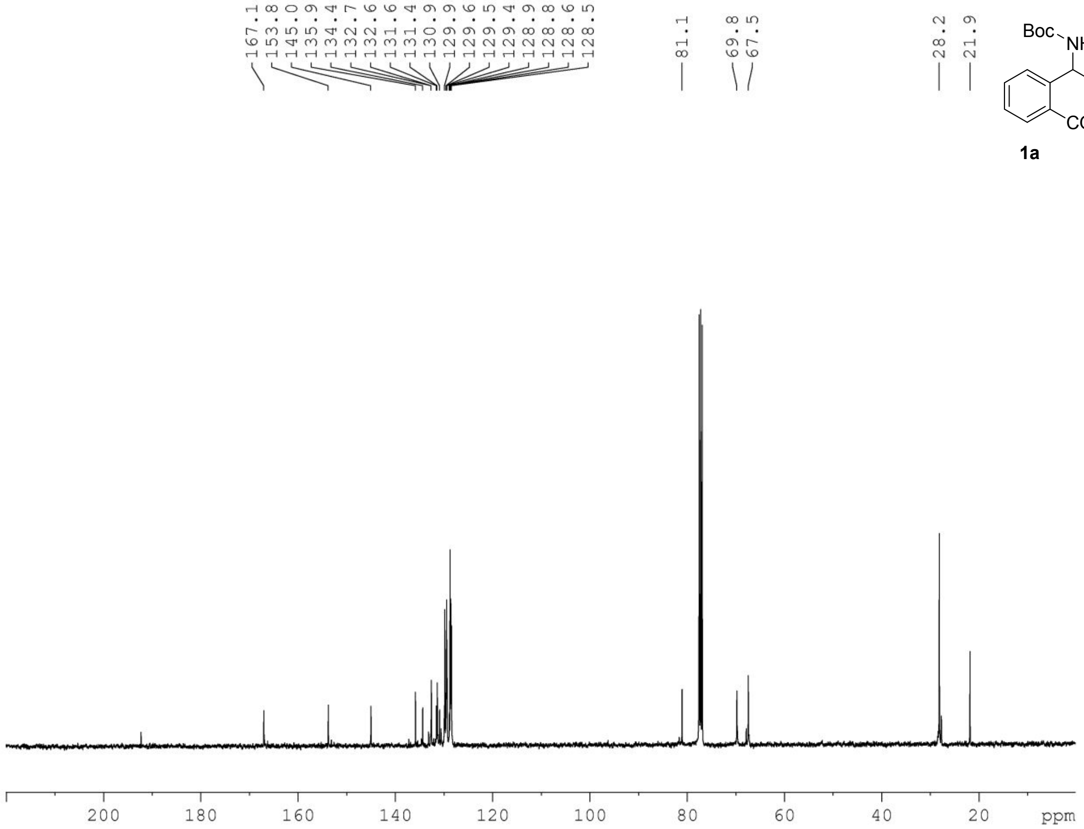

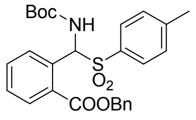

1a

^1H NMR (300 MHz, $CDCl_3$)

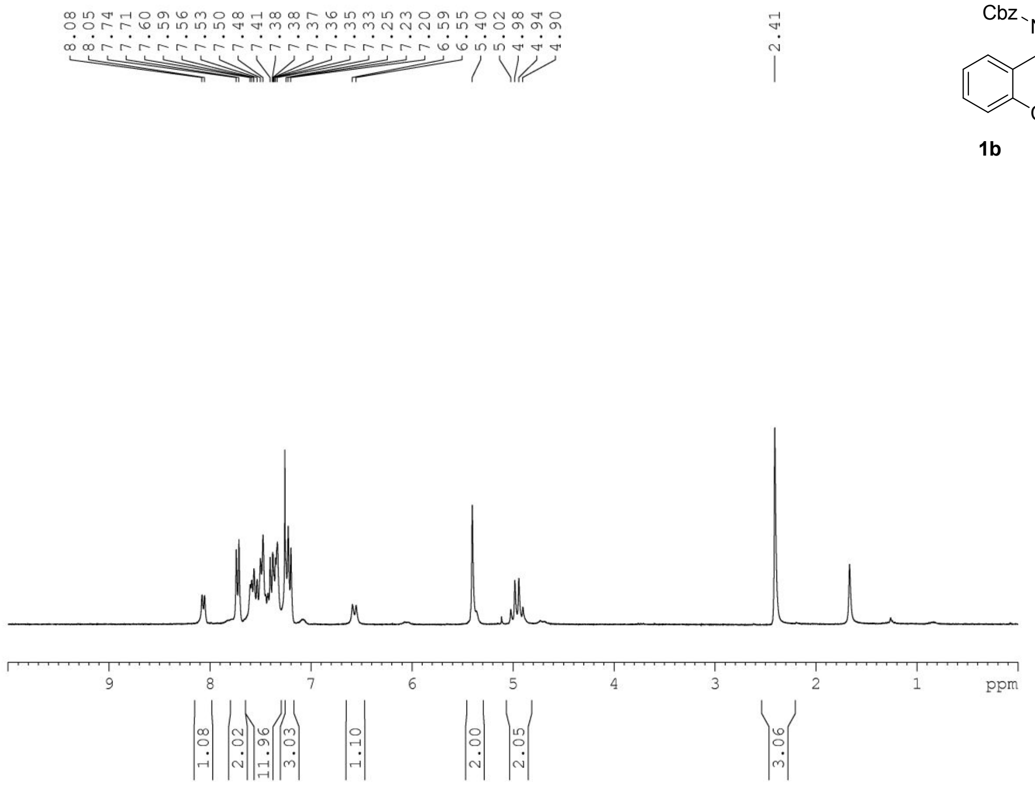

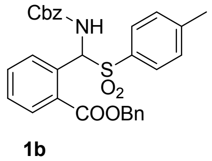

1b

$^{13}C\{^1H\}$ NMR (75 MHz, $CDCl_3$)

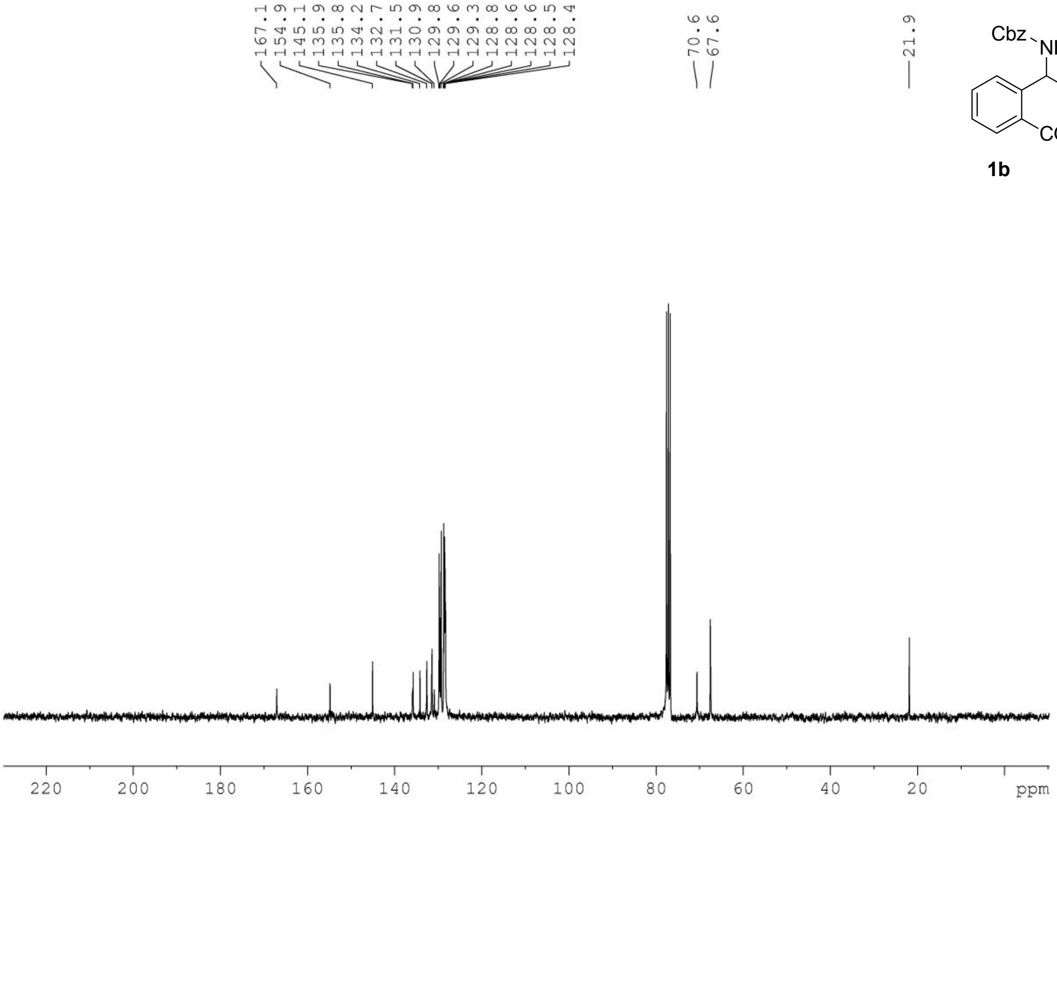

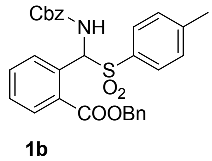

1b

^{1}H NMR (400 MHz, $CDCl_3$)

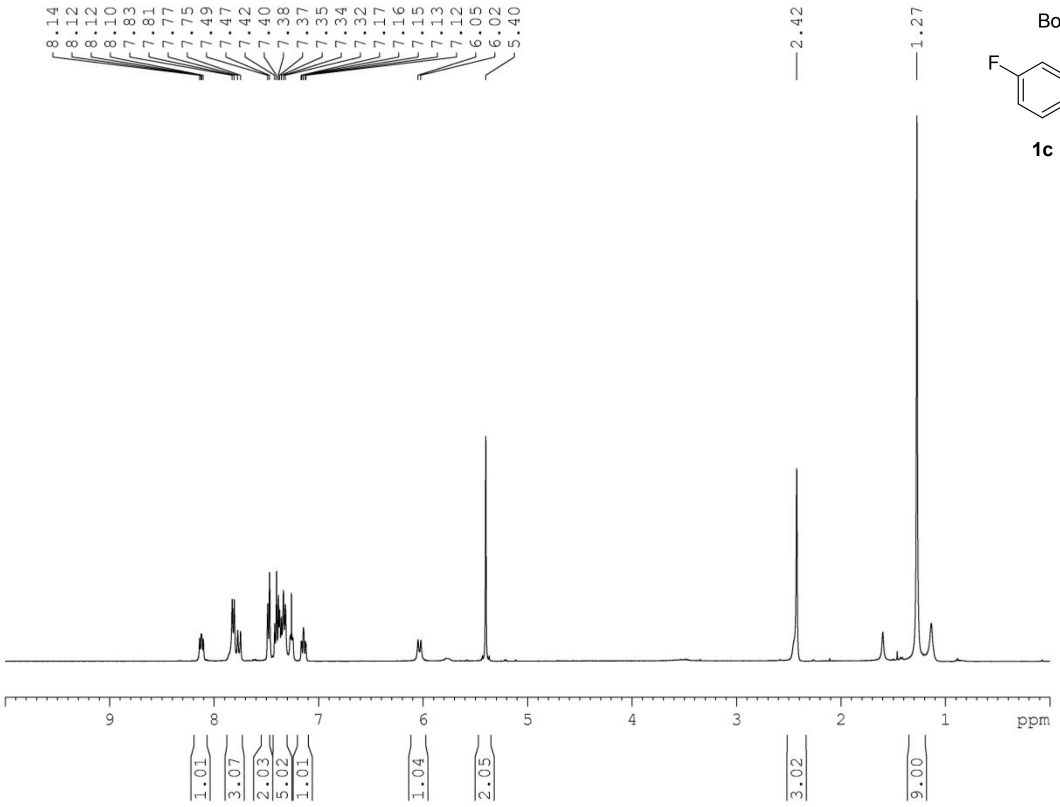

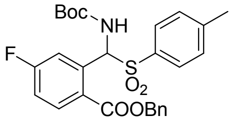

1c

^{13}C{^{1}H} NMR (100 MHz, $CDCl_3$)

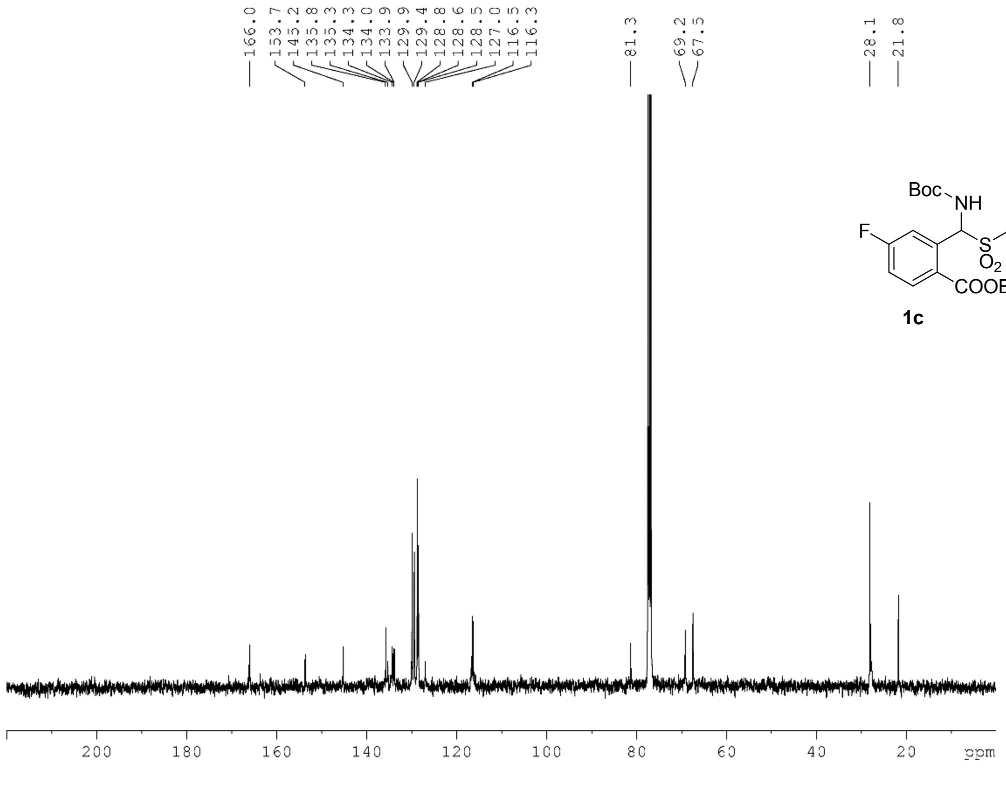

1c

^{1}H NMR (300 MHz, $CDCl_3$)

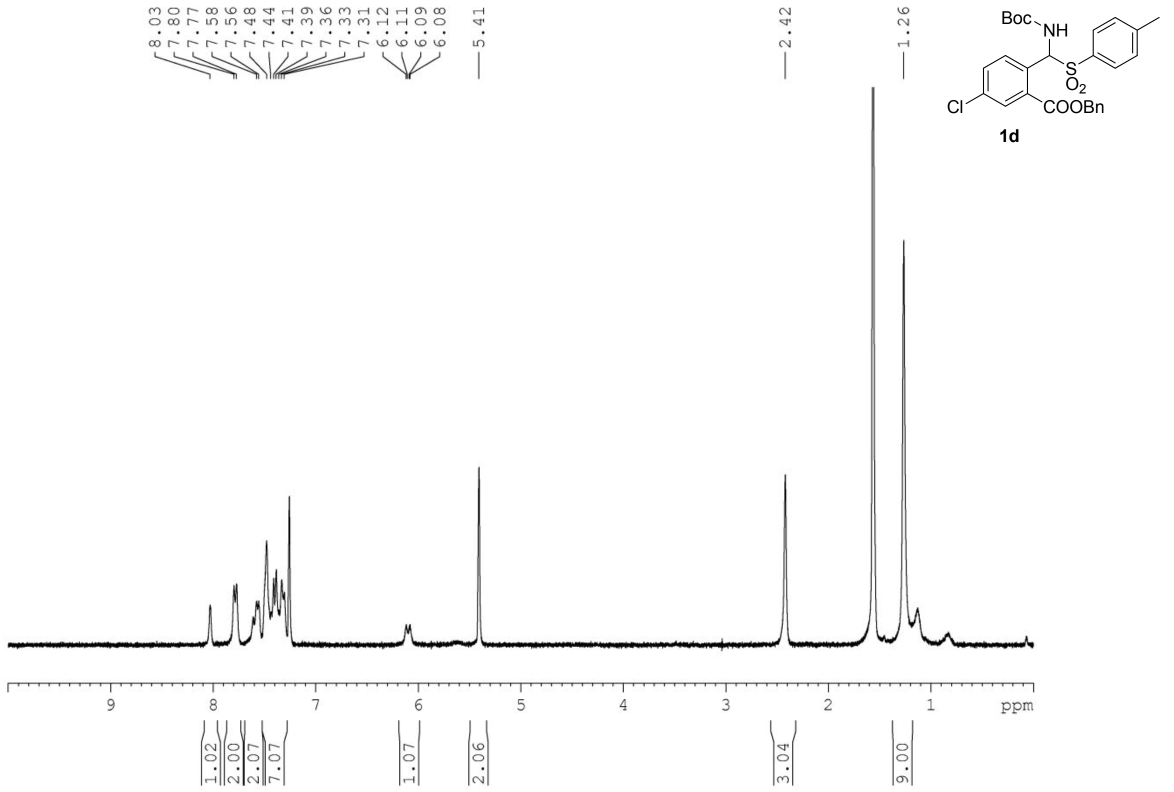

$^{13}C\{^{1}H\}$ NMR (100 MHz, $CDCl_3$)

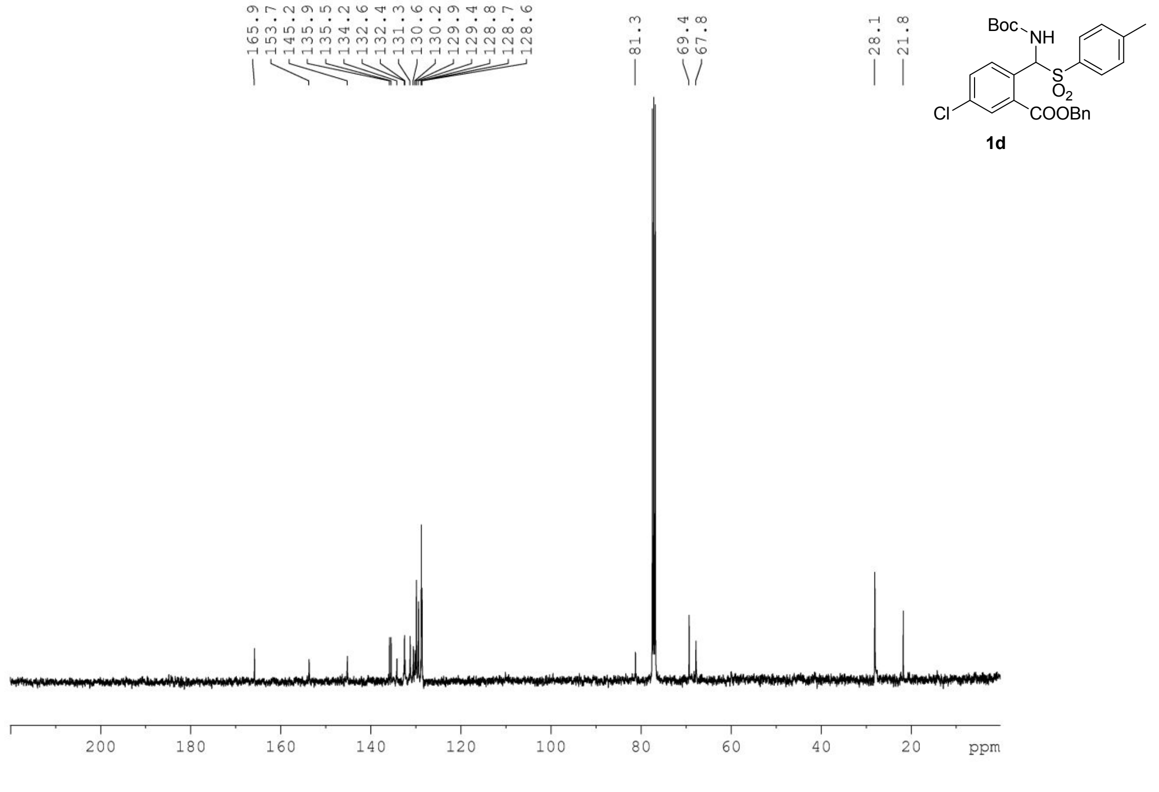

^{1}H NMR (400 MHz, $CDCl_3$)

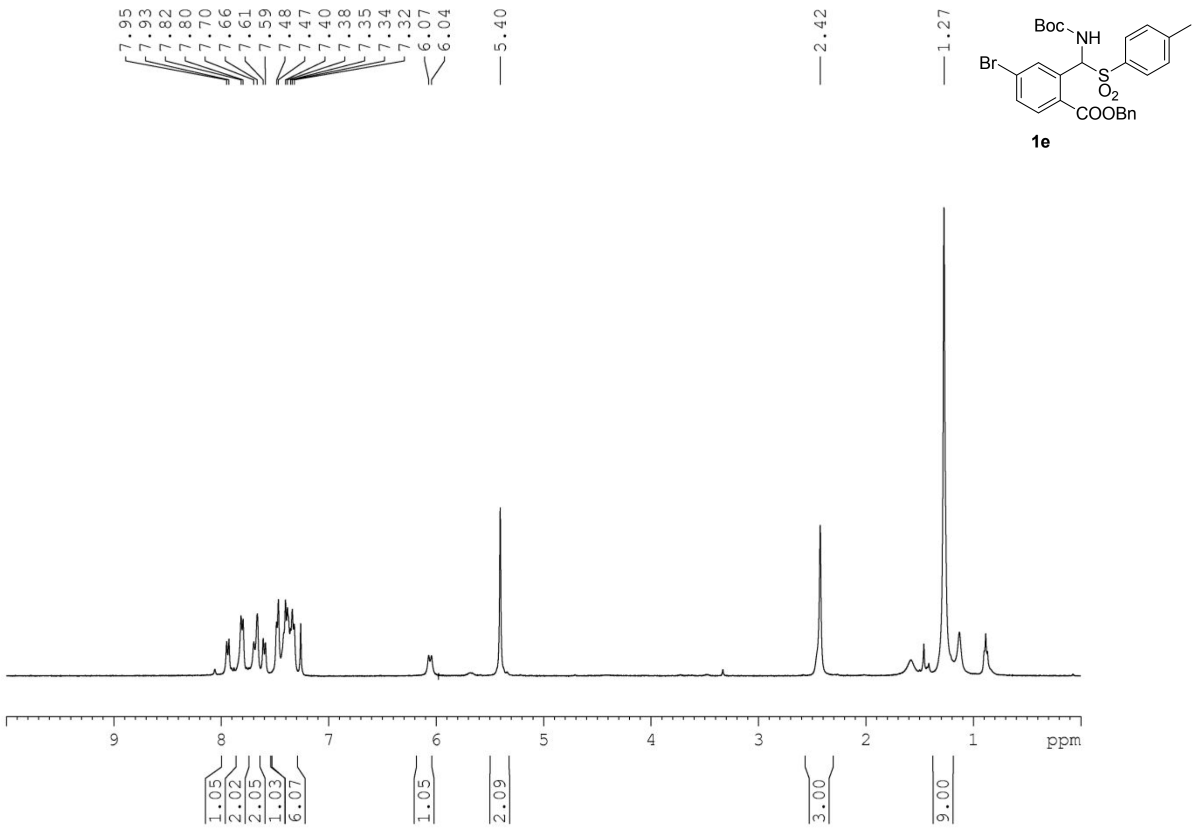

1e

^{13}C{^{1}H} NMR (75 MHz, $CDCl_3$)

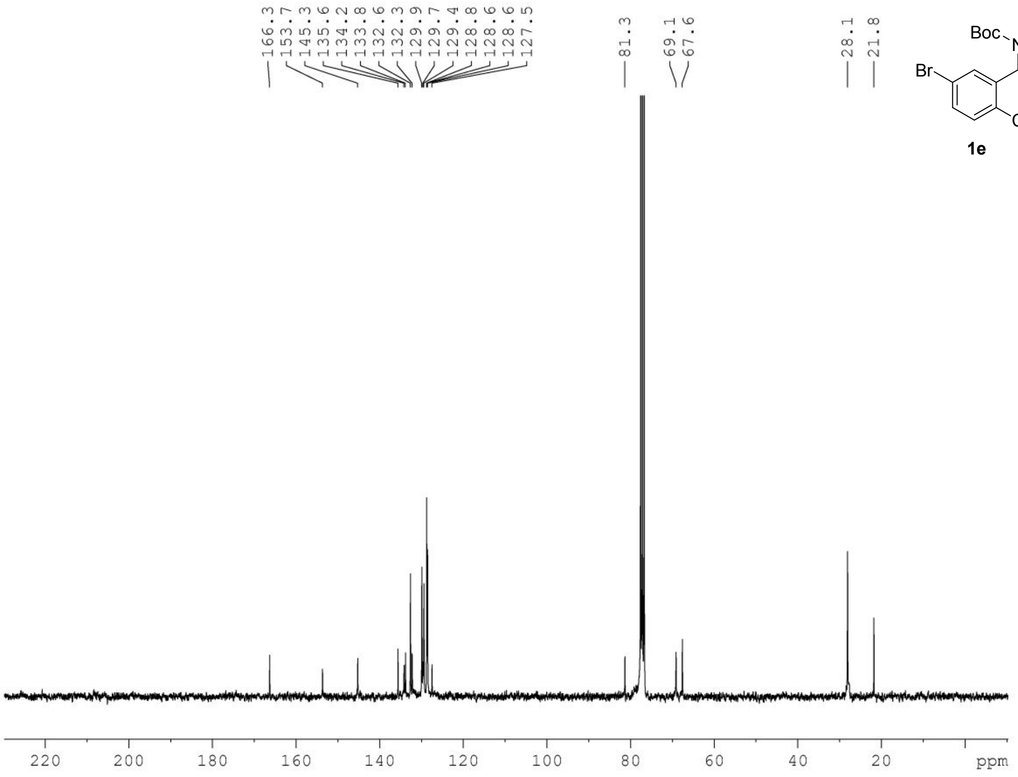

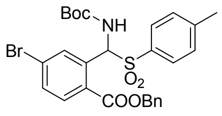

1e

^{1}H NMR (300 MHz, $CDCl_3$)

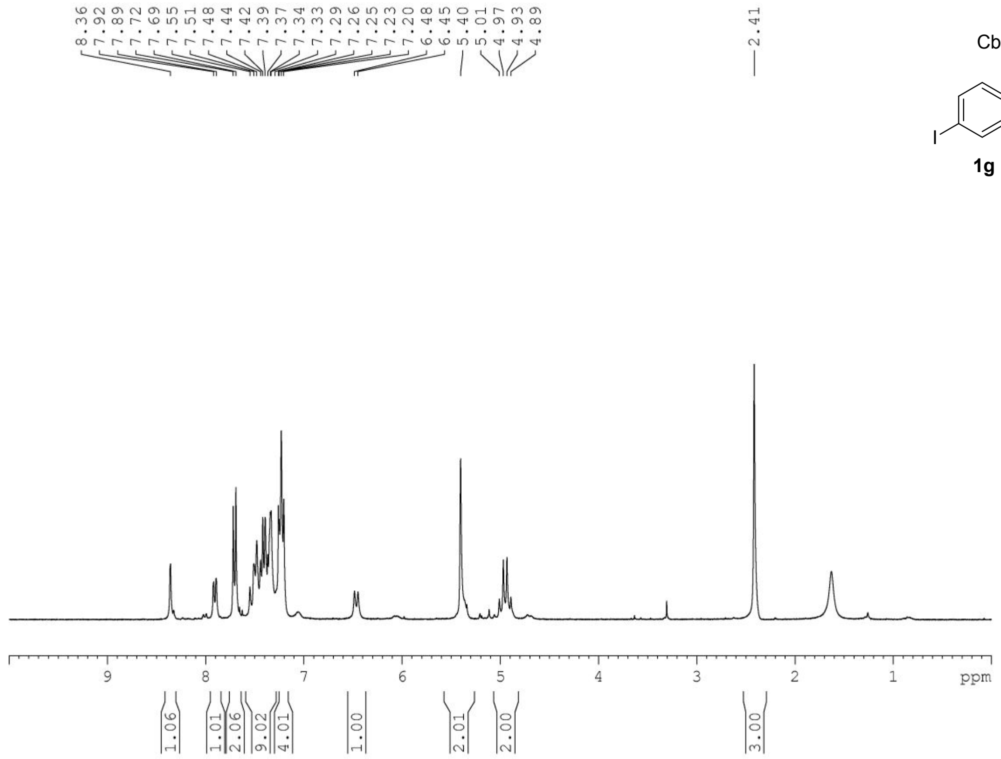

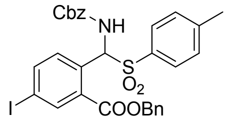

1g

$^{13}C\{^{1}H\}$ NMR (100 MHz, $CDCl_3$)

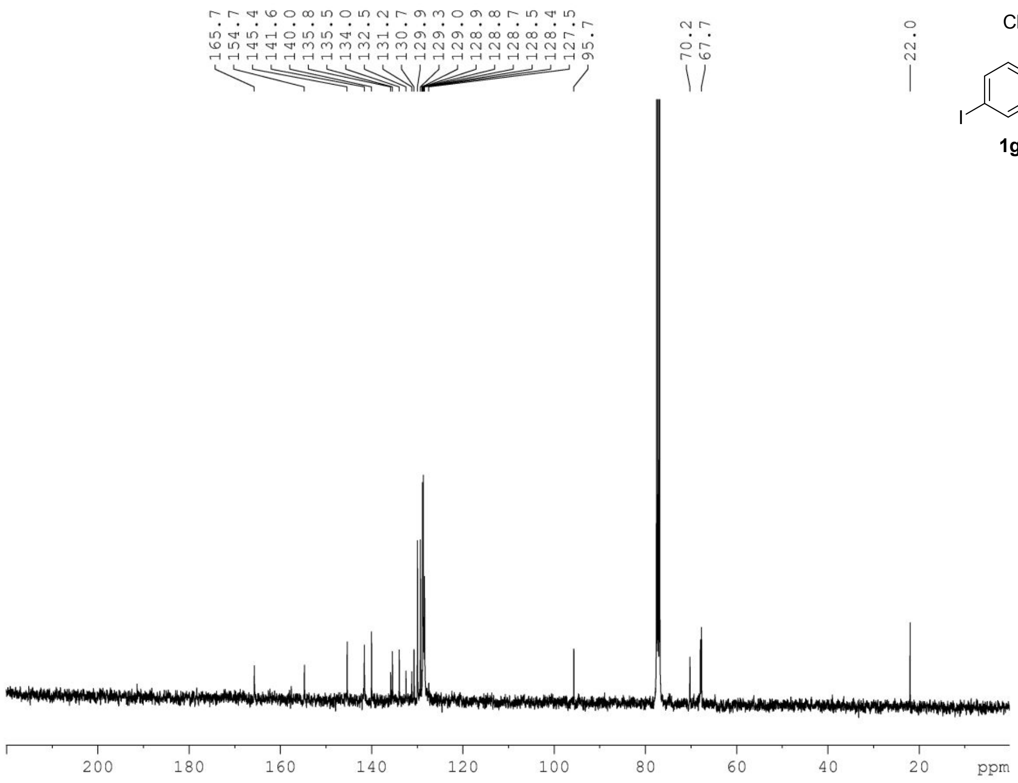

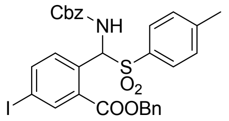

1g

^1H NMR (400 MHz, $CDCl_3$)

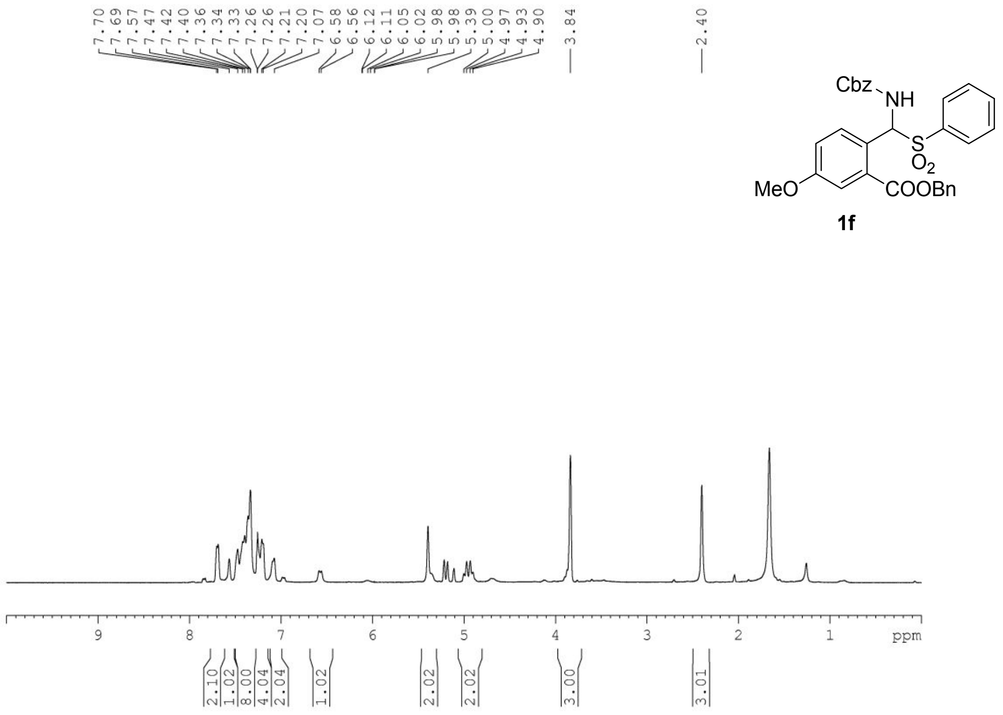

1f

^1H NMR (300 MHz, $CDCl_3$)

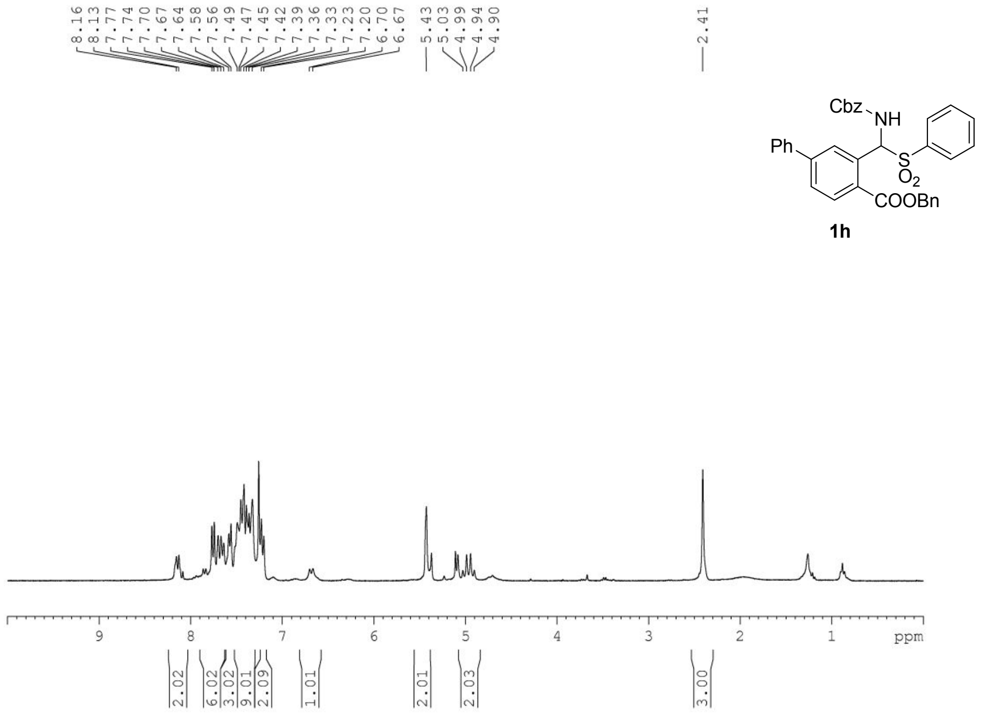

1h

^{1}H NMR (400 MHz, $CDCl_3$)

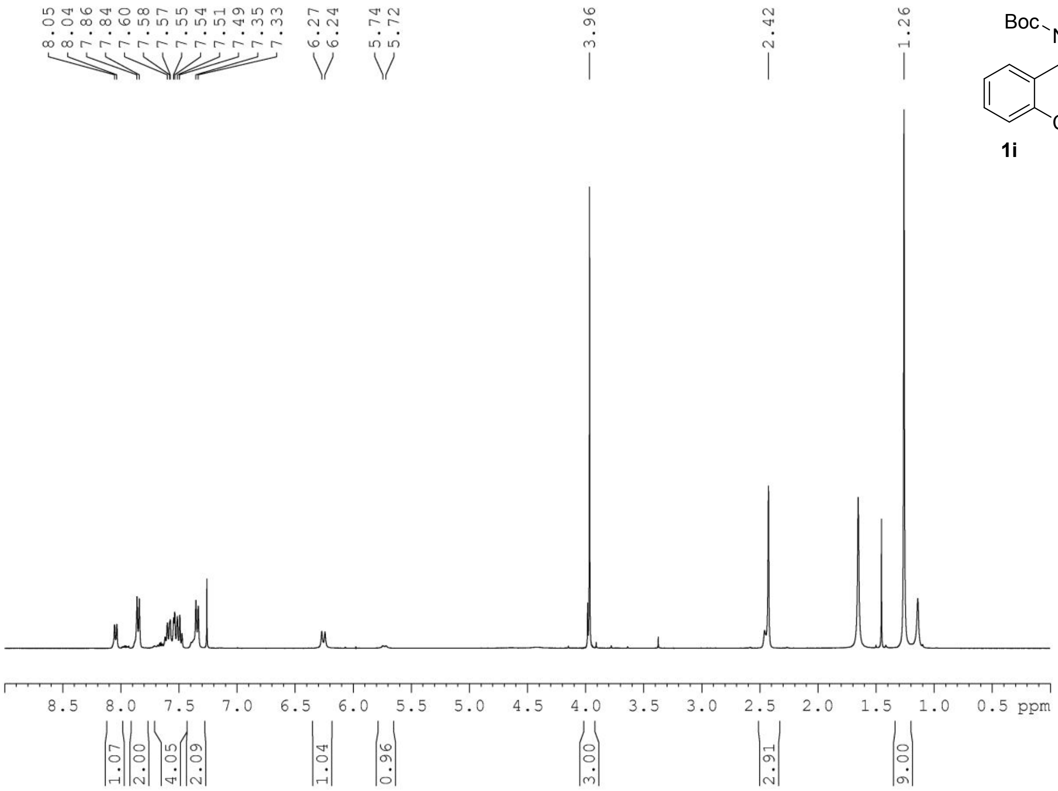

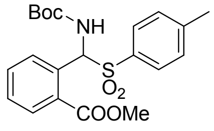

1i

^{13}C{^{1}H} NMR (75 MHz, $CDCl_3$)

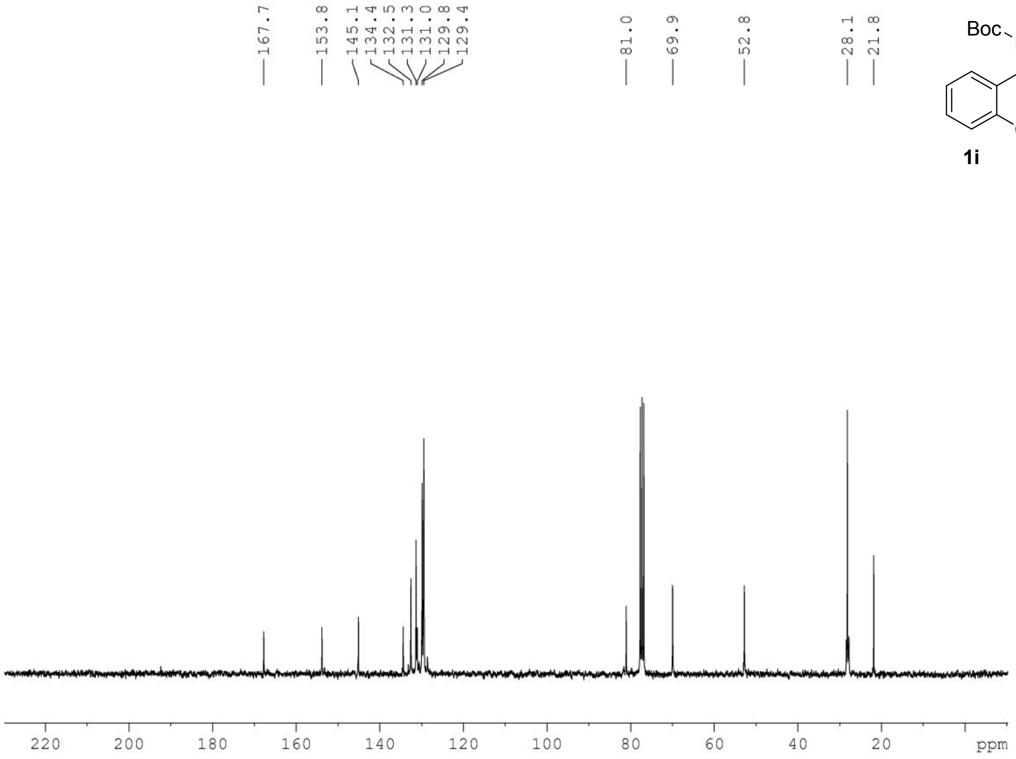

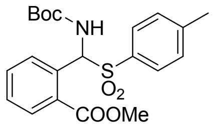

1i

^{1}H NMR (300 MHz, $CDCl_3$)

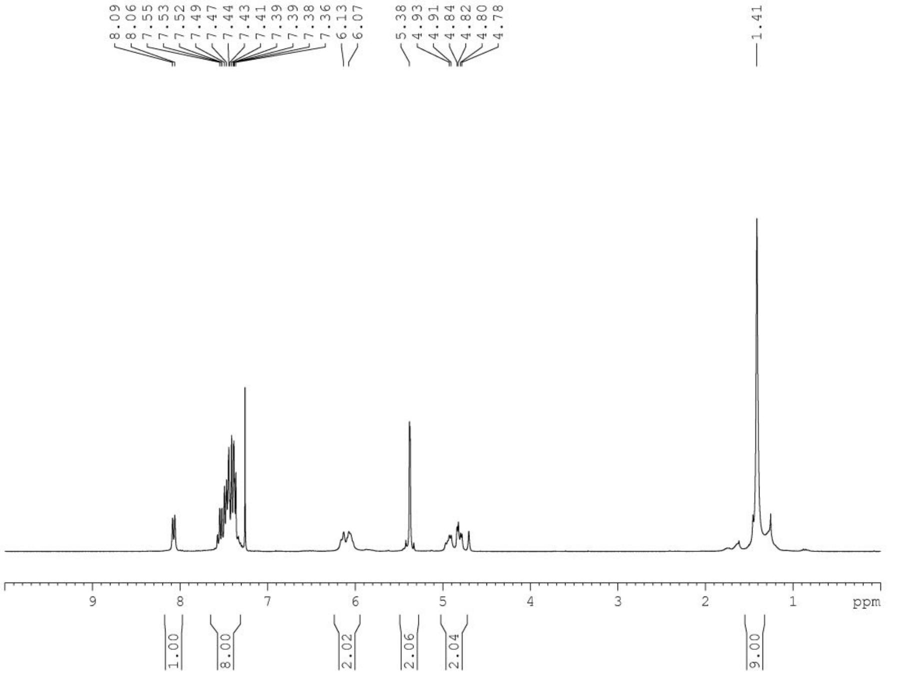

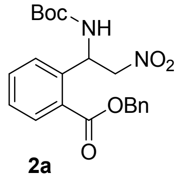

2a

$^{13}C\{^{1}H\}$ NMR (75 MHz, $CDCl_3$)

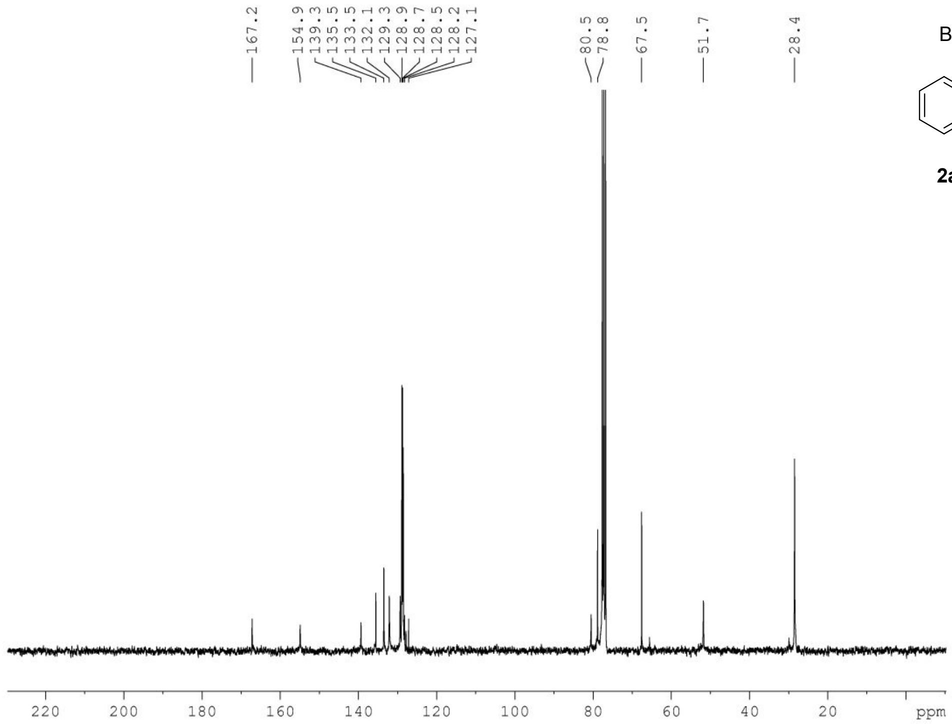

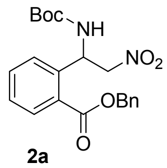

2a

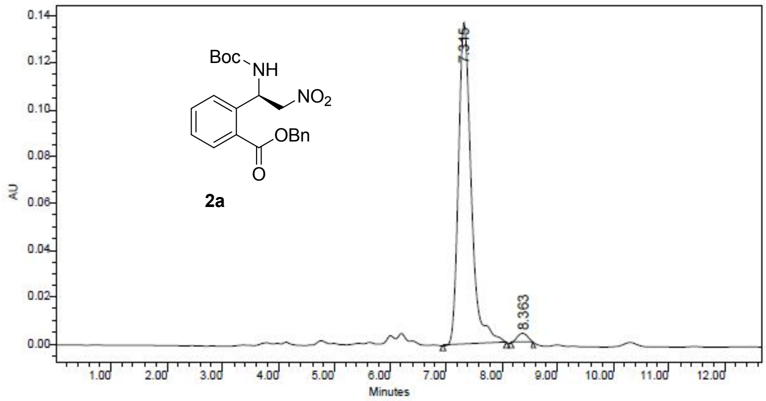

	RT (min)	Area (□V*sec)	% Area	Height (□V)	% Height
1	7.315	2195737	97.61	137177	97.15
2	8.363	53678	2.39	4018	2.85

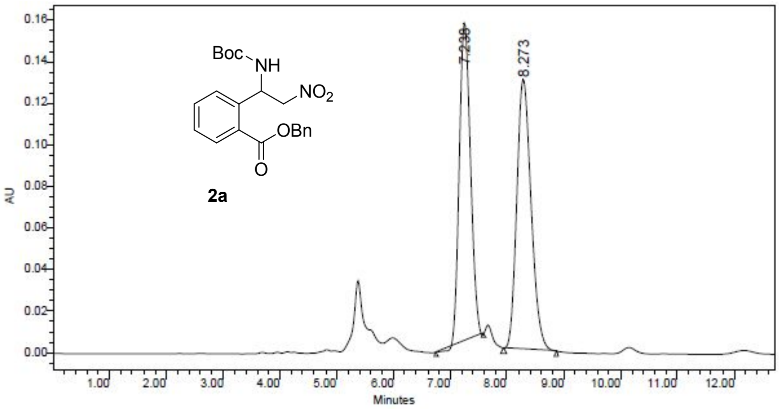

	RT (min)	Area (□V*sec)	% Area	Height (□V)	% Height
1	7.238	2107575	48.03	153210	54.08
2	8.273	2280811	51.97	130102	45.92

Copies of ^{1}H NMR, ^{13}CNMR spectra and HPLC traces of enantioenriched isoindolin-1-ones 3a-3h

^{1}H NMR (300 MHz, $CDCl_3$)

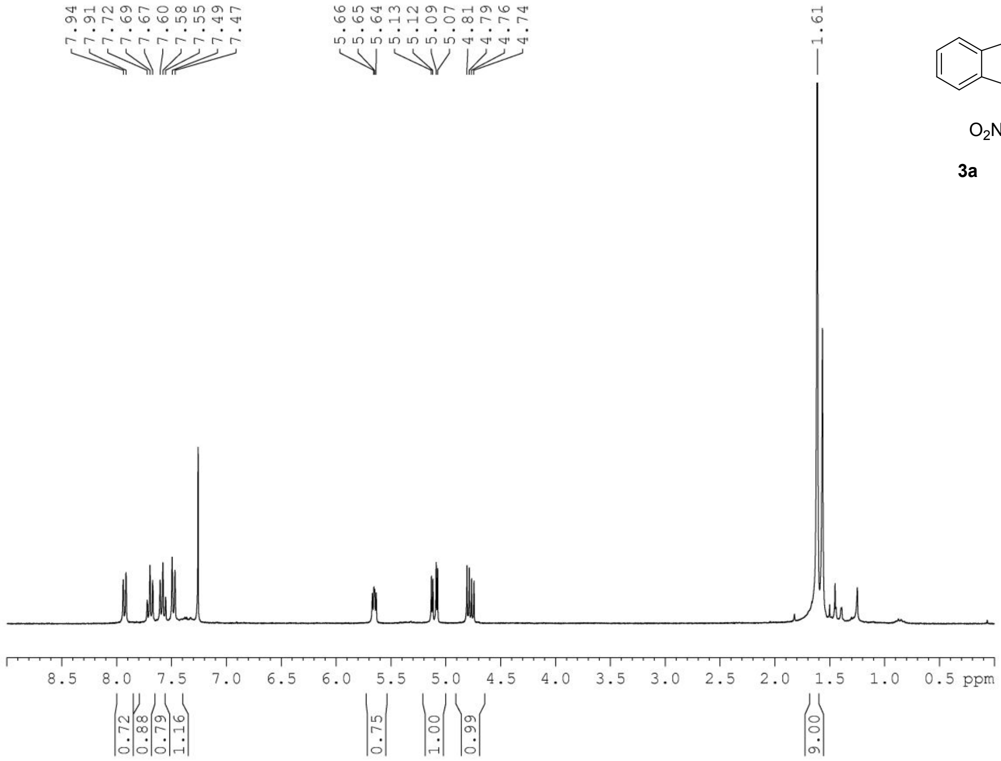

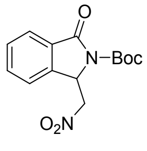

3a

$^{13}C\{^{1}H\}$ NMR (100 MHz, $CDCl_3$)

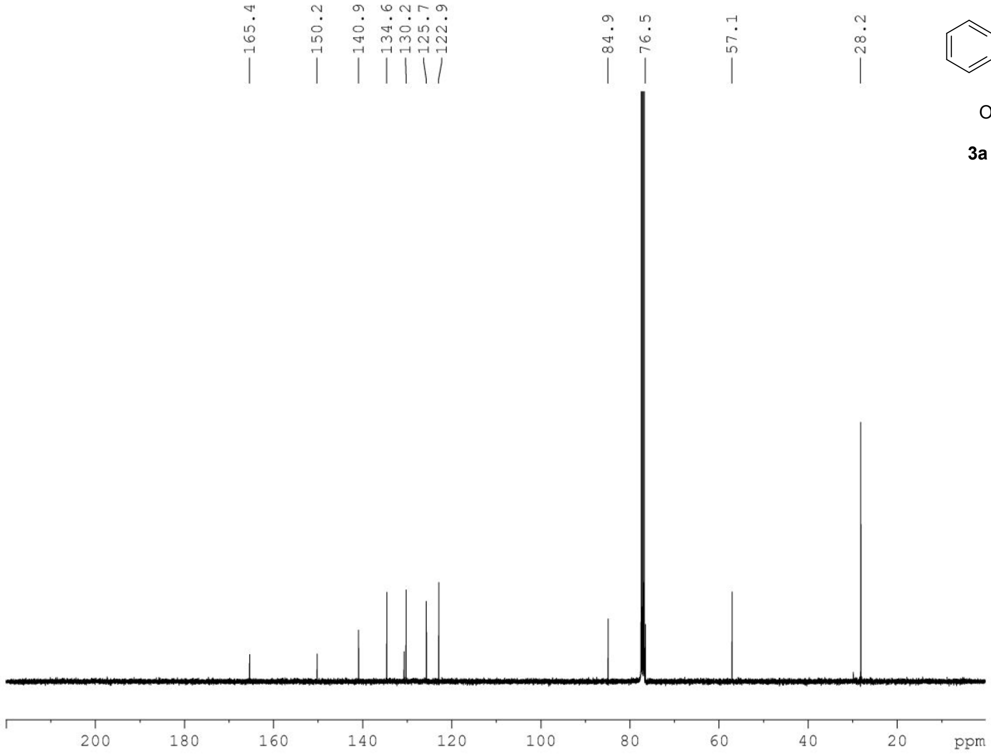

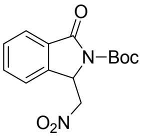

3a

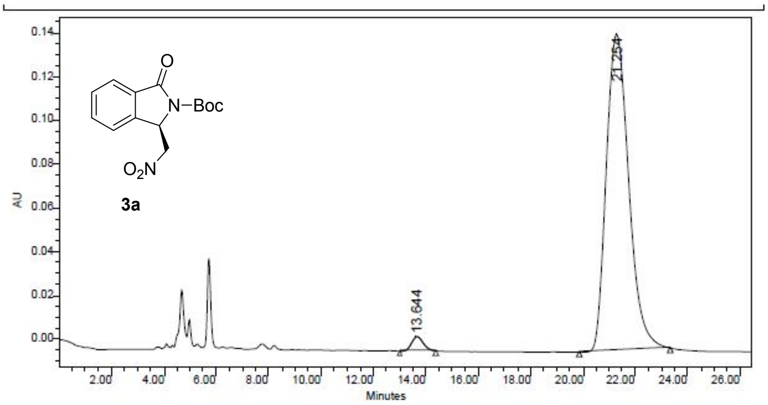

	RT (min)	Area (□V*sec)	% Area	Height (□V)	% Height
1	13.644	203486	2.30	6562	4.34
2	21.254	8627535	97.70	144591	95.66

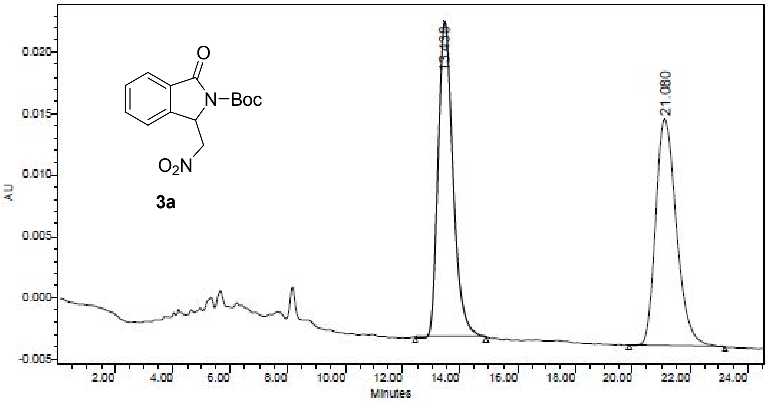

	RT (min)	Area (□V*sec)	% Area	Height (□V)	% Height
1	13.438	930526	50.04	25707	58.22
2	21.080	928980	49.96	18447	41.78

^{1}H NMR (400 MHz, $CDCl_3$)

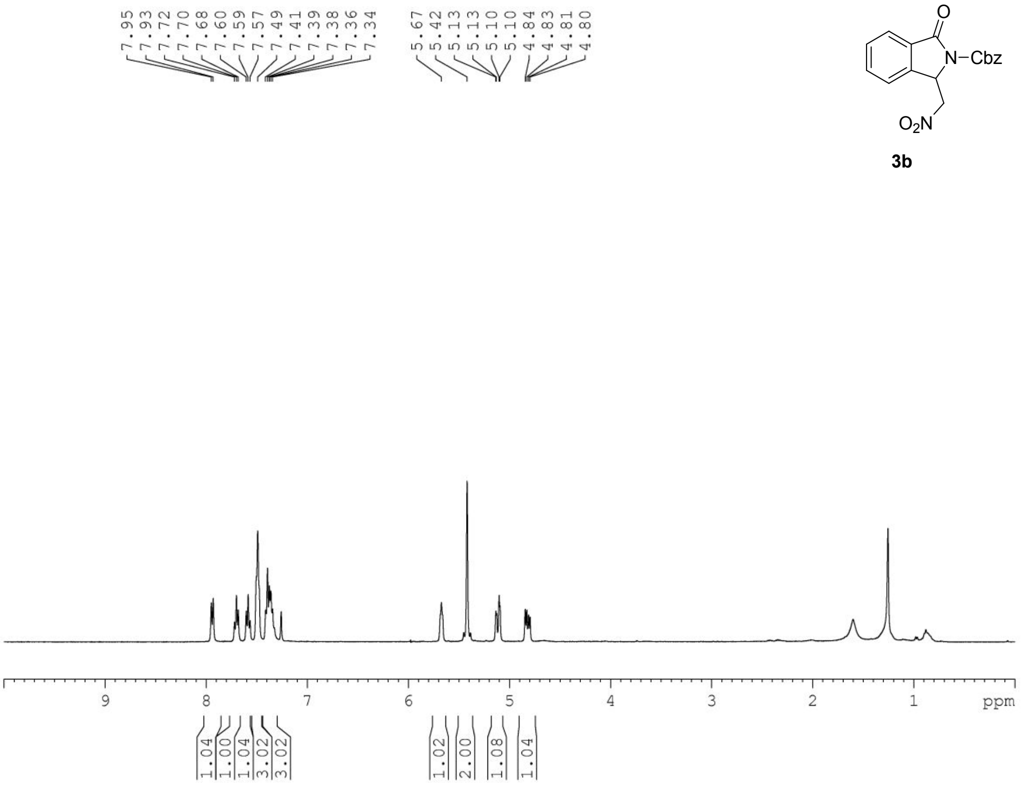

3b

$^{13}C\{^{1}H\}$ NMR (100 MHz, $CDCl_3$)

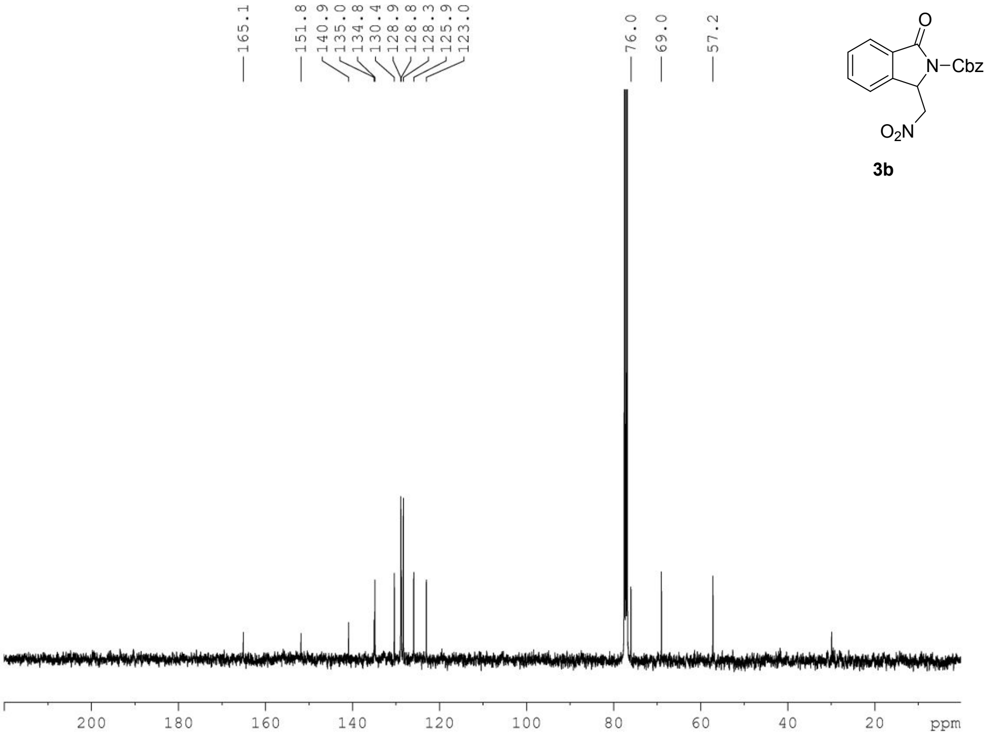

3b

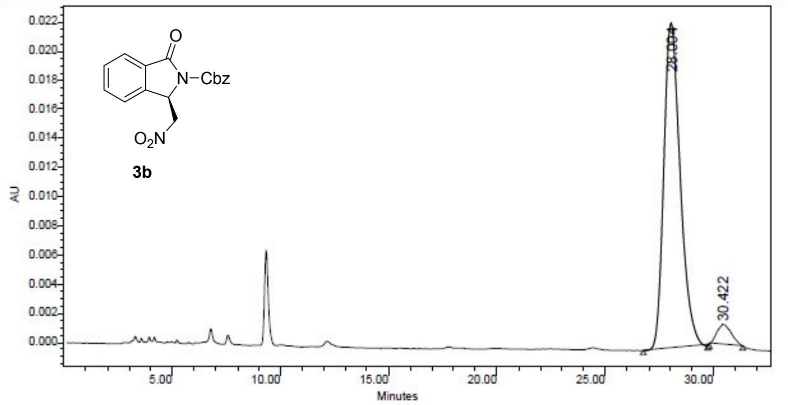

	RT (min)	Area (μV*sec)	% Area	Height (μV)	% Height
1	28.004	1176116	94.66	22321	94.07
2	30.422	66295	5.34	1406	5.93

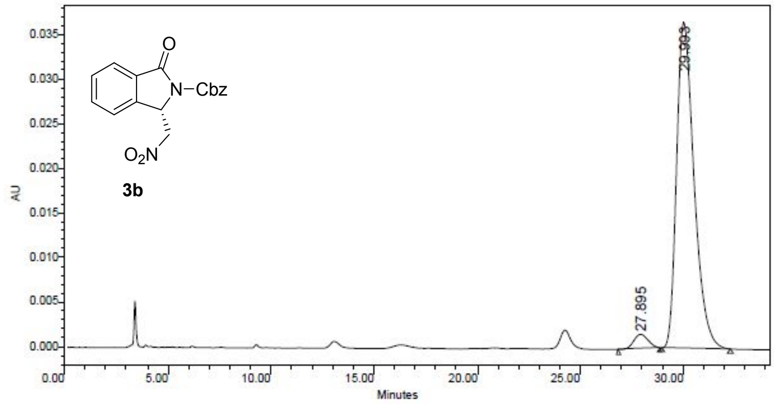

	RT (min)	Area (μV*sec)	% Area	Height (μV)	% Height
1	27.895	78364	3.52	1617	4.24
2	29.993	2148383	96.48	36495	95.76

^{1}H NMR (400 MHz, $CDCl_3$)

^{19}F NMR (400 MHz, $CDCl_3$)

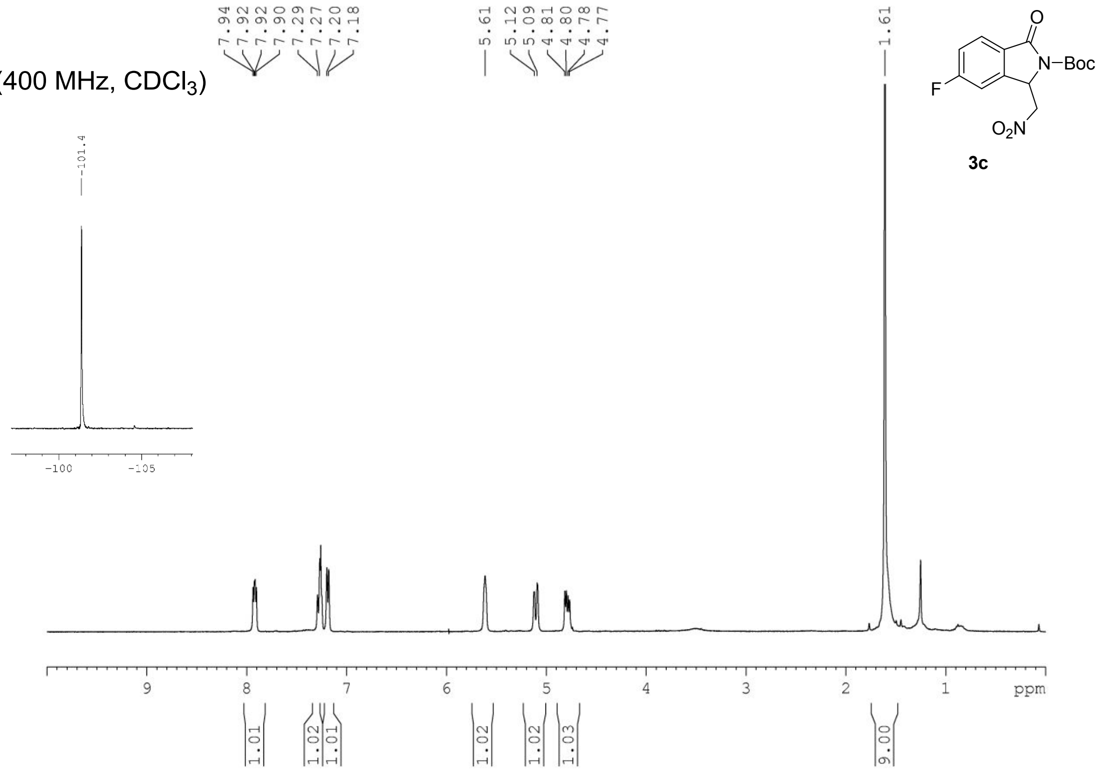

3c

$^{13}C\{^{1}H\}$ NMR (100 MHz, $CDCl_3$)

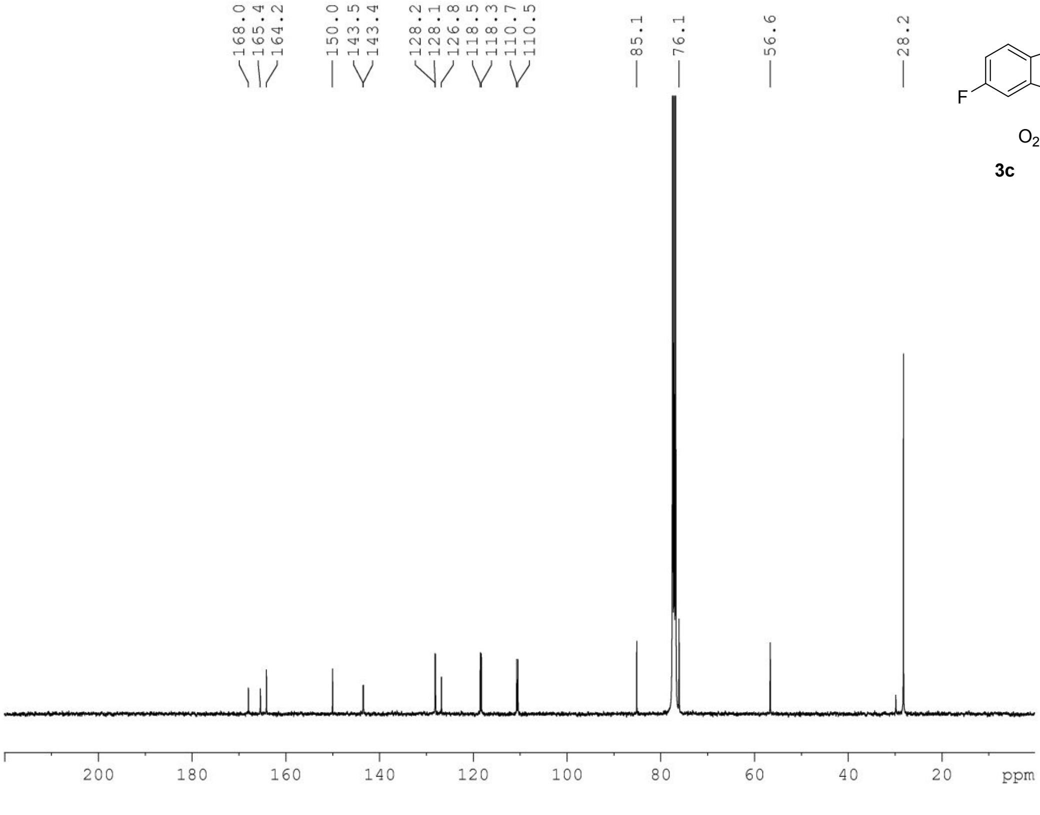

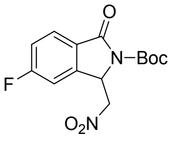

3c

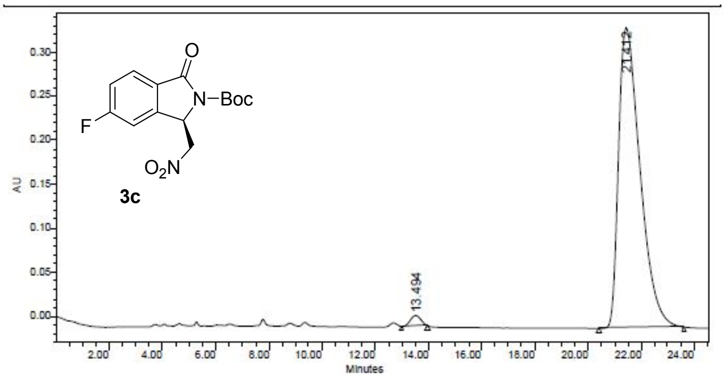

	RT (min)	Area (□V*sec)	% Area	Height (□V)	% Height
1	13.494	342341	1.76	12269	3.48
2	21.412	19150102	98.24	340045	96.52

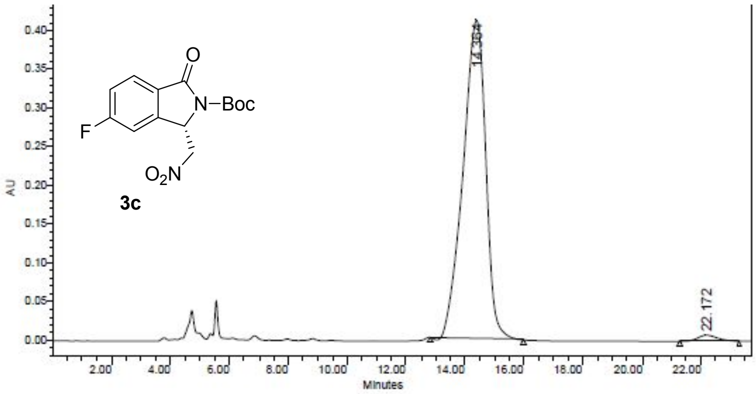

	RT (min)	Area (□V*sec)	% Area	Height (□V)	% Height
1	14.364	22148343	98.39	412021	98.12
2	22.172	361644	1.61	7878	1.88

^{1}H NMR (300 MHz, $CDCl_3$)

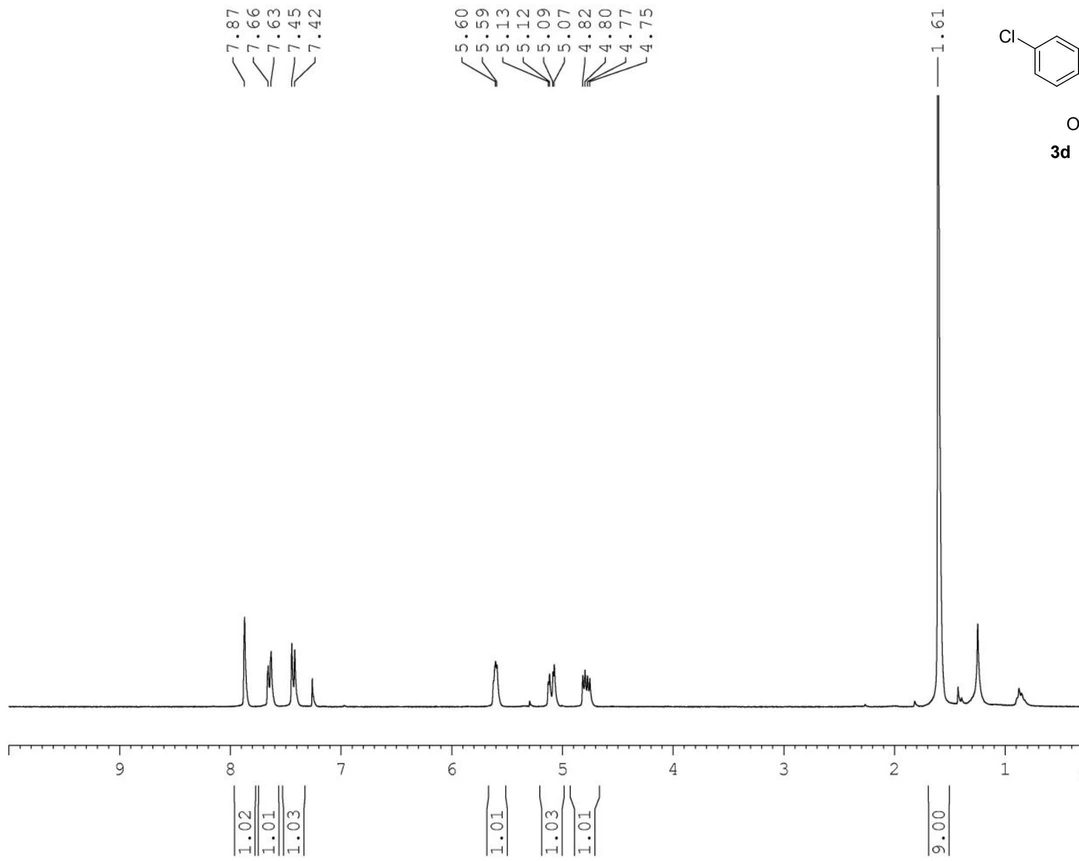

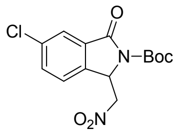

3d

$^{13}C\{^{1}H\}$ NMR (75 MHz, $CDCl_3$)

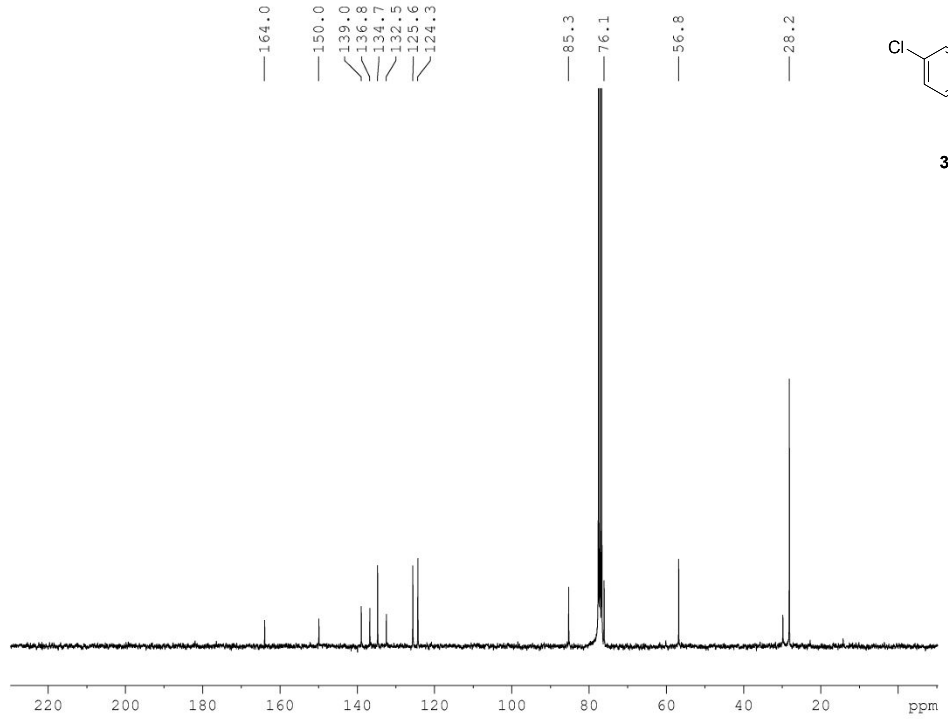

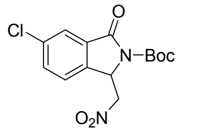

3d

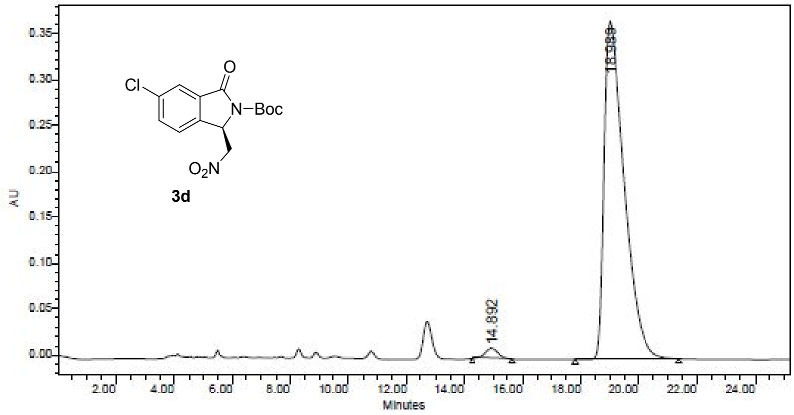

	RT (min)	Area (µV*sec)	% Area	Height (µV)	% Height
1	14.892	344290	1.91	11029	2.92
2	18.989	17681660	98.09	366325	97.08

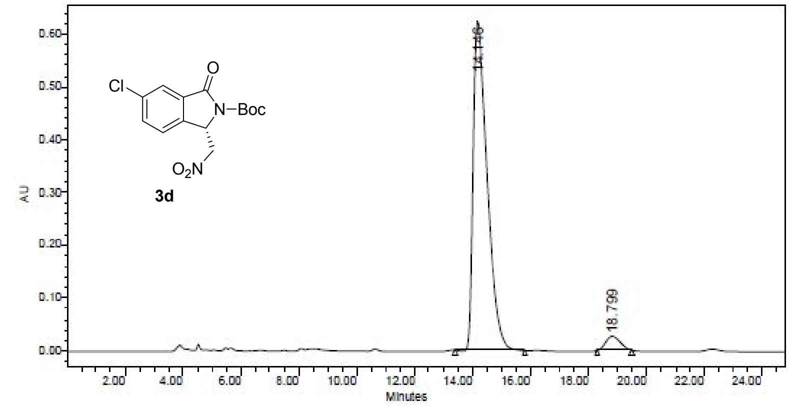

	RT (min)	Area (µV*sec)	% Area	Height (µV)	% Height
1	14.146	20874513	95.74	621863	95.93
2	18.799	929126	4.26	26400	4.07

^{1}H NMR (300 MHz, $CDCl_3$)

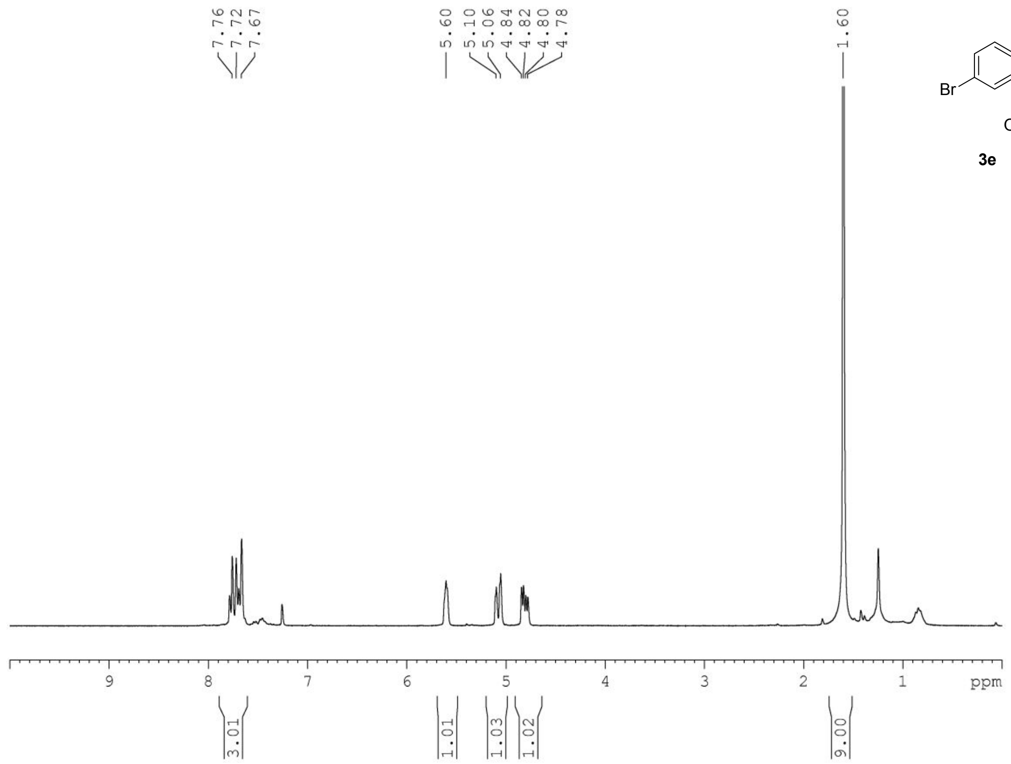

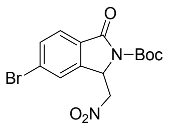

3e

$^{13}C\{^{1}H\}$ NMR (100 MHz, $CDCl_3$)

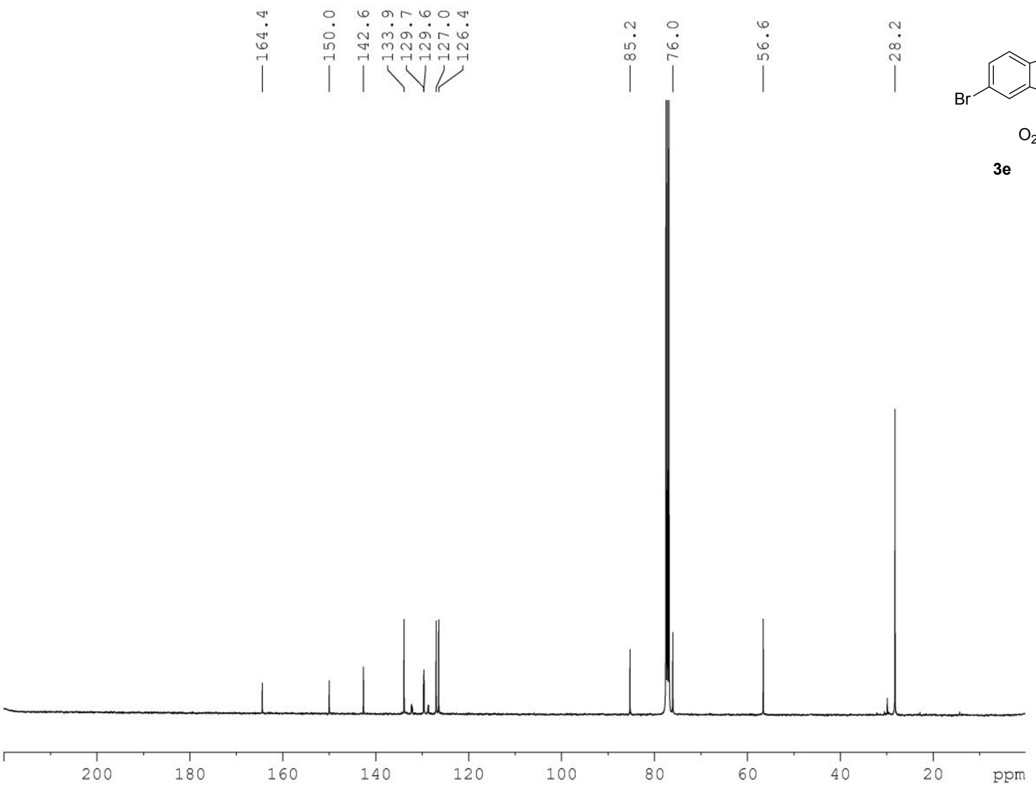

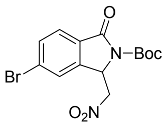

3e

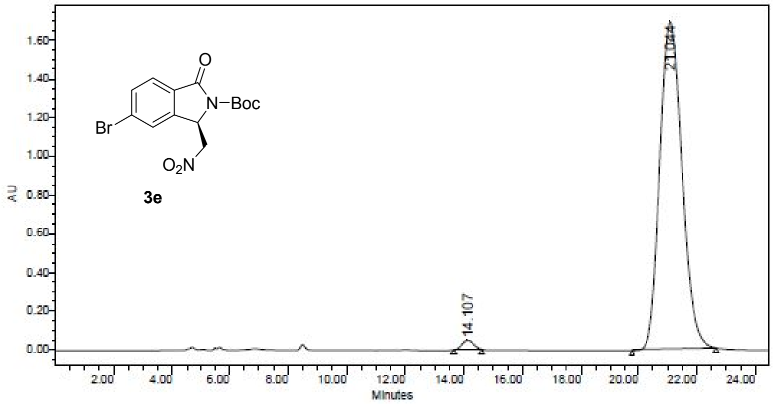

	RT (min)	Area (µV*sec)	% Area	Height (µV)	% Height
1	14.107	1307928	1.44	47336	2.72
2	21.044	89793994	98.56	1694127	97.28

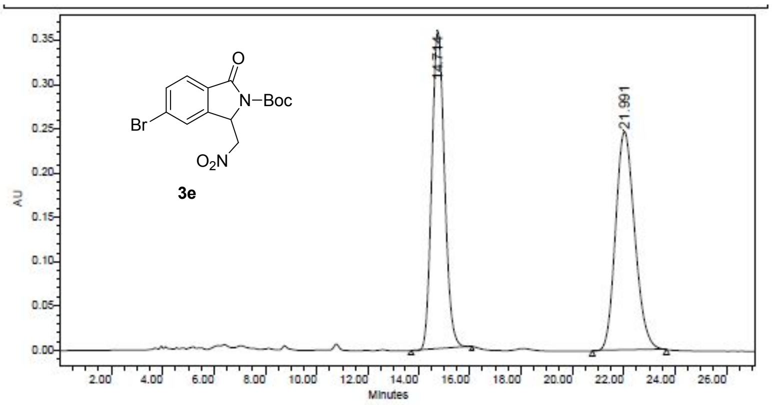

	RT (min)	Area (µV*sec)	% Area	Height (µV)	% Height
1	14.714	12440444	49.10	358610	59.18
2	21.991	12898535	50.90	247340	40.82

^{1}H NMR (300 MHz, $CDCl_3$)

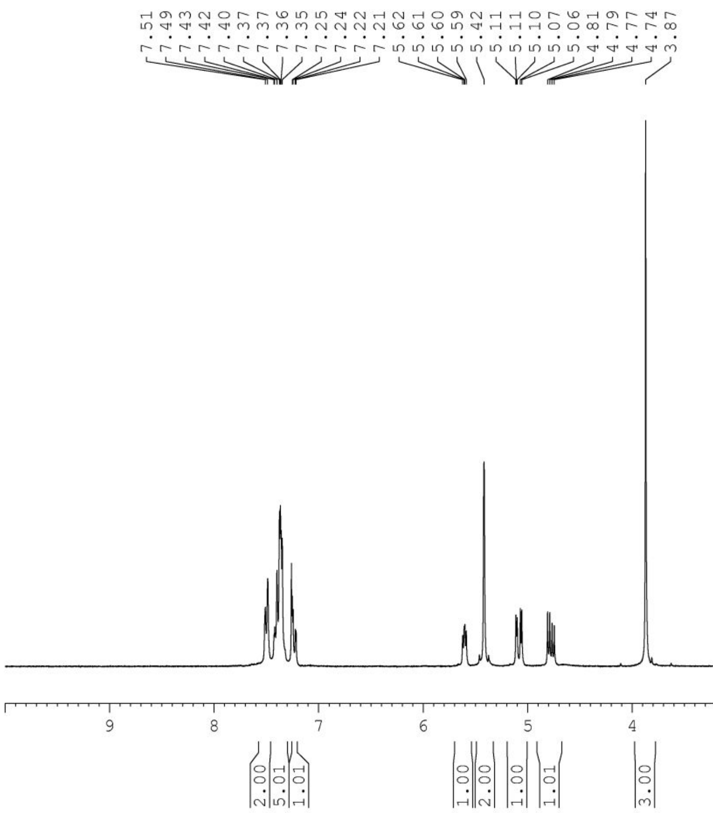

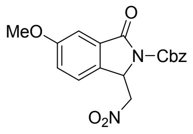

3f

^{13}C{^{1}H} NMR (100 MHz, $CDCl_3$)

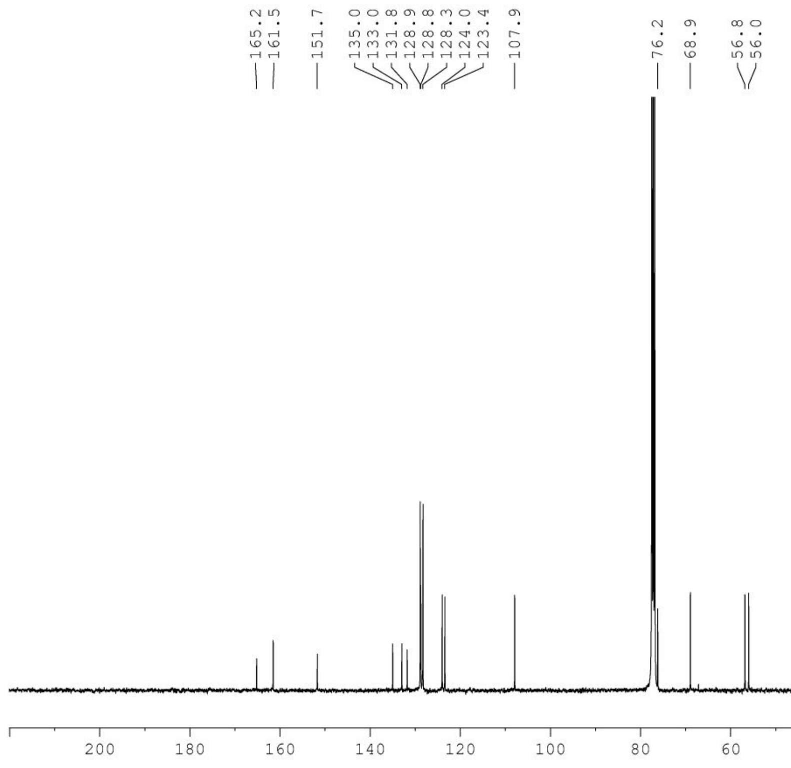

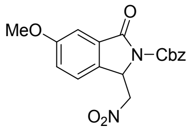

3f

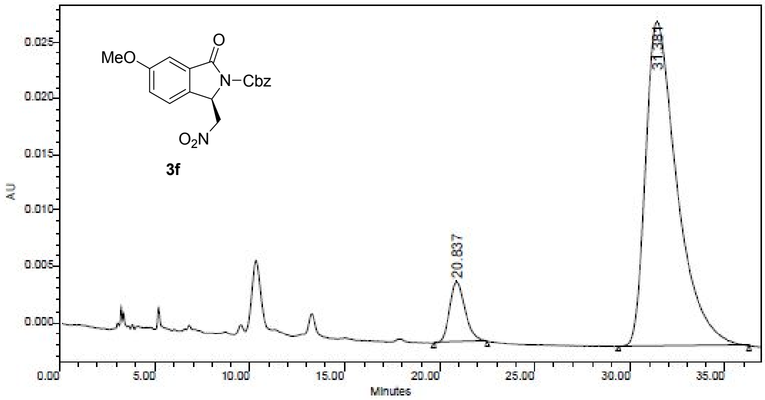

	RT (min)	Area (µV*sec)	% Area	Height (µV)	% Height
1	20.837	308679	8.45	5388	15.69
2	31.381	3346216	91.55	28949	84.31

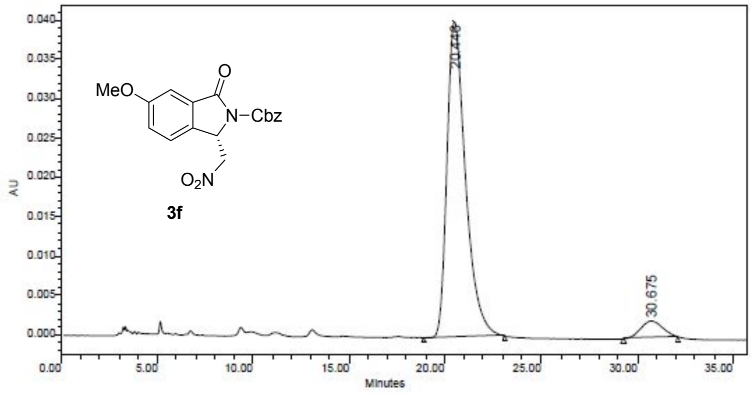

	RT (min)	Area (µV*sec)	% Area	Height (µV)	% Height
1	20.446	2696599	94.02	40101	94.89
2	30.675	171451	5.98	2160	5.11

^1H NMR (400 MHz, $CDCl_3$)

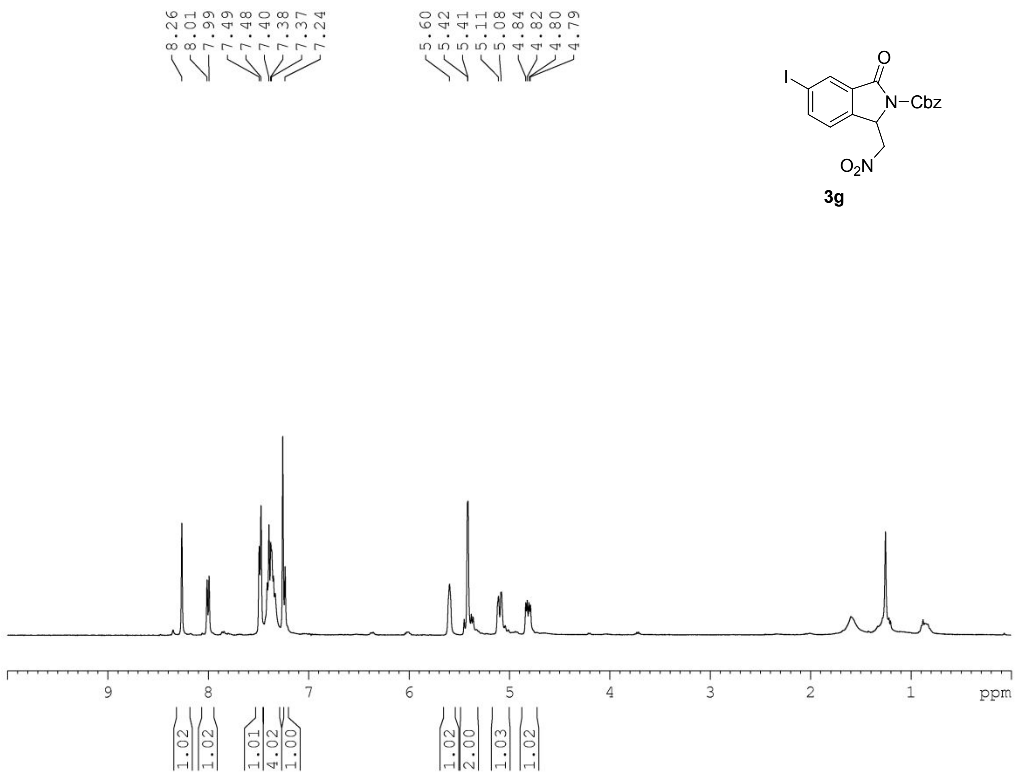

3g

$^{13}C\{^1H\}$ NMR (100 MHz, $CDCl_3$)

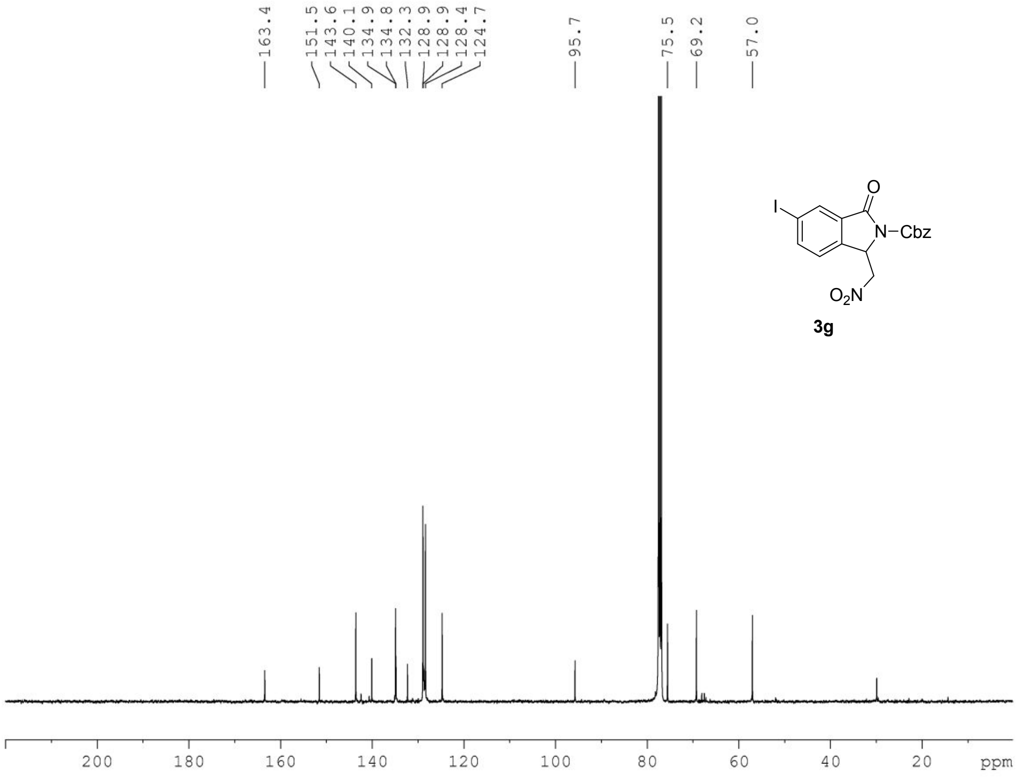

3g

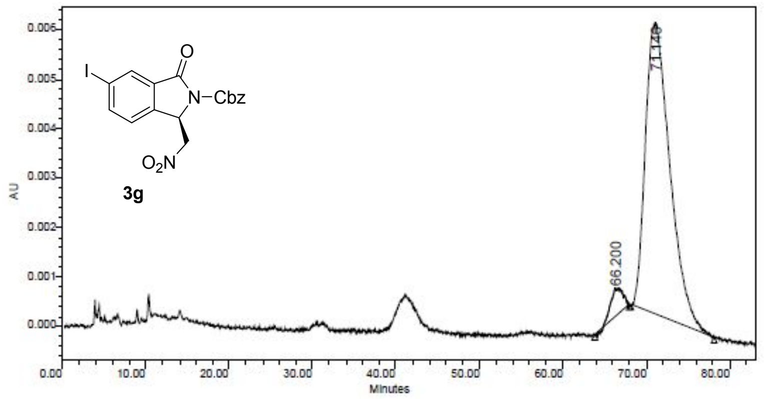

	RT (min)	Area (µV*sec)	% Area	Height (µV)	% Height
1	66.200	61315	4.85	589	9.09
2	71.146	1202866	95.15	5889	90.91

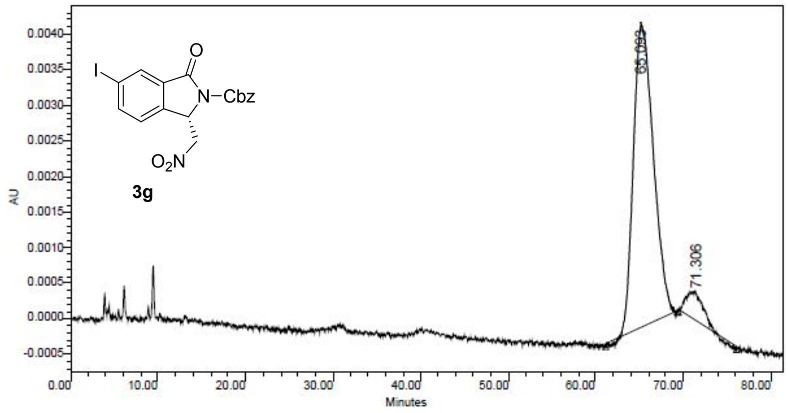

	RT (min)	Area (µV*sec)	% Area	Height (µV)	% Height
1	65.093	682406	92.07	4278	91.35
2	71.306	58791	7.93	405	8.65

^1H NMR (300 MHz, $CDCl_3$)

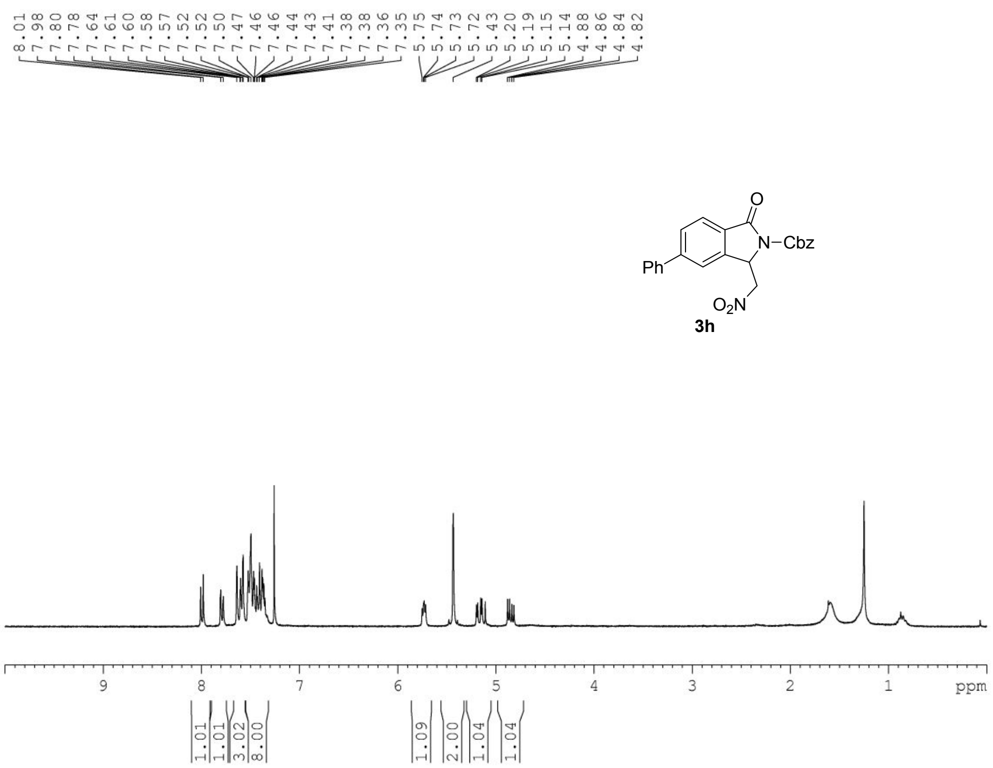

$^{13}C\{^1H\}$ NMR (150 MHz, $CDCl_3$)

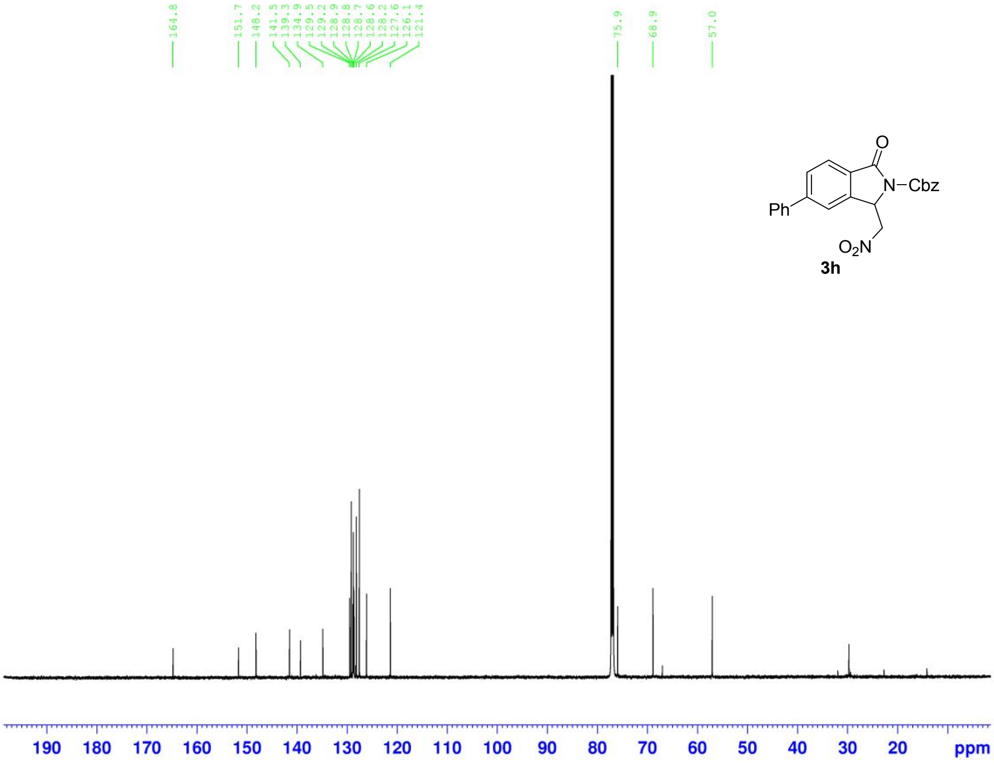

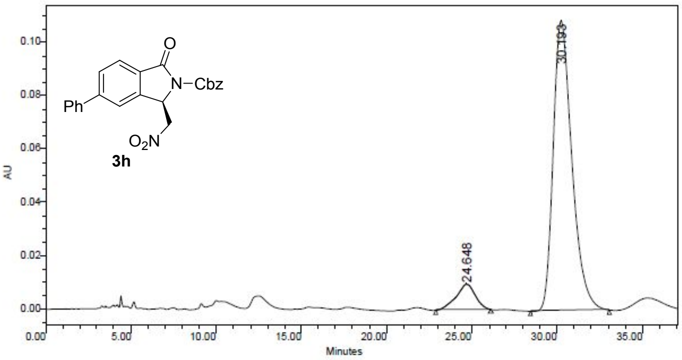

	RT (min)	Area (µV*sec)	% Area	Height (µV)	% Height
1	24.648	763418	8.39	9598	8.11
2	30.193	8330347	91.61	108800	91.89

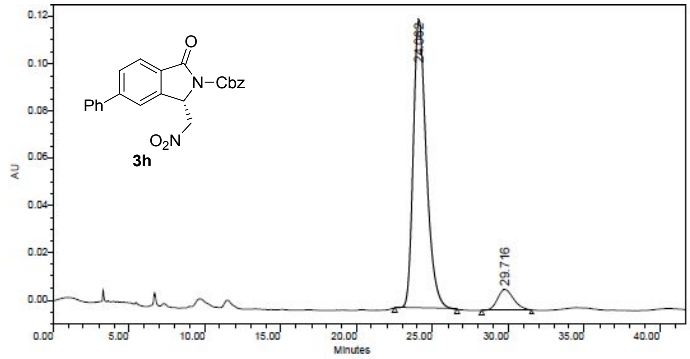

	RT (min)	Area (µV*sec)	% Area	Height (µV)	% Height
1	24.062	7348404	91.83	122201	93.27
2	29.716	653844	8.17	8816	6.73

^1H NMR (400 MHz, $CDCl_3$)

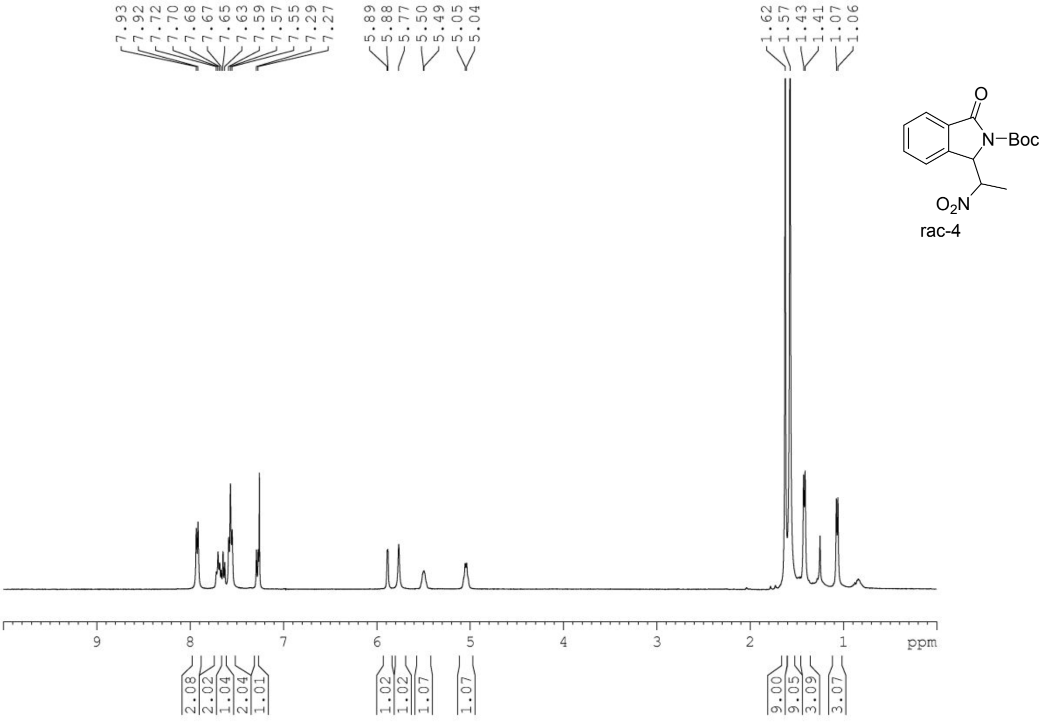

^1H NMR (400 MHz, $CDCl_3$)

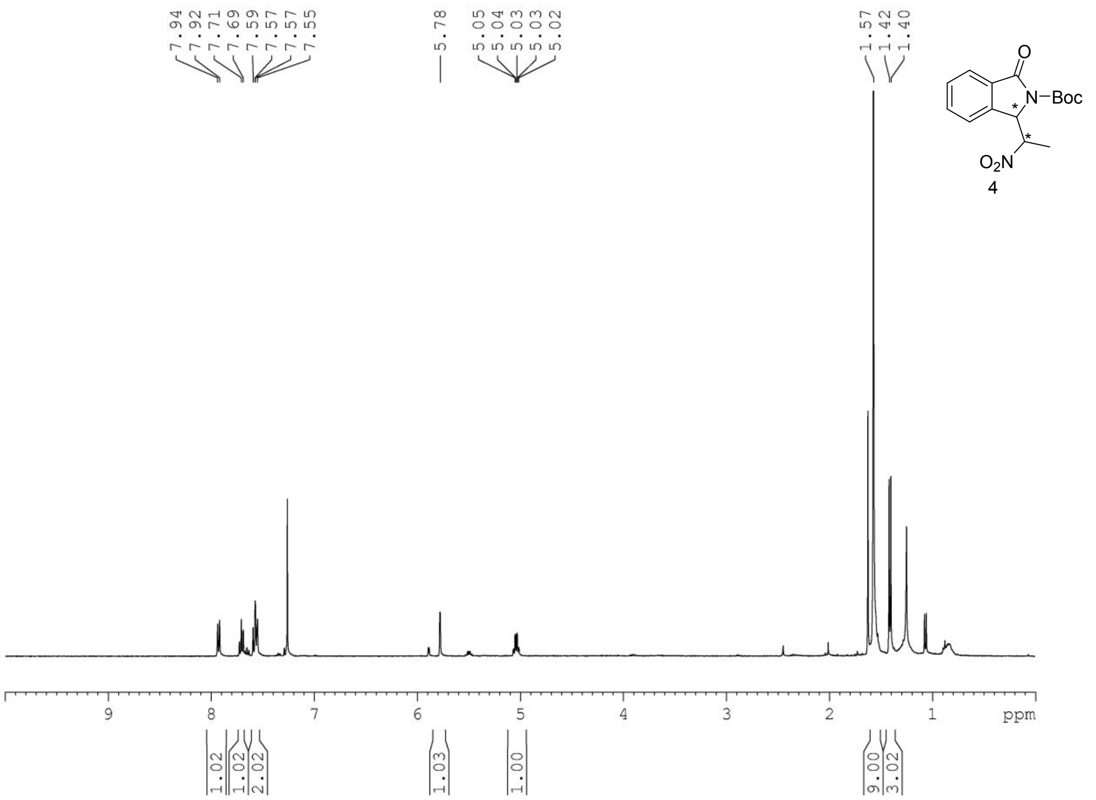

$^{13}C\{^{1}H\}$ NMR (100 MHz, $CDCl_3$)

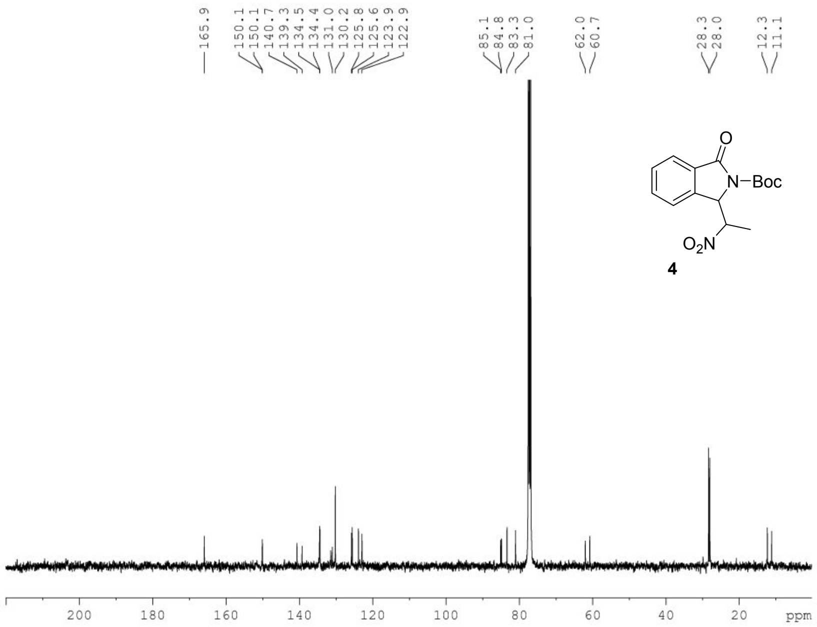

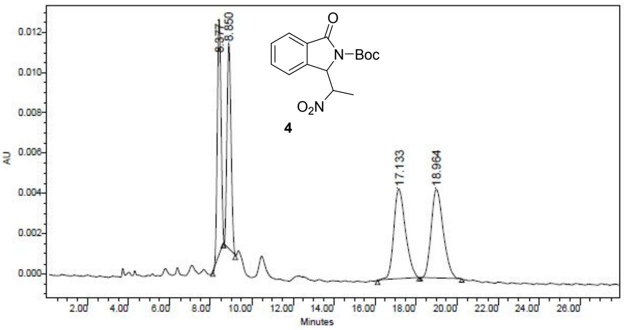

	RT (min)	Area (µV*sec)	% Area	Height (µV)	% Height
1	8.377	151004	23.23	11813	38.30
2	8.850	139680	21.49	10136	32.86
3	17.133	178909	27.52	4486	14.54
4	18.964	180434	27.76	4408	14.29

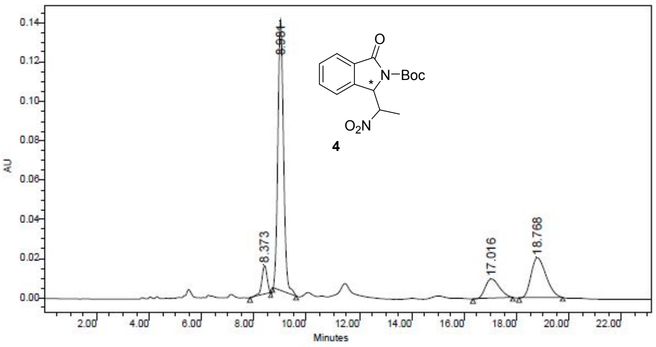

	RT (min)	Area (µV*sec)	% Area	Height (µV)	% Height
1	8.373	211591	6.12	14791	8.10
2	8.981	2059134	59.55	137533	75.30
3	17.016	373675	10.81	9891	5.42
4	18.768	813270	23.52	20423	11.18

^1H NMR (300 MHz, $CDCl_3$)

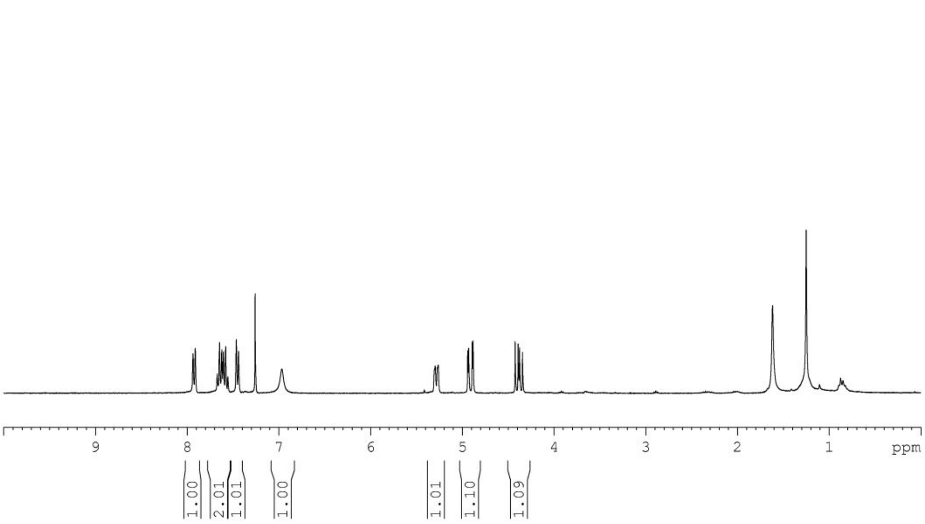

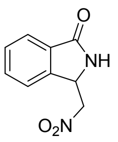

5

$^{13}C\{^1H\}$ NMR (75 MHz, DMSO-d_6)

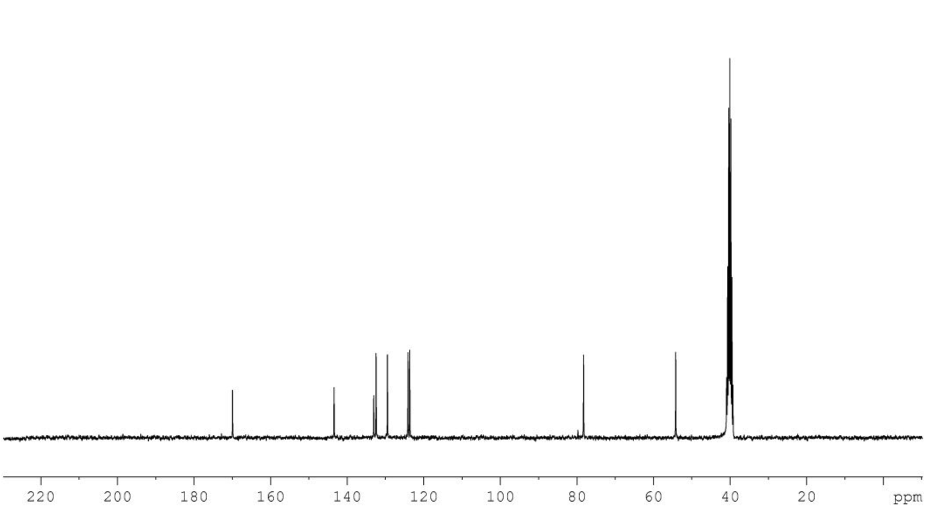

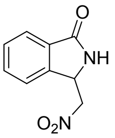

5

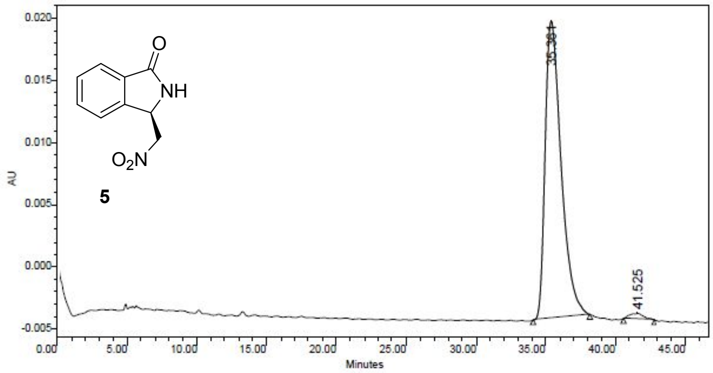

	RT (min)	Area (µV*sec)	% Area	Height (µV)	% Height
1	35.361	1908574	98.21	23936	98.05
2	41.525	34824	1.79	475	1.95

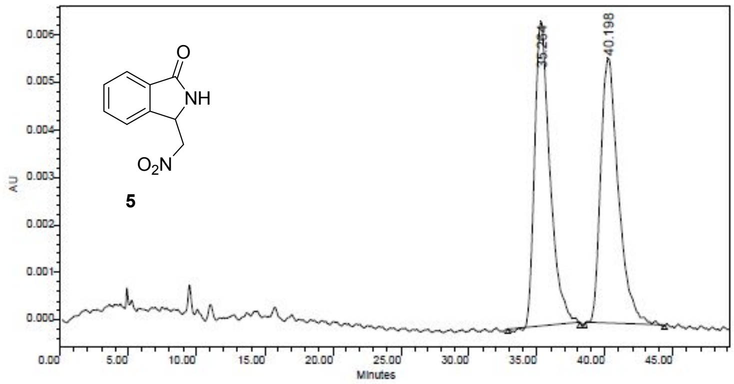

	RT (min)	Area (µV*sec)	% Area	Height (µV)	% Height
1	35.264	508788	50.00	6425	53.47
2	40.198	508871	50.00	5592	46.53

^{1}H NMR (400 MHz, $CDCl_3$)

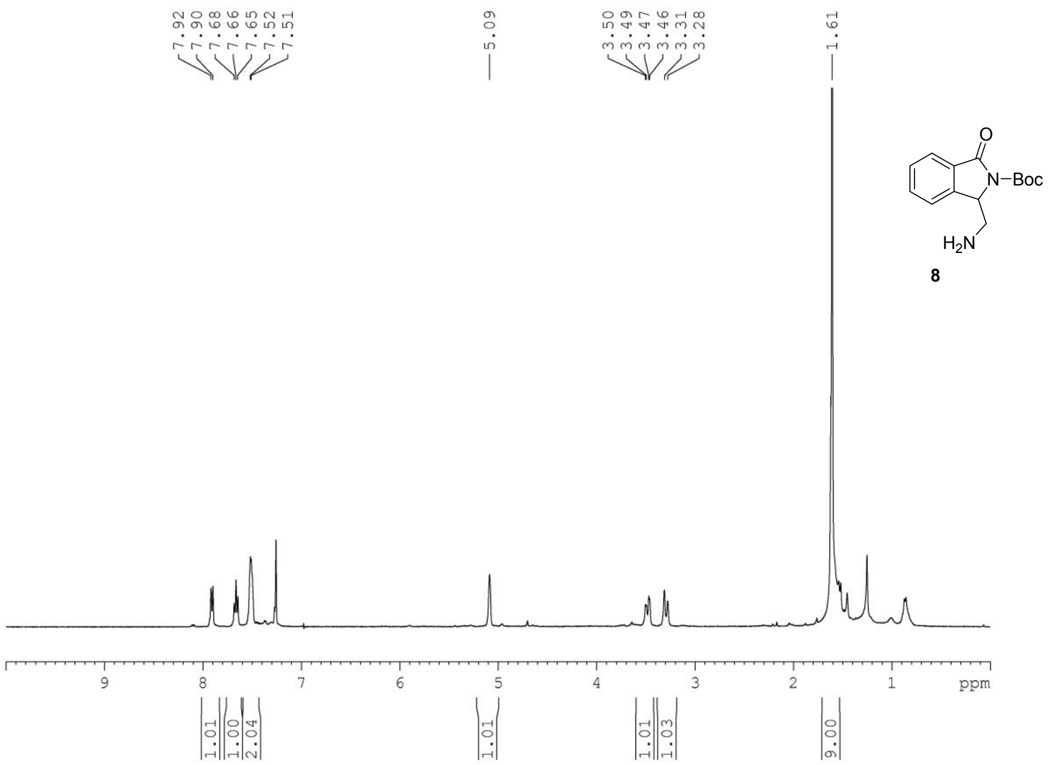

8

$^{13}C\{^{1}H\}$ NMR (100 MHz, $CDCl_3$)

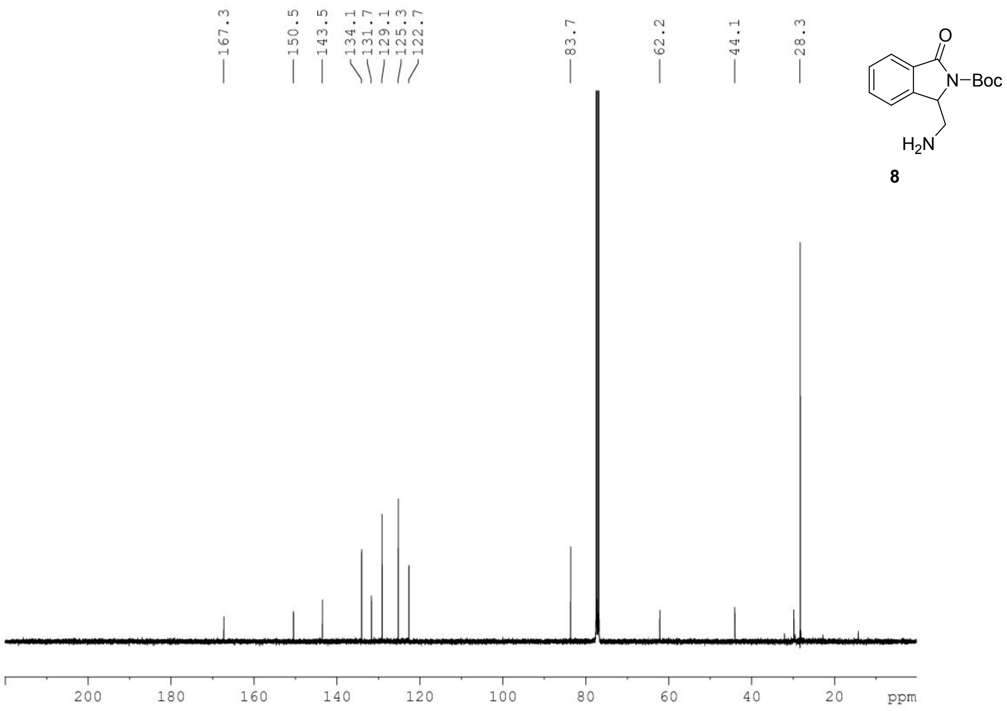

8

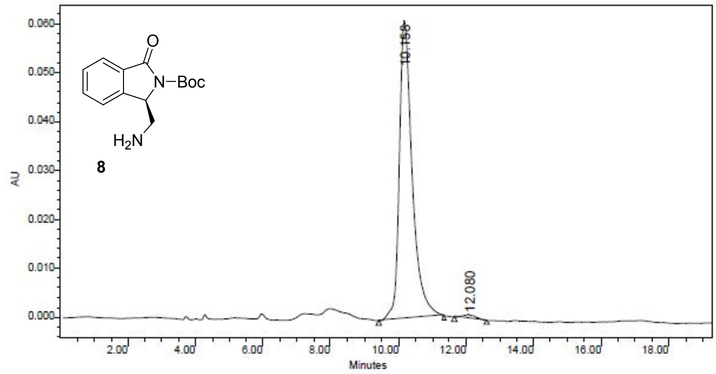

	RT (min)	Area (μV*sec)	% Area	Height (μV)	% Height
1	10.158	1619523	98.70	60885	98.74
2	12.080	21311	1.30	774	1.26

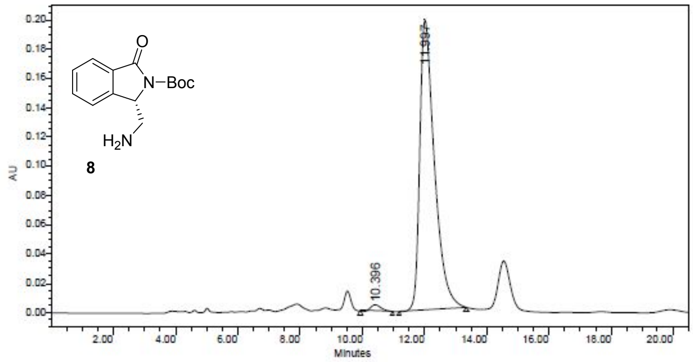

	RT (min)	Area (μV*sec)	% Area	Height (μV)	% Height
1	10.396	109119	1.65	4566	2.25
2	11.997	6512081	98.35	198501	97.75

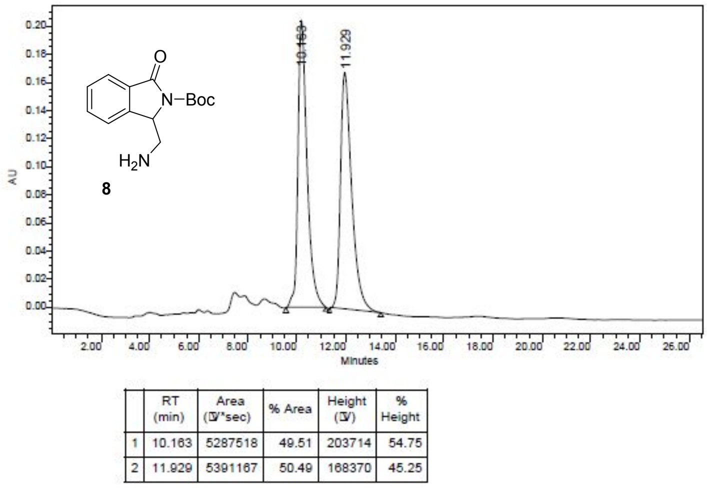

	RT (min)	Area (μV*sec)	% Area	Height (μV)	% Height
1	10.163	5287518	49.51	203714	54.75
2	11.929	5391167	50.49	168370	45.25

^{1}H NMR (300 MHz, $CDCl_3$)

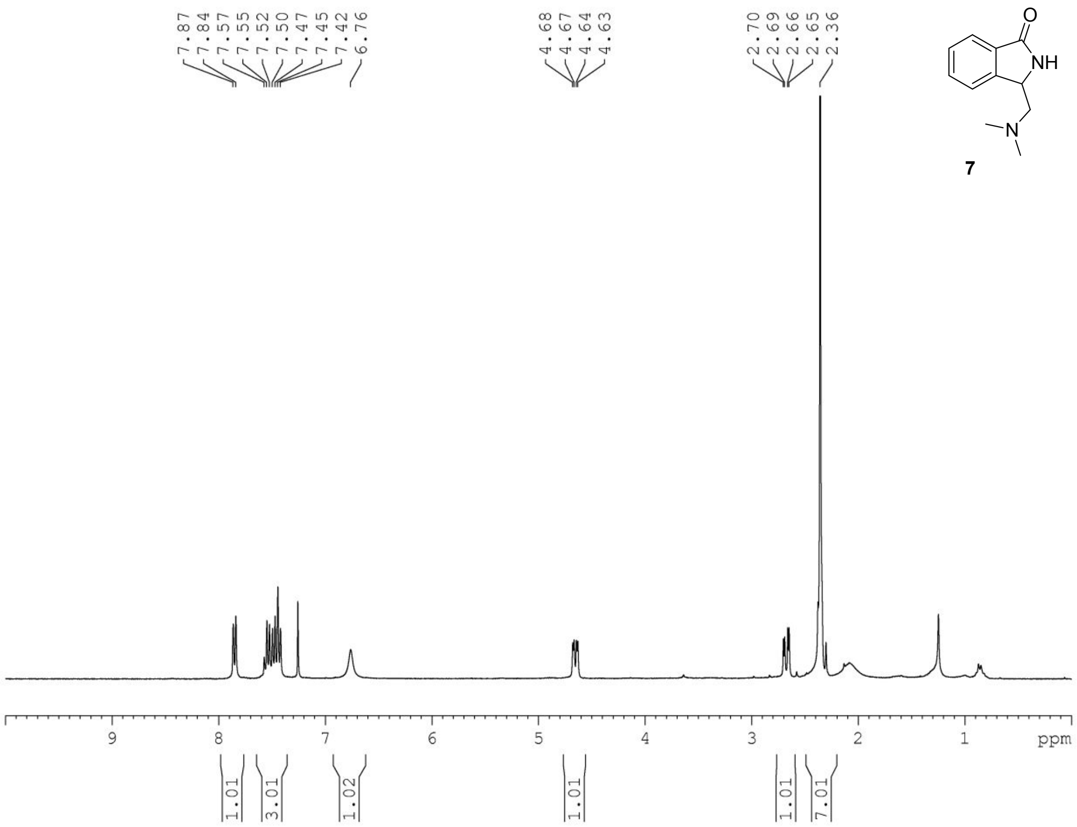

$^{13}C\{^{1}H\}$ NMR (75 MHz, $CDCl_3$)

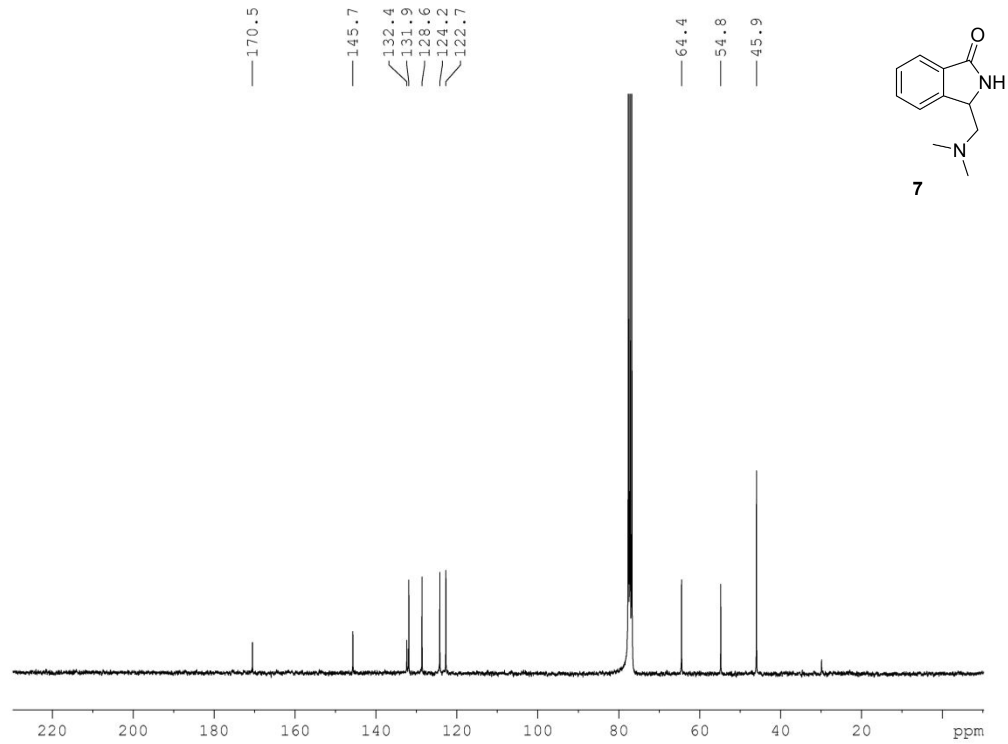

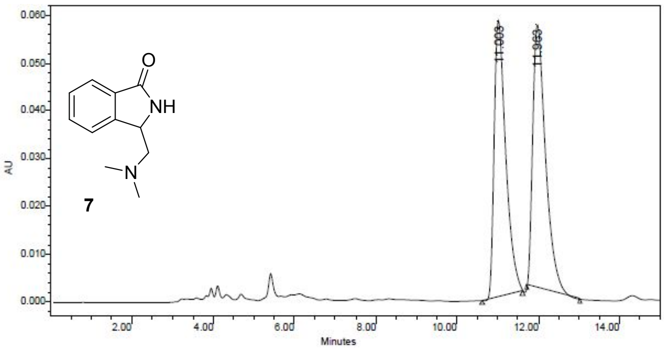

	RT (min)	Area (μV*sec)	% Area	Height (μV)	% Height
1	11.003	1146226	48.78	57883	51.27
2	11.963	1203412	51.22	55019	48.73

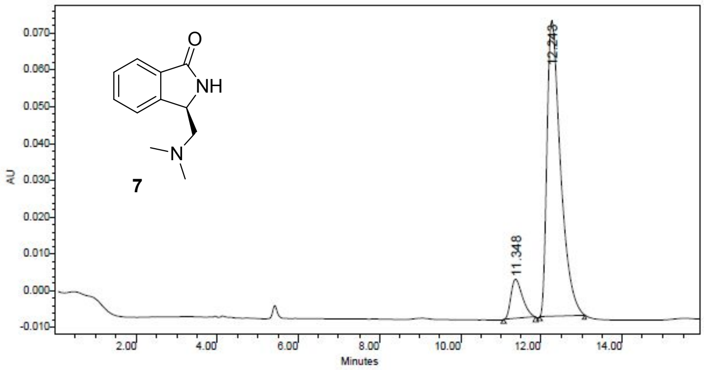

	RT (min)	Area (μV*sec)	% Area	Height (μV)	% Height
1	11.348	216601	10.36	10767	11.80
2	12.243	1873864	89.64	80454	88.20

IR spectra of selected compounds

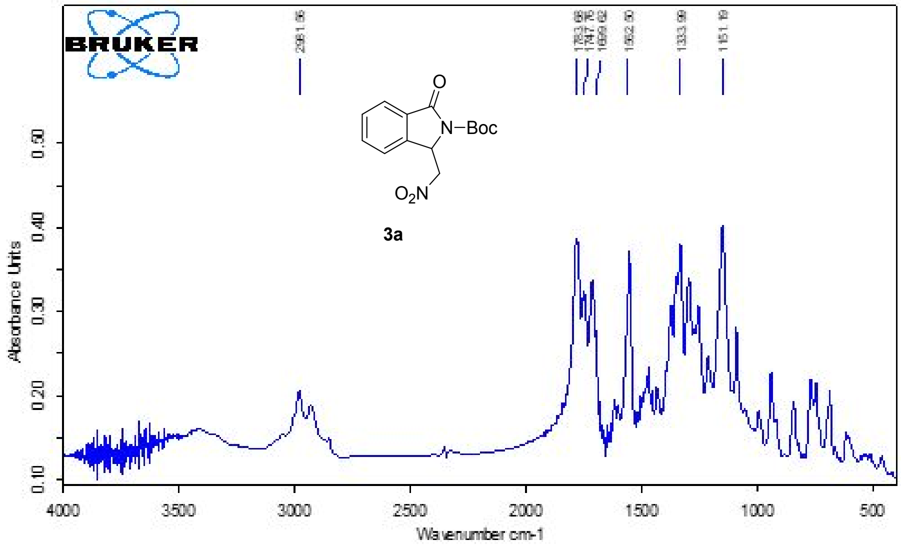

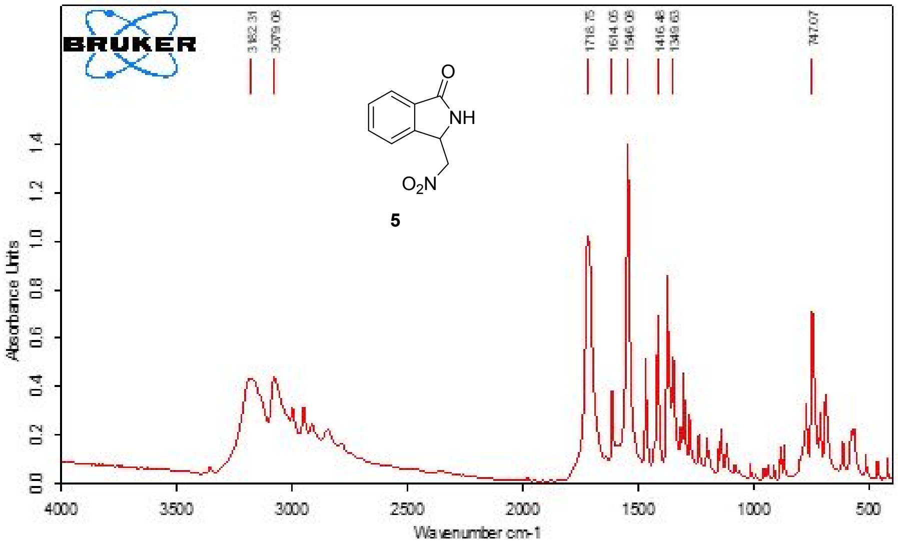

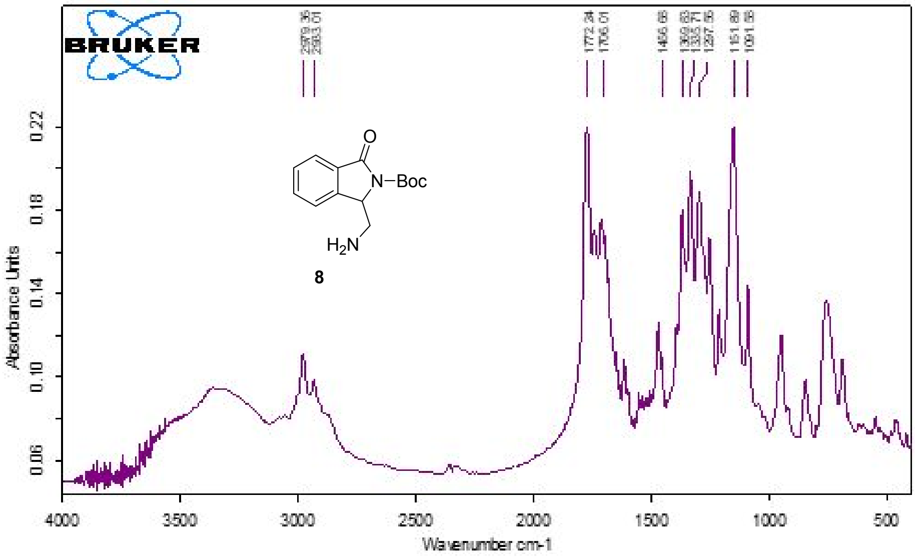

Supplement: Supplementary file 1 — jo2c00518_si_001.pdf [file jo2c00518_si_001.pdf]
